# Electro‐Oxidative Selective Esterification of Methylarenes and Benzaldehydes

**DOI:** 10.1002/chem.202005158

**Published:** 2021-01-25

**Authors:** Congjun Yu, Bünyamin Özkaya, Frederic W. Patureau

**Affiliations:** ^1^ Institute of Organic Chemistry RWTH Aachen University Landoltweg 1 52074 Aachen Germany

**Keywords:** electro-oxidative acetalization, electro-oxidative coupling, electro-oxidative esterification, oxidative esterification, phosphonium electrolytes

## Abstract

A mild and green electro‐oxidative protocol to construct aromatic esters from methylarenes and alcohols is herein reported. Importantly, the reaction is free of metals, chemical oxidants, bases, acids, and operates at room temperature. Moreover, the design of the electrolyte was found critical for the oxidation state and structure of the coupling products, a rarely documented effect. This electro‐oxidative coupling process also displays exceptional tolerance of many fragile easily oxidized functional groups such as hydroxy, aldehyde, olefin, alkyne, as well as neighboring benzylic positions. The enantiomeric enrichment of some chiral alcohols is moreover preserved during this electro‐oxidative coupling reaction, making it overall a promising synthetic tool.

## Graphical abstract

**P beats N**: A mild and green electro‐oxidative protocol to construct aromatic esters from methylarenes and alcohols is reported. Importantly, the reaction has a broad functional group tolerance and is free of metals, chemical oxidants, bases, acids, while operating at room temperature. The design of the electrolyte was found critical for the oxidation state and structure of the coupling products.

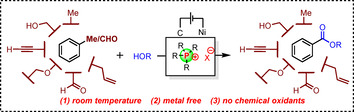

Electro‐oxidative strategies occupy an increasingly important place in the synthetic method toolbox. Indeed, the vast majority of organic building blocks possess numerous C−H bonds, making electro‐oxidation a privileged and arguably still underappreciated tool for the design of step and atom economic coupling reactions. In this context, carboxyl esters are among the most prevalent functional groups encountered in very diverse areas of organic chemistry, from liquid crystal polymers, to cosmetics, pharmaceuticals, artificial fragrances, agrochemicals and food additives.^[1]^ Moreover, they constitute a relatively straight‐forward retrosynthetic disconnection. Indeed, traditional methods of esterification are typically accomplished by reacting carboxylic acids or their activated derivatives (e.g. acyl chlorides, anhydrides and activated esters) with alcohols and related substrates.^[2]^ In those cases, pre‐oxidation and pre‐activation are thus necessary, usually associated with strong chemical oxidants and their typically poor functional group tolerance. Moreover, the strong acids or bases typically employed in those methods further limit the associated substrate scopes. Alternative routes through the oxidative esterification of aldehydes^[3]^ and benzyl alcohols^[4]^ have also been developed. However, such methodologies generally still require stoichiometric amounts of strong and toxic oxidants, high temperature and/or transition‐metal catalysts (Scheme 1 a). Thus, in this context, the concept of direct oxidative esterification of methylarenes with ubiquitous alcohols still constitutes a priority objective. Such a strategy can be implemented by using harsh reaction conditions with metal catalysts, strong chemical oxidants, and high reaction temperatures (Scheme 1 b).^[5]^

**Scheme 1:**
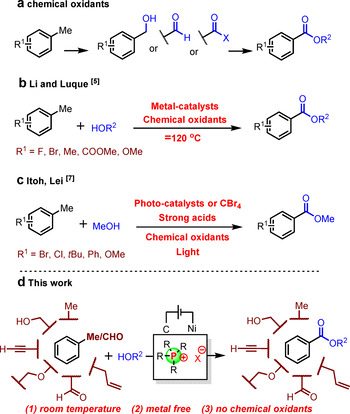
The oxidative construction of esters: a) chemical oxidants; b) Li, Luque, and colleagues;^[5]^ c) Itoh, Lei, and co‐workers;^[7]^ d) this work.

Inspiring flow chemistry techniques were moreover applied, such as the electrochemical oxidative esterification of 4‐methoxytoluene in a flow reactor.^[6]^ Alternatively, photocatalytic methods have also been developed, however often relying on onerous photocatalysts, stoichiometric amounts of CBr_4_ or strong acids. Moreover, in those cases, strong chemical oxidants remain indispensable. Thus, the corresponding substrates scopes tend to be limited, in particular toward fragile easily oxidized functional groups such as hydroxy, ether, aldehyde as well as other benzylic positions (Scheme 1 c).^[7]^ Herein, we report a mild and green protocol to construct esters from methylarenes and alcohols with electricity^[8]^ as the only „oxidant.“ This electro‐oxidative method is associated with a broad fragile functional group tolerance (Scheme 1 d).

We started our investigations from 4‐methylanisole **1 a** as the model substrate reacting with methanol **1 b** in an undivided cell (pictures shown in Figure 1). Carbon was used as the anode, while nickel was found to be the best cathode.^[9]^ Three products can be formed in this reaction: the target oxidative coupling product ester **1 c**, the aldehyde **1 d** and the acetal **1 e**. Diverse electrolytes were tested under several currents showing a significant impact on the transformation's selectivity with respect to these three products. As expected, lower currents were found to favor acetalization. In contrast, higher currents favor the esterification product, suggesting the intermediacy of **1 e** and/or **1 d** in the formation of ester coupling product **1 c**. In all cases, the optimal reaction time was found to be 18 h for reaching full conversion.

**Figure 1:**
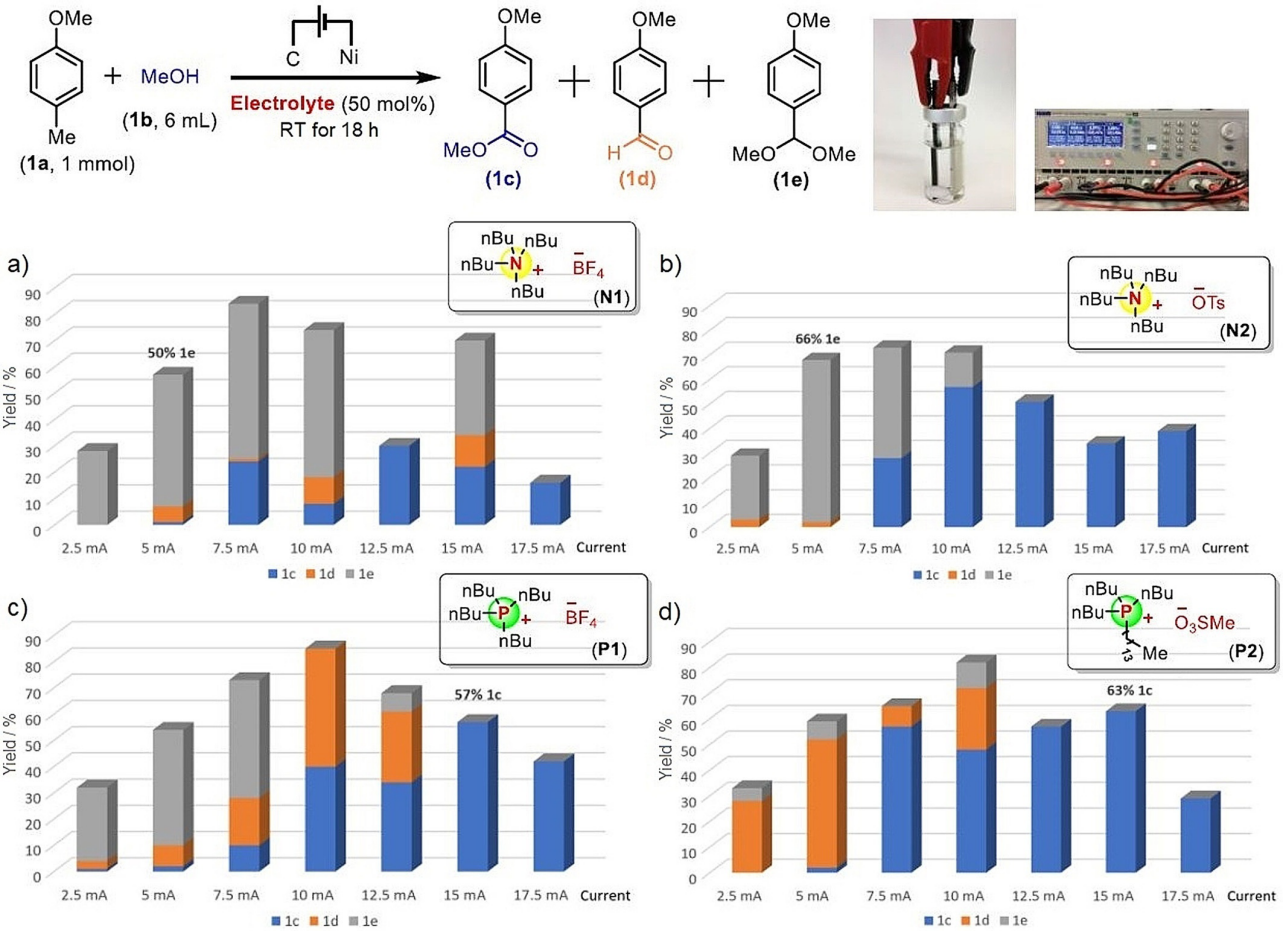
Electro‐oxidative product ratios, depending on electrolytes and currents. Yields determined by ^1^H NMR analysis of the crude reaction mixture with 1,3,5‐trimethoxybenzene as an internal standard.

Importantly, the electrolyte seems to have an influence on the electro‐oxidative coupling reaction's outcome. The common ammonium electrolytes favor the formation of acetals (Figure 1 a,b). The best conditions for acetalization were obtained with tetrabutylammonium *p*‐toluenesulfonate **N2** under a 5 mA current, giving a 66 % NMR yield of acetal **1 e**. In contrast, the phosphonium salts, which are rarely used as electrolytes, favor the esterification process (Figure 1 c,d). The best conditions for esterification were obtained with tributyl(tetradecyl)phosphonium methanesulfonate **P2** as the electrolyte under a current of 15 mA, providing a 63 % NMR yield of ester **1 c**. Thus, the electro‐oxidative coupling reaction's outcome seems to depend on the electrolyte's structure, phosphonium salts leading to higher oxidation than ammonium salts (**P2**>**P1**>**N2**>**N1**), and sulfonate anion leading to higher oxidation than tetrafluoroborate.

To investigate the electrolyte's influence, a series of initial mechanistic experiments were conducted. Firstly, in situ NMR of the crude reaction mixture showed that neither representative electrolytes **N1** nor **P2** were chemically altered during the reaction. Thus, a chemical involvement of the electrolyte can be reasonably excluded. The cyclic voltammetry (CV) profiles of **N1** and **P2** were then measured with Ferrocene as an internal standard (Figure 2 a–d). Interestingly, the phosphonium electrolyte **P2** showed a clear oxidation potential above +2 V, while the ammonium electrolyte **N1** did not. Next, the cyclic voltammograms of methylarene substrate **1 a** and the two subsequent reaction intermediates acetal **1 e** and aldehyde **1 d** were measured in combination with both reference electrolytes **N1**, and thereafter **P2**. Those seem to follow the oxidation potential trend: **1 a**<**1 e<1 d**. Moreover, interestingly, electrolyte **P2** seems to make the oxidation potentials of these compounds globally higher than electrolyte **N1**. This may explain why ammonium electrolytes tend to favor acetal product **1 e**, while phosphonium electrolyte **P2** may be more suitable for further oxidative esterification toward product **1 c**. The precise mechanistic principles responsible for the latter electrolyte effects remain unclear however, and are being further studied in our laboratory. Diverse alternative electrolytes, electrodes as well as diverse reaction conditions have also been investigated, none of which provided superior results, whether toward acetalization or esterification (see SI).

**Figure 2:**
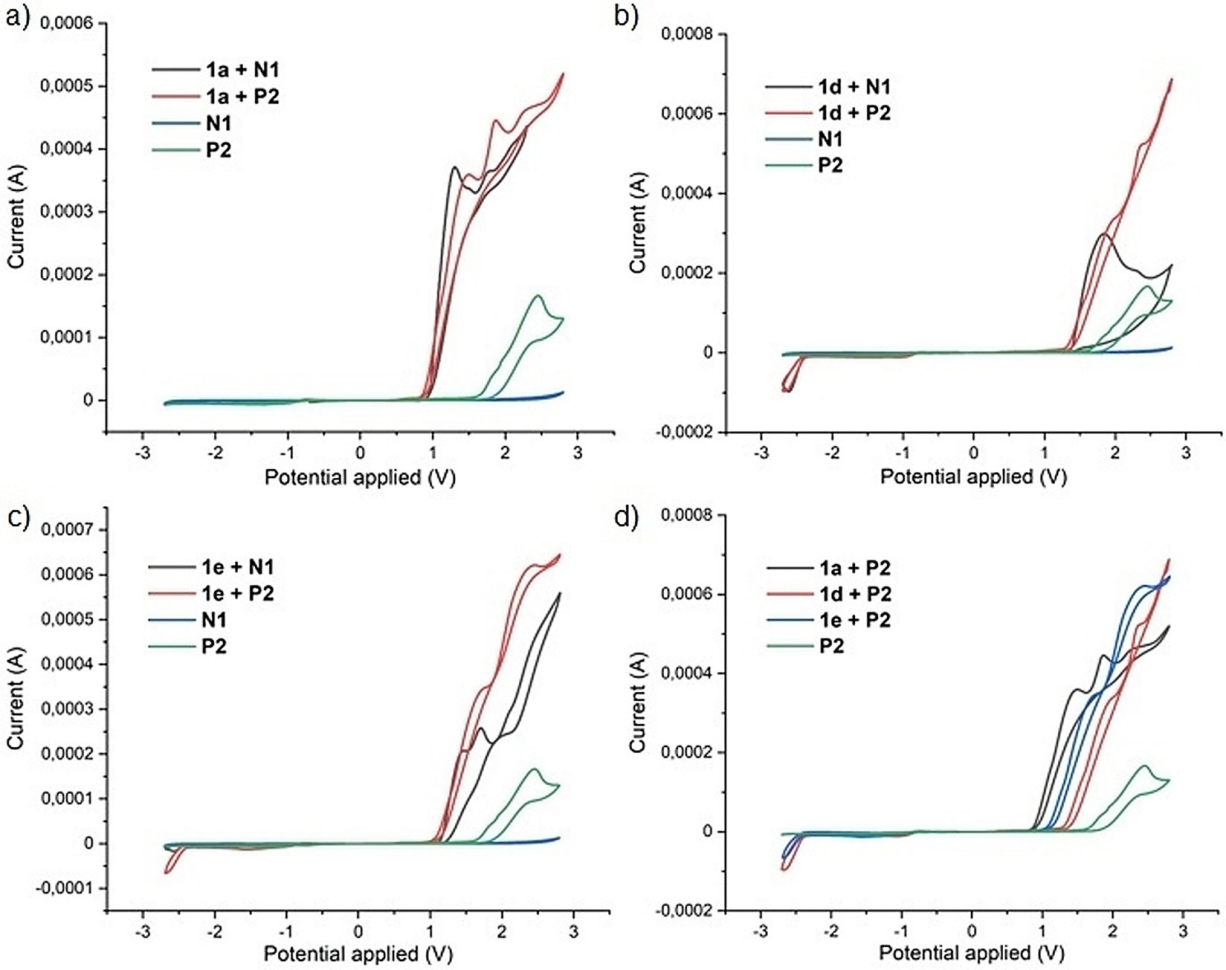
Cyclic voltammograms (CV), with a Pt disk working electrode (diameter 1 mm), Ag/Ag^+^ (0.01 m AgNO_3_, 0.1 m NBu_4_PF_6_, MeCN) as the reference electrode, and a Pt wire counter electrode. Scan rate: 100 mV s^−1^, solvent: MeCN. All investigated substrates (**1 a**, **1 d**, **1 e**) and electrolytes (**N1**, **P2**) were engaged at 1 m concentration. Ferrocene was used as internal standard.

The electro‐oxidative esterification substrate scope was thereafter investigated. The *para* position with respect to the methylarene reaction site proved to be important, with *para*‐methoxy‐ and *para*‐bromo‐ providing superior results. This particularity allows for remarkable benzylic group tolerance on the other (non‐reactive) positions of the arene substrate (Table 1, **3 c**, **5 c**, **7 c**, **9 c**, **10 c**). This reaction is also remarkably tolerant to a number of other oxidation sensitive functional groups such as aldehyde (Table 1, **11 c**), ethers (Table 1, **13 c**, **15 c**) and hydroxy groups (Table 1, **16 c**). This appreciable redox tolerance toward fragile functional groups highlights the superiority of electro‐oxidative strategies versus chemical oxidants. Other important groups such as furan (Table 1, **14 c**), fluoride (Table 1, **17 c**, **19 c**) and chloride (Table 1, **20 c**) are also tolerated in this reaction. Many alcohols have also been found to be effective coupling partners for the reaction (Table 1, **21 c**–**32 c**). For instance, (*R*)‐(−)‐1‐Methoxy‐2‐propanol (**31 b**) and (*R*)‐(+)‐1‐Phenylethanol (**32 b**) were successfully used in the reaction, while maintaining their enantiomeric enrichment (**31 c** was obtained with 96 % *ee* and **32 c** with 98 % *ee*).

**Table 1:** Substrate scope for the electro‐oxidative esterification of methylarenes.^[a]^

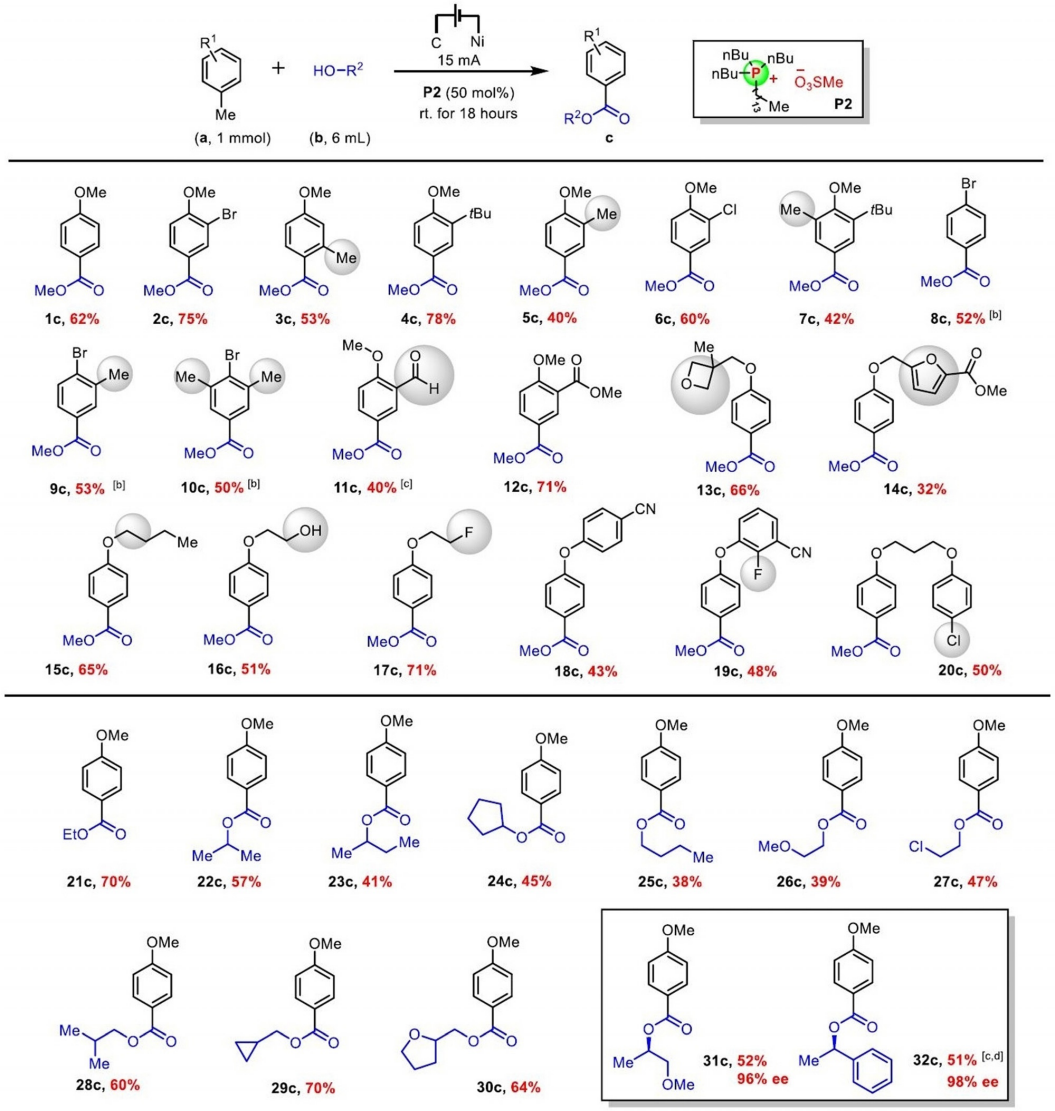

[a] Isolated yields. [b] Electrolyte **N1** was used instead of **P2**. [c] 1.0 equivalent electrolyte was applied. [d] The current was 10 mA.

The electro‐oxidative acetalization reaction was thereafter investigated. Acetalization is a useful synthetic technique to protect aldehydes of various multifunctional organic molecules.^[10]^ Besides, acetals are also important organic synthons and intermediates as well as common functional group in natural products.^[11]^ In the fine chemical industries, acetals are also widely used.^[12]^ Traditional routes to acetalization usually start from aldehydes,[^10^, ^13^] alcohols^[14]^ or olefins.^[15]^ Starting from methylarenes has been less reported.^[16]^ Therefore, we investigated the scope of the herein described electro‐oxidative acetalization of methylarenes (Table 2), with suitable electrolytes. For electron‐rich arenes such as **1 a**, the acetalization was achieved under a low 5 mA current. In other cases, a higher current was needed. In general, the ammonium electrolyte **N2** was found to be optimal for electro‐oxidative acetalization. However, some exceptions were noted, such as substrate **2 e** which requires phosphonium electrolyte **P2**.

**Table 2:** Substrate scope for the electro‐oxidative acetalization.^[a]^

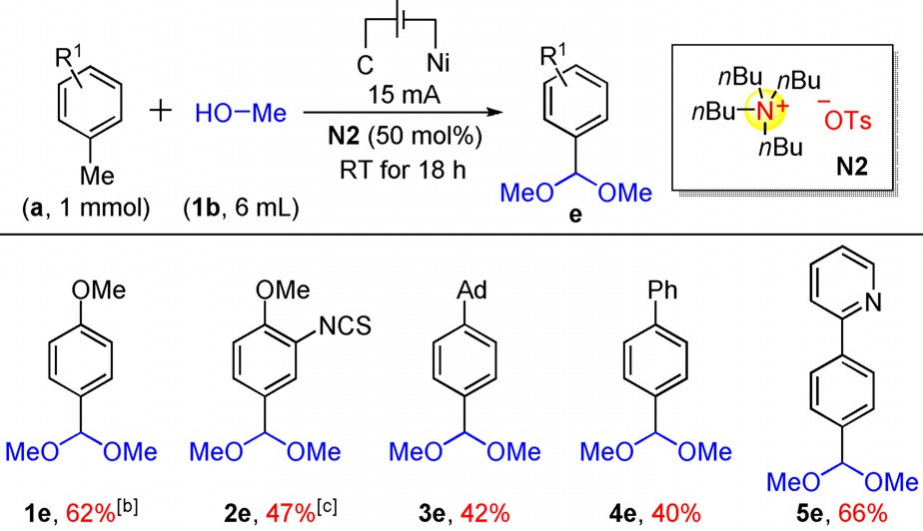

[a] Isolated yields. [b] The current was 5 mA. [c] Electrolyte **P2** was used instead of **N2**.

Next, we established that acetals and aldehydes are also competent substrates in this electro‐oxidative esterification reaction (Figure 3). The dehydrogenative esterification of benzaldehydes with alcohols was therefore investigated, as it represents a meaningful synthetic method. Indeed, in traditional methods,^[3]^ transition metals or/and strong chemical oxidants are usually required and the functional group tolerance typically limited. Remarkably, NHC catalysis has also been used for oxidative esterifications of aldehydes, however with an organic terminal oxidant.^[17]^ Under a low 5 mA current in combination with electrolyte **P2**, a variety of aldehydes could be dehydrogenatively coupled with alcohols to furnish the corresponding esters in good yields (Table 3). This new electro‐oxidative reaction moreover shows a very good tolerance for many oxidation sensitive functional groups (**16 c**, **34 c**, **35 c**, **36 c**, **37 c**), including olefins (**37 c**, **38 c**) and alkynes (**39 c**), making it a useful synthetic method.

**Figure 3:**
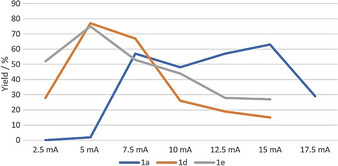
Esterification yields, depending on starting materials and currents. Yields determined by ^1^H NMR analysis of the crude reaction mixture with 1,3,5‐trimethoxybenzene as an internal standard.

**Table 3:** Substrate scope for the electro‐oxidative esterification of benzaldehydes.^[a]^

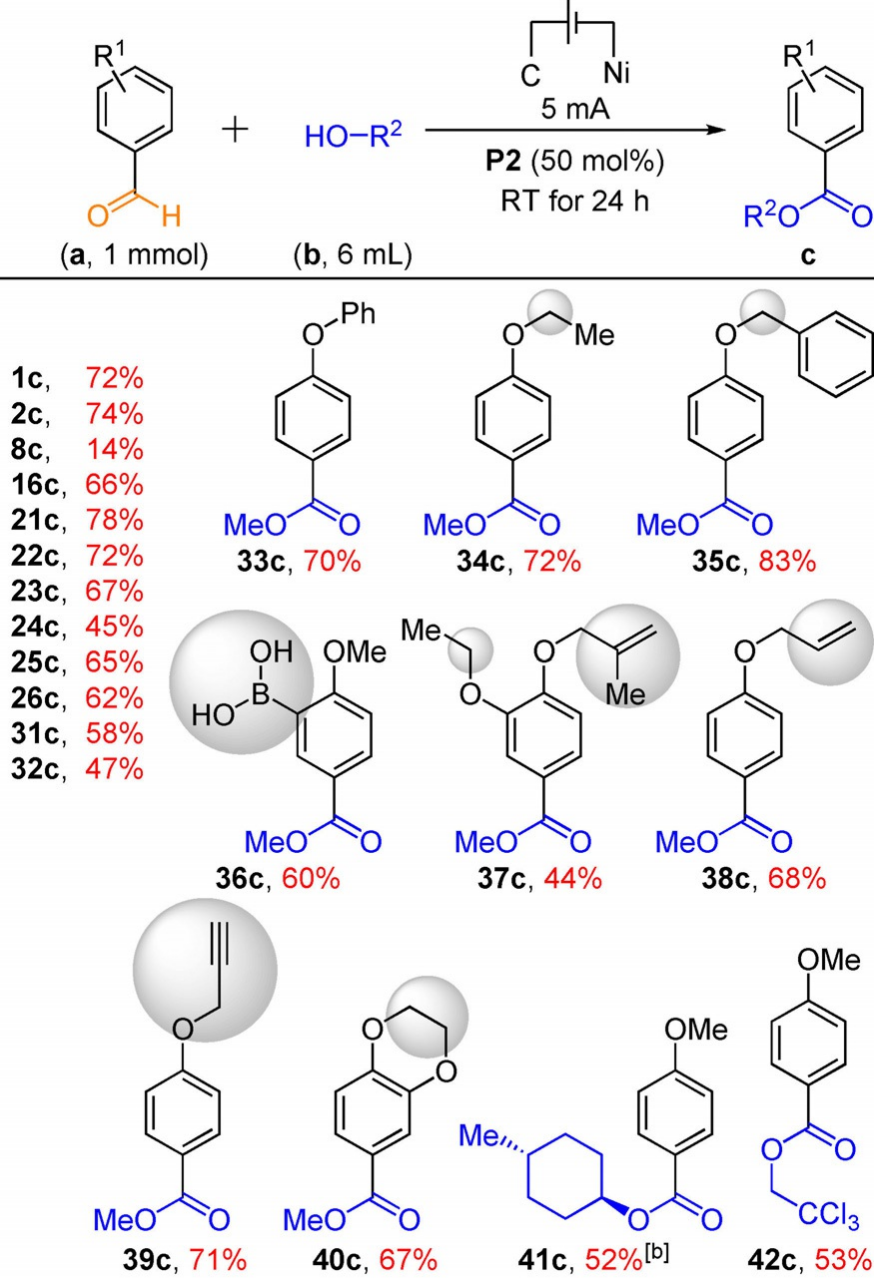

[a] Isolated yields. [b] 1.0 equivalent electrolyte was applied.

Some further selected mechanistic experiments were then performed. Firstly, when electricity was replaced by chemical oxidants such as *tert*‐butyl peroxide, sodium metaperiodate or potassium permanganate, under otherwise identical reaction conditions, none of the three expected oxidation products (**1 c**–**e**) could be observed. However, when CAN (ceric ammonium nitrate), or DDQ were used instead of electricity, some oxidation occurred forming the corresponding aldehyde **1 d** in 23 % and 8 % NMR yield, respectively (Scheme 2 a). This indicates that an aryl radical cation is most likely formed as a first reaction intermediate, in line with Yoshida's „radical‐cation pool“ concept.^[18]^ This also explains the selective reaction at the *para* position to the electron‐donating groups as well as the tolerance of some „fragile functional groups“ located at different positions. Moreover, control aldehyde **1 d’**—without an electron donating *para*‐methoxy functional group—could not be converted, thus highlighting the importance of the electron donating group (Scheme 2 b). Based on these elements, a mechanistic scenario is proposed in Scheme 3.

**Scheme 2:**
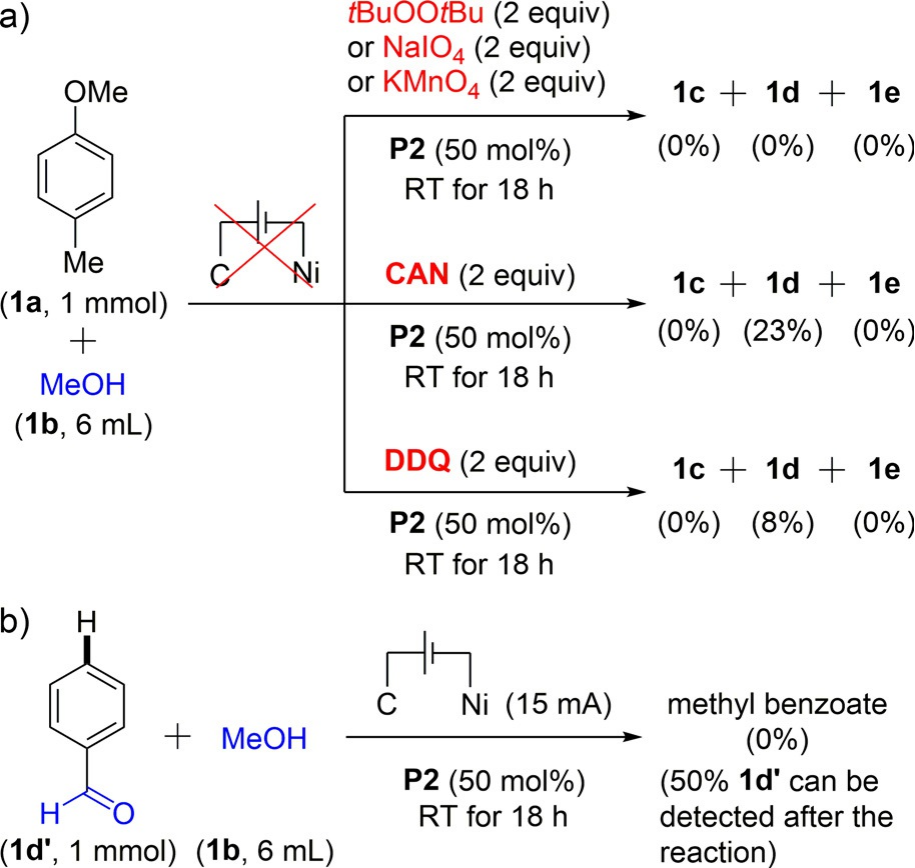
Selected mechanistic experiments.

**Scheme 3:**
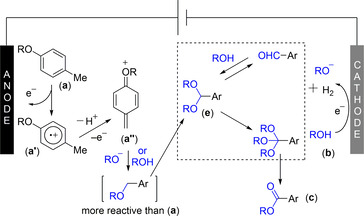
Mechanism proposal.

The aryl radical cation **a’** can be formed initially at the anode, followed by deprotonation and oxidation to form quinone methide derivative **a’’**. The latter would then be trapped by the alkoxide formed at the cathode, or simply by the high concentration excess alcohol. Further anodic oxidation events would deliver acetal intermediate **e**, followed by the oxidative formation of the corresponding orthoester, which in turn would hydrolyze either in situ or during work‐up to ester product **c**. The fact that the orthoester was typically not found may suggest the former scenario to be correct. Alternatively, the direct oxidation of acetal **e** at the anode toward ester product **c** (without the intermediacy of an orthoester) might also occur.

In summary, we have developed a mild and green electro‐oxidative method to construct esters and acetals from methylarenes and benzaldehydes. This electro‐oxidative method shows special tolerance to many easily oxidized structures such as hydroxys, aldehydes, olefins, alkynes, as well as other benzylic positions making it valuable in synthetic chemistry.

## Conflict of interest

The authors declare no conflict of interest.

## Supporting information

As a service to our authors and readers, this journal provides supporting information supplied by the authors. Such materials are peer reviewed and may be re‐organized for online delivery, but are not copy‐edited or typeset. Technical support issues arising from supporting information (other than missing files) should be addressed to the authors.

Supplementary
