# Supplementary material for: Electro‐Oxidative Selective Esterification of Methylarenes and Benzaldehydes
Source: Chemistry. 2021 Jan 25;27(11):3682–7. doi: 10.1002/chem.202005158 (PMC7986861; doi:10.1002/chem.202005158)
Supplement: Supplementary file 1 — Supplementary [file CHEM-27-3682-s001.pdf]

Chemistry–A European Journal

Supporting Information

Electro-Oxidative Selective Esterification of Methylarenes and Benzaldehydes

Congjun Yu, Bünyamin Özkaya, and Frederic W. Patureau*[a]

Table of Contents

1. General information…….S3
2. Condition optimization…….S3
3. Preparation of some starting materials…….S7
4. The equipment for the electro-oxidative reactions…….S10
5. Preparation of the esters and acetals from methylarenes…….S10
6. Preparation of the esters from aldehydes…….S26
7. References…….S31
8. Copies of ^{1}H and ^{13}C spectra, HPLC spectra…….S32

1. General information

NMR spectra were obtained on an Agilent VNMRS 400 or a Bruker Av 600 using $CDCl_3$ as solvents. Chemical shifts are given in ppm and coupling constants (*J*) in Hz. ^1H spectra were calibrated in relation to the reference measurement of TMS (0.00 ppm). ^{13}C spectra were calibrated in relation to the deuterated solvent, namely $CDCl_3$ (77.16 ppm). The following abbreviations were used for ^1H NMR spectra to indicate the signal multiplicity: s (singlet), d (doublet), t (triplet), q (quartet) and m (multiplet) as well as combinations of them. Flash chromatography was performed on silica gel (60 M, 0.04-0.063 mm) by standard technique. All the chemicals used for synthesis were purchased from Sigma Aldrich, abcr, Alfa Aesar, TCI, Fisher, or chemPUR. All the electrodes were purchased from IKA. High resolution mass spectra (HRMS) were recorded on ThermoFisher Scientific LTQ Orbitrap XL spectrometer. IR spectra were measured on a PerkinElmer 100 FT-IR spectrometer with an UATR Diamond KRS-5 unit. Cyclic voltammetry was performed using a PGSTAT101 from Metrohm Autolab with platinum disk working electrode (diameter 1 mm), Ag/Ag^+ (0.01 M $AgNO_3$, 0.1 M NBu_4PF_6, MeCN) reference electrode and platinum wire counter electrode. All measurements were recorded under nitrogen atmosphere with anhydrous solvents. The scan rate was 100 mV/s. All given potentials are reported versus ferrocene as internal reference.

2. Condition optimization

(1) The yields of three products with different electrolytes under different currents (^1H NMR yields with 1,3,5-trimethoxybenzene as an internal standard).

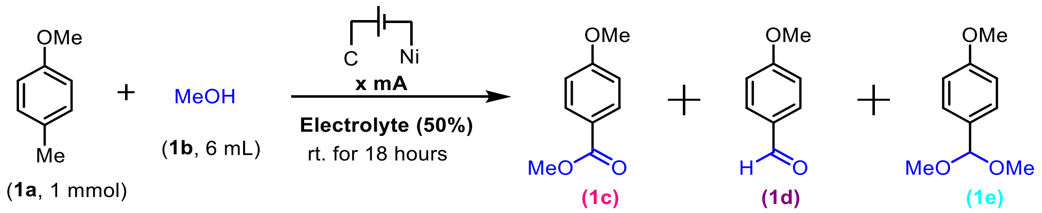

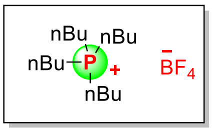

I (mA)	2.5 mA	5 mA	7.5 mA	10 mA	12.5 mA	15 mA	17.5 mA
1c (yield)	1%	2%	10%	40%	34%	57%	42%
1d (yield)	3%	8%	18%	45%	27%	0%	0%
1e (yield)	28%	44%	45%	0%	7%	0%	0%

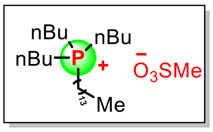

I (mA)	2.5 mA	5 mA	7.5 mA	10 mA	12.5 mA	15 mA	17.5 mA
1c (yield)	0%	2%	57%	48%	57%	63%	29%
1d (yield)	28%	50%	8%	24%	0%	0%	0%
1e (yield)	5%	7%	0%	10%	0%	0%	0%

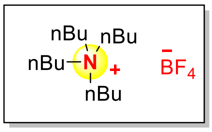

I (mA)	2.5 mA	5 mA	7.5 mA	10 mA	12.5 mA	15 mA	17.5 mA
1c (yield)	0%	1%	24%	8%	30%	22%	16%
1d (yield)	0%	6%	1%	10%	0%	12%	0%
1e (yield)	28%	50%	59%	56%	0%	36%	0%

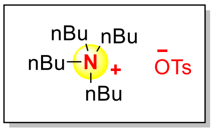

I (mA)	2.5 mA	5 mA	7.5 mA	10 mA	12.5 mA	15 mA	17.5 mA
1c (yield)	0%	0%	28%	57%	51%	34%	39%
1d (yield)	3%	2%	0%	0%	0%	0%	0
1e (yield)	26%	66%	45%	14%	0%	0%	0%

(2) The yields of three products with some different electrolytes (^{1}H NMR yields with 1,3,5-trimethoxybenzene as an internal standard).

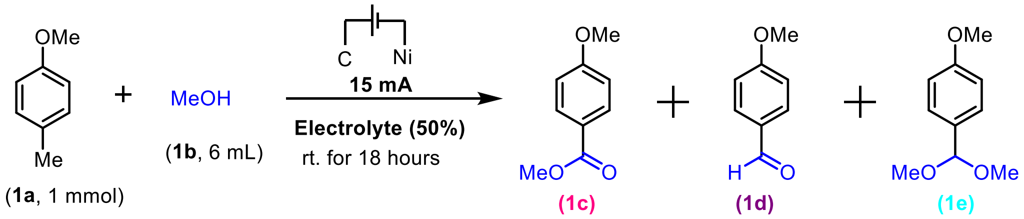

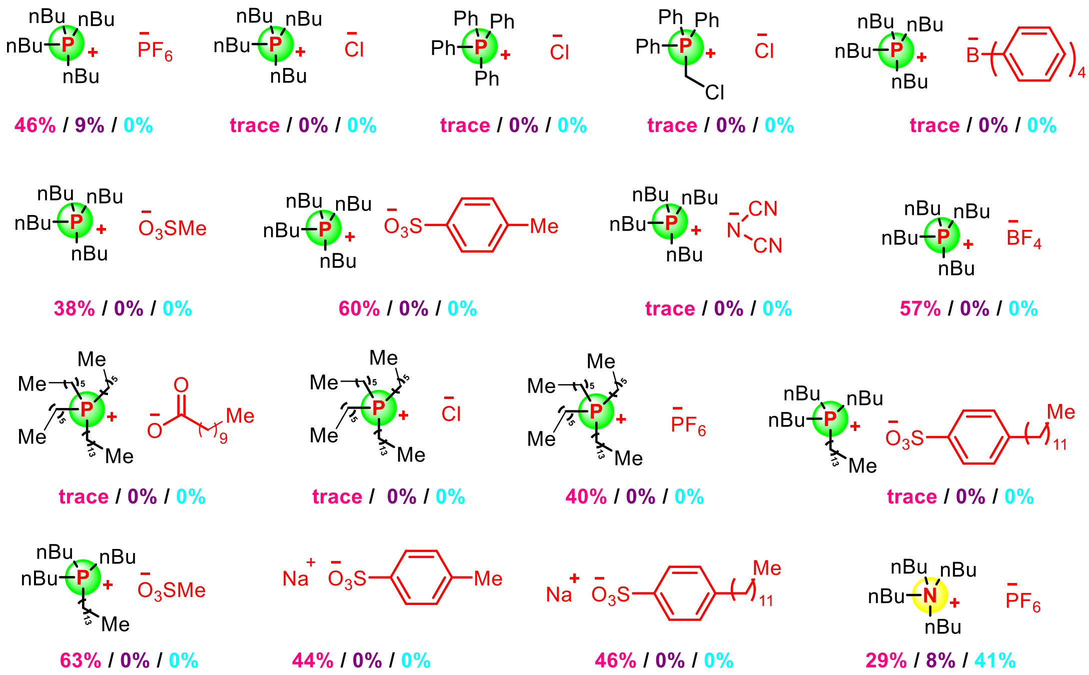

(3) The yields of three products with different amounts of electrolytes under different currents (^{1}H NMR yields with 1,3,5-trimethoxybenzene as an internal standard).

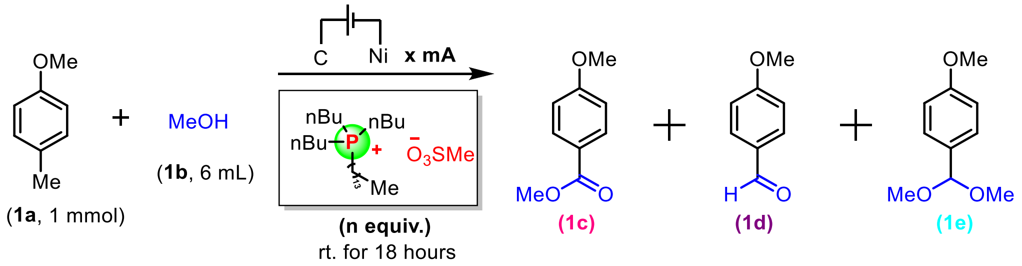

n x	5 mA	7.5 mA	10 mA	12.5 mA	15 mA
0.2 equiv.		30%/5%/36%	27%/0%/13%		
0.5 equiv.	1%/6%/50%	24%/1%/59%	8%/10%/56%	30%/0/0	22%/12%/36%
1.0 equiv.		13%/15%/47%	28%/10%/31%		
1.5 equiv.			27%/0%/19%		

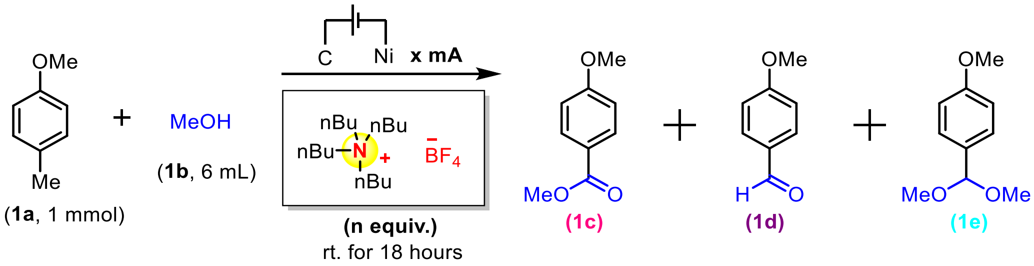

n x	5 mA	7.5 mA	10 mA	12.5 mA	15 mA	17.5 mA
0.2 equiv.			43%/24%/0%	45%/0/0	38%/0/0	23%/0/0
0.5 equiv.	2%/50%/7%	57%/8%/0	48%/24%/10%	57%/0/0	63%/0/0	29%/0/0
1.0 equiv.			65%/13%/0%	60%/0/0	52%/0/0	
1.5 equiv.				64%/3%/0	31%/0/0	

(4) The yields of acetalization of different substrates under different currents (^{1}H NMR yields with 1,3,5-trimethoxybenzene as an internal standard).

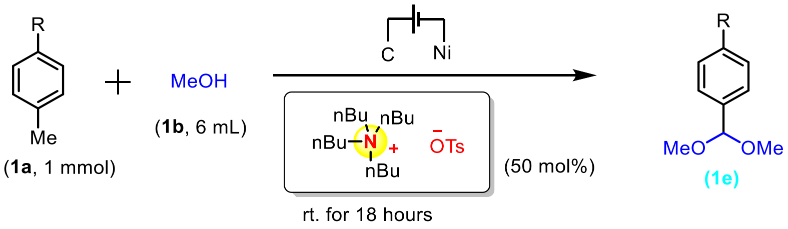

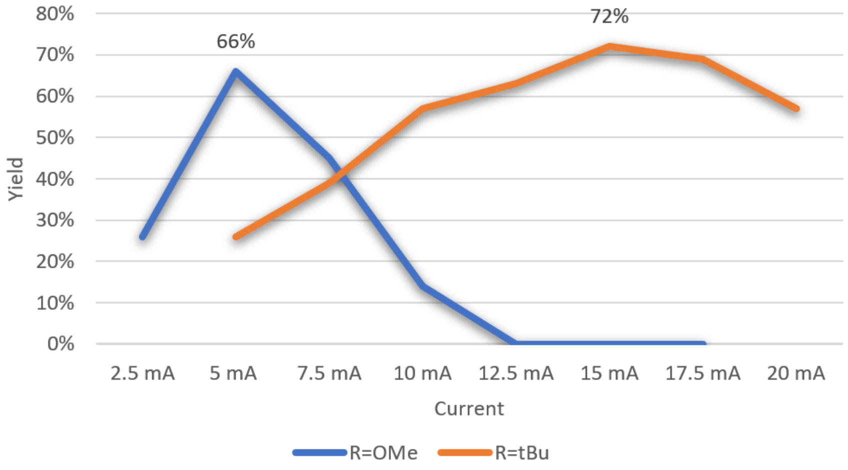

(5) Some other condition optimizations (^{1}H NMR yields with 1,3,5-trimethoxybenzene as an internal standard).

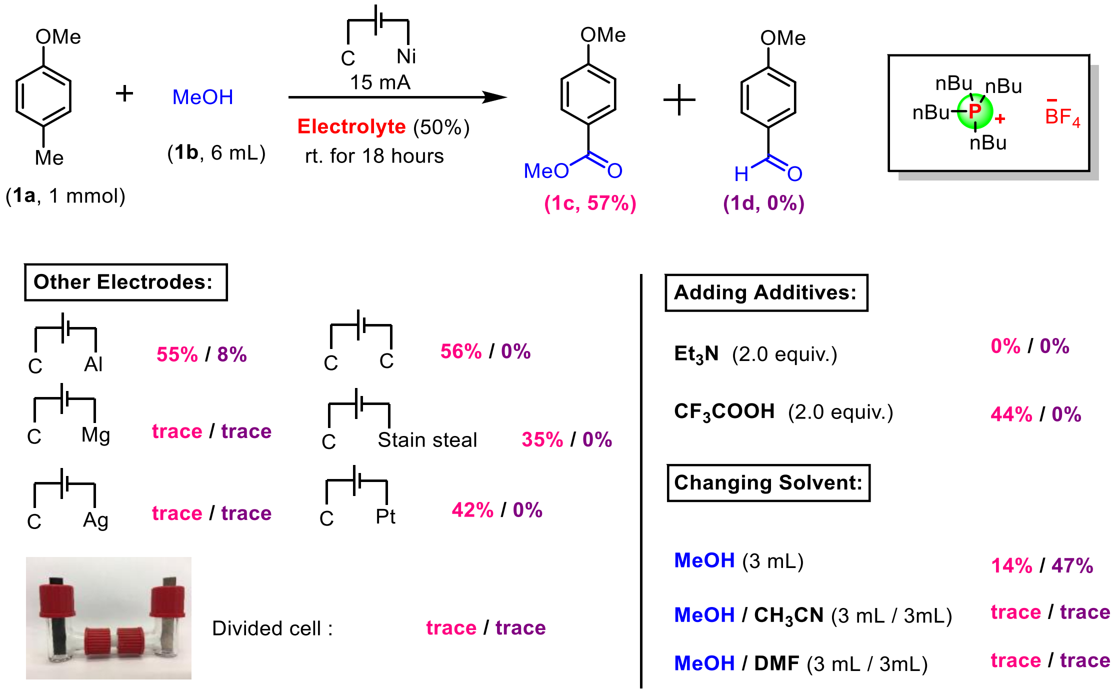

(6) Esterification yields, depending on starting materials and currents. Yields determined by ^{1}H NMR analysis of the crude reaction mixture with 1,3,5-trimethoxybenzene as an internal standard.

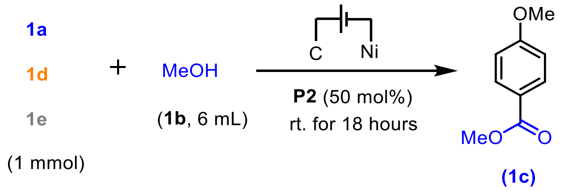

Yield	2.5 mA	5 mA	7.5 mA	10 mA	12.5 mA	15 mA	17.5 mA
1a	0%	2%	57%	48%	57%	63%	29%
1d	28%	77%	67%	26%	19%	15%	
1e	52%	75%	53%	44%	28%	27%	

3. Preparation of some starting materials

(1)

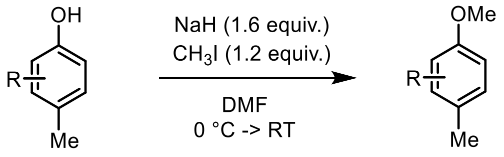

According to a known procedure,[S1] a schlenk-tube (100 mL) was charged with sodium hydride (60% powder in oil, 8 mmol, 320 mg) and DMF (20 mL). After the sodium hydride was dissolved, the corresponding phenol was added (5 mmol, X mg) while stirring and the mixture was cooled down to 0 °C. Methyl iodide (6 mmol, ≈ 400 µL) was added dropwise, the reaction was stirred (3h) and simultaneously allowed to acclimatize to room temperature. The reaction was quenched with water (20 mL), extracted with ethyl acetate (3x 10 mL), dried over $MgSO_4$ and concentrated under reduced pressure. The crude was purified by SiO_2 gel column chromatography.

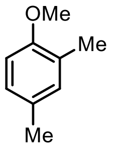

Chemical Formula: $C_9H_{12}O$

Following the procedure, the product **5a** was converted from 2,4-dimethylphenole (5 mmol, 611 mg). The crude mixture is purified by SiO_2 gel column chromatography with hexane/EA (95:5). Isolated yield: 544 mg, 80 % as a yellow oil.

^{1}H NMR (400 MHz, Chloroform-*d*) δ 6.95 – 6.82 (m, 2H), 6.64 (d, J = 8.8 Hz, 1H), 3.72 (s, 3H), 2.18 (s, 3H), 2.12 (s, 3H).

^{13}C NMR (101 MHz, Chloroform-*d*) δ 155.80, 131.63, 129.56, 127.05, 126.47, 110.09, 55.56, 20.54, 16.24.

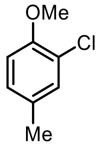

Chemical Formula: C_8H_9ClO

Following the procedure, the product **6a** was converted from 2-chloro-4-methylphenol (5 mmol, 713 mg). The crude mixture is purified by SiO_2 gel column chromatography with hexane. Isolated yield: 770 mg, 98 % as a white oil.

^{1}H NMR (400 MHz, Chloroform-*d*) δ 7.11 (d, *J* = 2.1 Hz, 1H), 6.94 (dd, *J* = 8.6, 2.2 Hz, 1H), 6.74 (d, *J* = 8.4 Hz, 1H), 3.80 (s, 3H), 2.20 (s, 3H).

^{13}C NMR (101 MHz, Chloroform-*d*) δ 151.97, 130.09, 129.86, 127.22, 121.10, 111.14, 55.33, 19.38.

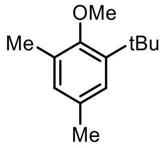

Chemical Formula: $C_{13}H_{20}O$

Following the procedure, the product **7a** was converted from 2,4-dimethyl-5-*tert*-butylphenol (5 mmol, 891 mg). The crude mixture is purified by SiO_2 gel column chromatography with hexane. Isolated yield: 845 mg, 88 % as a yellow oil.

^{1}H NMR (400 MHz, Chloroform-*d*) δ 6.88 (d, *J* = 2.2 Hz, 1H), 6.79 (d, *J* = 2.2 Hz, 1H), 3.67 (s, 3H), 2.21 (s, 3H), 2.19 (s, 3H), 1.30 (s, 9H).

^{13}C NMR (101 MHz, Chloroform-*d*) δ 156.29, 142.23, 132.33, 131.16, 130.49, 125.55, 60.74, 35.01, 31.18, 21.12, 17.35.

(2)

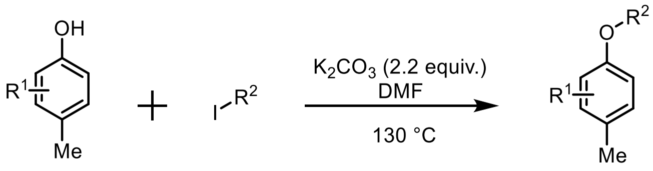

According to a modification of known procedure, a schlenk tube (100 mL) was charged with the phenol (1.1 equiv), the iodine compound (x mmol), potassium carbonate (2.2 equiv.) and DMF (0.7 M). The mixture was heated up at 130 °C (x h). The crude was washed with water (3x 10 mL) and ethyl acetate (3x 5 mL), dried over $MgSO_4$ and concentrated under reduced pressure. The crude was purified by SiO_2 gel column chromatography.

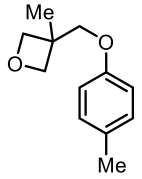

Chemical Formula: $C_{12}H_{16}O_2$

Following the procedure, the product **13a** was converted from *p*-cresol (2.1 mmol, 227 mg) and 3-(iodomethyl)-3-methyloxetane (2 mmol, 424 mg) and heated up for 3 h. The crude mixture is purified by SiO_2 gel column chromatography with pentane/EA/NEt_3 (8:1:1). Isolated yield: 267 mg, 69 % as a white oil.

^{1}H NMR (600 MHz, Chloroform-*d*) δ 7.02 (d, *J* = 8.2 Hz, 2H), 6.76 (d, *J* = 8.2 Hz, 2H), 4.55 (d, *J* = 6.3 Hz, 2H), 4.37 (d, *J* = 5.7 Hz, 2H), 3.92 (s, 2H), 2.22 (s, 3H), 1.36 (s, 3H).

^{13}C NMR (151 MHz, Chloroform-*d*) δ 157.11, 130.33, 130.05, 114.52, 80.02, 73.07, 39.85, 21.45, 20.59.

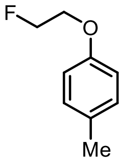

Chemical Formula: $C_9H_{11}FO$

Following the procedure, the product **17a** was converted from *p*-cresol (2.5 mmol, 270 mg) and 1-fluoro-2-iodoethane (2.4 mmol, 417 mg) and heated up overnight. The crude mixture is purified by SiO_2 gel column chromatography with pentane/EA (9:1). Isolated yield: 151 mg, 39 % as a white oil.

^{1}H NMR (400 MHz, Chloroform-*d*) δ 7.09 (d, *J* = 8.2 Hz, 2H), 6.83 (d, *J* = 8.1 Hz, 2H), 4.73 (dt, *J* = 47.6, 4.5 Hz, 2H), 4.17 (dt, *J* = 27.9, 4.2 Hz, 2H), 2.29 (s, 3H).

^{13}C NMR (101 MHz, Chloroform-*d*) δ 156.44, 130.67, 130.10, 114.65, 82.16 (d, $J_{C\text{-}F}$ = 170.1 Hz), 67.35 (d, $J_{C\text{-}F}$ = 20.6 Hz), 20.59.

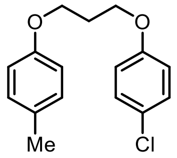

Chemical Formula: $C_{16}H_{17}ClO_2$

Following the procedure, the product **20a** was converted from *p*-cresol (2.5 mmol, 270 mg) and 1-chloro-4-(3-iodopropoxy)benzene (2.8 mmol, 827 mg) and heated up overnight. The crude mixture is purified by SiO_2 gel column chromatography with pentane/EA (9:1). Isolated yield: 277 mg, 40 % as a white powder.

^{1}H NMR (400 MHz, Chloroform-*d*) δ 7.26 – 7.17 (m, 2H), 7.06 (d, *J* = 8.2 Hz, 2H), 6.87 – 6.75 (m, 4H), 4.11 (t, *J* = 6.1 Hz, 4H), 2.27 (s, 3H), 2.26 – 2.19 (m, 2H).

^{13}C NMR (101 MHz, Chloroform-*d*) δ 156.65, 155.82, 129.16, 129.06, 128.44, 124.68, 114.92, 113.49, 63.98, 63.48, 28.43, 19.60.

4. The equipment for the electro-oxidative reactions

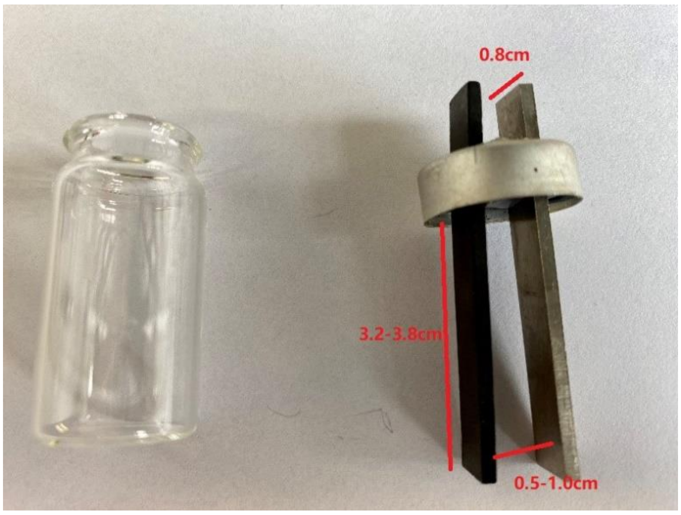

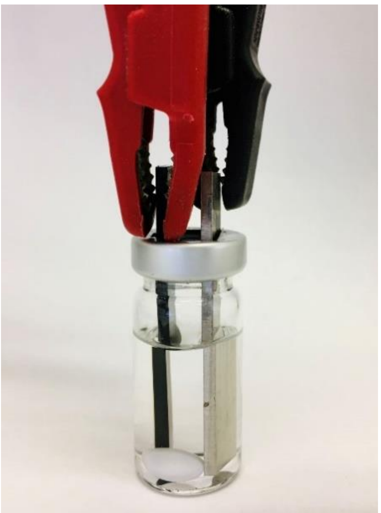

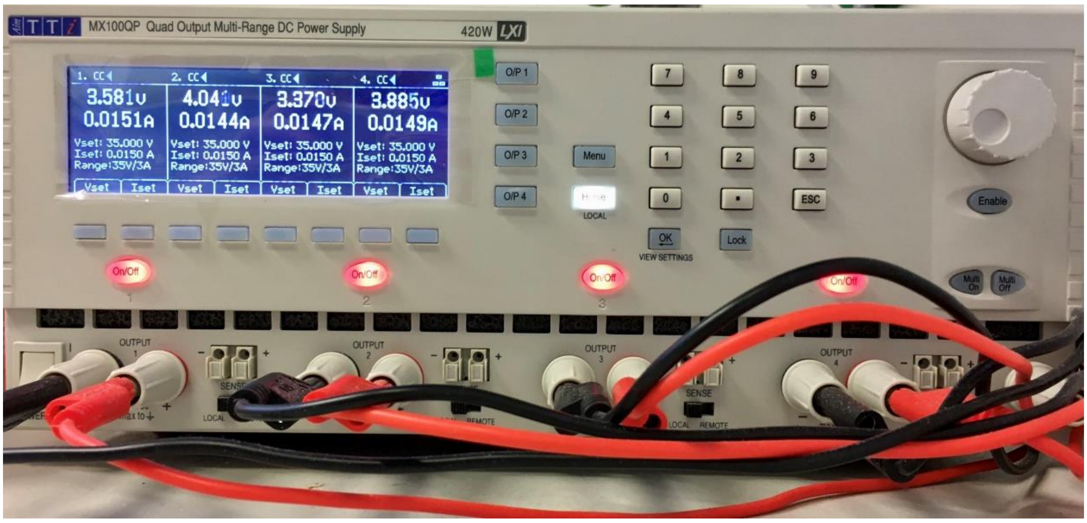

5. Preparation of the esters and acetals from methylarenes

General procedure for the esterification of methylarenes.

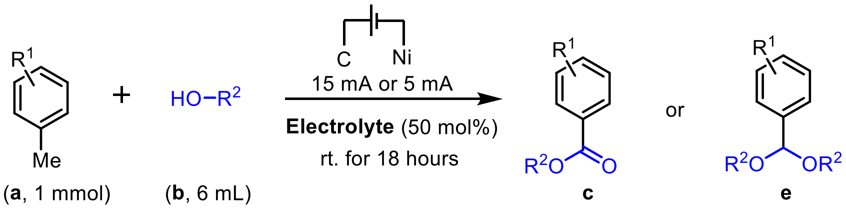

Unless otherwise specified, the methylarene **a** (1.0 mmol scale) and the electrolyte **P2** or **N1** or **N2** (0.5 mmol) are added into the alcohol **b** (6 mL) in a reaction vial. The reaction vial is then sealed

with aluminous headspace cap with electrodes (see the pictures above). The current is set to 15 mA or 5 mA. Keep the reaction stirring in room temperature for 18 hours. After that, the alcohol is removed and the crude is directly engaged on SiO_2 gel column chromatography for purification.

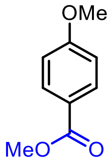

Chemical Formula: $C_9H_{10}O_3$

1c: Following the general procedure, 122 mg (1 mmol, 1 equiv.) starting material and 247 mg (50 mol%) electrolyte **P2** was used, the current was 15 mA. The crude mixture was purified by SiO_2 gel column chromatography with pentane/EA (from 40:1 to 20:1). 103 mg product was obtained by 62% isolated yield as white solid.

^{1}H NMR (600 MHz, Chloroform-*d*) δ 7.95 – 7.87 (m, 2H), 6.89 – 6.81 (m, 2H), 3.79 (s, 3H), 3.76 (s, 3H).

^{13}C NMR (151 MHz, Chloroform-*d*) δ 166.92, 163.43, 131.66, 122.70, 113.68, 55.46, 51.89.

ESI-HRMS: mass spectrometry: m/z calc. 189.05222 $[C_9H_{10}O_3Na]^+$, measured 189.05200.

IR (neat, cm^{-1}): ṽ: 3402, 3005, 2951, 2843, 1708, 1603, 1509, 1429, 1317, 1280, 1254, 1166, 1105, 1021, 962, 846, 768, 737, 696.

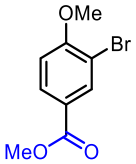

Chemical Formula: $C_9H_9BrO_3$

2c: Following the general procedure, 201 mg (1 mmol, 1 equiv.) starting material and 247 mg (50 mol%) electrolyte **P2** was used, the current was 15 mA. The crude mixture is purified by SiO_2 gel column chromatography with pentane/EA (20:1). 184 mg product was obtained by 75% isolated yield as grey solid.

^{1}H NMR (600 MHz, Chloroform-*d*) δ 8.16 (d, *J* = 2.5 Hz, 1H), 7.97 – 7.76 (m, 1H), 6.85 (d, *J* = 8.6 Hz, 1H), 3.89 (s, 3H), 3.82 (s, 3H).

^{13}C NMR (151 MHz, Chloroform-*d*) δ 165.86, 159.60, 134.95, 130.75, 123.90, 111.57, 111.15, 56.58, 52.30.

APCI-HRMS: mass spectrometry: m/z calc. 244.98078 $[C_9H_{10}O_3Br]^+$, measured 244.98133.

IR (neat, cm^{-1}): ṽ: 3076, 3008, 2952, 2842, 1703, 1597, 1496, 1465, 1426, 1393, 1313, 1264, 1189, 1152, 1115, 1050, 1012, 970, 906, 827, 761, 684.

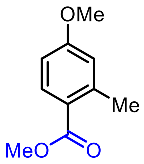

Chemical Formula: $C_{10}H_{12}O_3$

3c: Following the general procedure, 136 mg (1 mmol, 1 equiv.) starting material and 247 mg (50

mol%) electrolyte **P2** was used, the current was 15 mA. The crude mixture is purified by SiO_2 gel column chromatography with pentane/EA (from 40:1 to 20:1). 96 mg product was obtained by 53% isolated yield as colorless oil.

^{1}H NMR (600 MHz, Chloroform-*d*) δ 7.85 (d, *J* = 9.4 Hz, 1H), 6.66 (dd, *J* = 4.7, 2.3 Hz, 2H), 3.77 (s, 3H), 3.75 (s, 3H), 2.52 (s, 3H).

^{13}C NMR (151 MHz, Chloroform-*d*) δ 167.64, 162.41, 143.22, 133.07, 121.84, 117.02, 111.03, 55.36, 51.59, 22.42.

ESI-HRMS: mass spectrometry: m/z calc. 203.06787 $[C_{10}H_{12}O_3Na]^+$, measured 203.06764.

IR (neat, cm^{-1}): ṽ: 2954, 2870, 1715, 1602, 1493, 1436, 1360, 1302, 1239, 1185, 1148, 1119, 1095, 1025, 985, 916, 882, 825, 771, 742, 703.

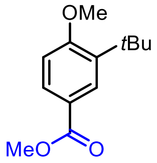

Chemical Formula: $C_{13}H_{18}O_3$

4c: Following the general procedure, 178 mg (1 mmol, 1 equiv.) starting material and 247 mg (50 mol%) electrolyte **P2** was used, the current was 15 mA. The crude mixture is purified by SiO_2 gel column chromatography with pentane/EA (from 40:1 to 20:1). 173 mg product was obtained by 78% isolated yield as colorless oil.

^{1}H NMR (600 MHz, Chloroform-*d*) δ 7.90 (d, *J* = 2.2 Hz, 1H), 7.81 (dd, *J* = 8.5, 2.2 Hz, 1H), 6.78 (d, *J* = 8.5 Hz, 1H), 3.84 – 3.71 (m, 6H), 1.30 (s, 9H).

^{13}C NMR (151 MHz, Chloroform-*d*) δ 167.40, 162.40, 138.17, 129.51, 128.38, 122.02, 110.88, 55.18, 51.82, 34.99, 29.63.

ESI-HRMS: mass spectrometry: m/z calc. 245.11482 $[C_{13}H_{18}O_3Na]^+$, measured 245.11457.

IR (neat, cm^{-1}): ṽ: 2954, 2870, 1715, 1602, 1493, 1436, 1360, 1302, 1239, 1185, 1148, 1119, 1095, 1025, 985, 916, 882, 825, 771, 742, 703.

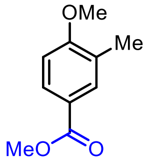

Chemical Formula: $C_{10}H_{12}O_3$

5c: Following the general procedure, 136 mg (1 mmol, 1 equiv.) starting material and 247 mg (50 mol%) electrolyte **P2** was used, the current was 15 mA. The crude mixture is purified by SiO_2 gel column chromatography with pentane/EA (from 40:1 to 20:1). 72 mg product was obtained by 40% isolated yield as white solid.

^{1}H NMR (600 MHz, Chloroform-*d*) δ 7.81 (dd, *J* = 8.5, 2.2 Hz, 1H), 7.75 (d, *J* = 2.2 Hz, 1H), 6.75 (d, *J* = 8.5 Hz, 1H), 3.80 (s, 6H), 2.16 (s, 3H).

^{13}C NMR (151 MHz, Chloroform-*d*) δ 167.26, 161.69, 132.09, 129.47, 126.72, 122.14, 109.34, 55.58, 51.91, 16.26.

ESI-HRMS: mass spectrometry: m/z calc. 203.06787 $[C_{10}H_{12}O_3Na]^+$, measured 203.06770.

IR (neat, cm^{-1}): ṽ: 3007, 2950, 2922, 2848, 1702, 1603, 1502, 1431, 1324, 1295, 1256, 1187, 1142, 1108, 1020, 912, 892, 827, 790, 763, 714.

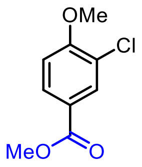

Chemical Formula: $C_9H_9ClO_3$

6c: Following the general procedure, 157 mg (1 mmol, 1 equiv.) starting material and 247 mg (50 mol%) electrolyte **P2** was used, the current was 15 mA. The crude mixture is purified by SiO_2 gel column chromatography with pentane/EA (20:1). 120 mg product was obtained by 60% isolated yield as white solid.

^{1}H NMR (600 MHz, Chloroform-*d*) δ 7.97 (d, *J* = 2.1 Hz, 1H), 7.85 (dd, *J* = 8.6, 2.1 Hz, 1H), 6.87 (d, *J* = 8.7 Hz, 1H), 3.88 (s, 3H), 3.81 (s, 3H).

^{13}C NMR (151 MHz, Chloroform-*d*) δ 165.95, 158.74, 131.76, 129.98, 123.45, 122.60, 111.32, 56.42, 52.23.

ESI-HRMS: mass spectrometry: m/z calc. 223.01324 $[C_9H_9O_3ClNa]^+$, measured 223.01299.

IR (neat, cm^{-1}): ṽ: 3019, 2955, 2848, 1700, 1598, 1573, 1502, 1428, 1274, 1240, 1186, 1164, 1121, 1059, 1015, 979, 910, 861, 827, 761, 707.

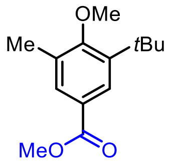

Chemical Formula: $C_{14}H_{20}O_3$

7c: Following the general procedure, 192 mg (1 mmol, 1 equiv.) starting material and 247 mg (50 mol%) electrolyte **P2** was used, the current was 15 mA. The crude mixture is purified by SiO_2 gel column chromatography with pentane/EA (from 30:1 to 10:1). 100 mg product was obtained by 42% isolated yield as yellow oil.

^{1}H NMR (600 MHz, Chloroform-*d*) δ 7.79 (d, *J* = 2.2 Hz, 1H), 7.67 (d, *J* = 2.2 Hz, 1H), 3.81 (s, 3H), 3.73 (s, 3H), 2.28 (s, 3H), 1.33 (s, 9H).

^{13}C NMR (151 MHz, Chloroform-*d*) δ 167.39, 162.80, 142.86, 131.66, 131.43, 126.70, 124.74, 60.69, 52.03, 35.23, 30.91, 17.62.

ESI-HRMS: mass spectrometry: m/z calc. 259.13047 $[C_{14}H_{20}O_3Na]^+$, measured 259.13029.

IR (neat, cm^{-1}): ṽ: 2954, 2871, 1718, 1599, 1471, 1436, 1414, 1360, 1308, 1224, 1151, 1007, 907, 769, 680.

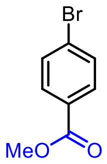

Chemical Formula: $C_8H_7BrO_2$

8c: Following the general procedure, 170 mg (1 mmol, 1 equiv.) starting material and 165 mg (50 mol%) electrolyte **N1** was used, the current was 15 mA. The crude mixture is purified by SiO_2 gel column chromatography with DCM. 110 mg product was obtained by 52% isolated yield as white solid.

^{1}H NMR (600 MHz, Chloroform-*d*) δ 7.92 (d, *J* = 8.2 Hz, 2H), 7.60 (d, *J* = 8.3 Hz, 2H), 3.94 (s, 3H).

^{13}C NMR (151 MHz, Chloroform-*d*) δ 166.52, 131.87, 131.27, 129.21, 128.19, 52.43.
APCI-HRMS: mass spectrometry: m/z calc. 214.97022 $[C_8H_8O_2{}^{79}Br]^+$, measured 214.97126.
IR (neat, cm^{-1}): ṽ: 2997, 2951, 2851, 1712, 1587, 1481, 1438, 1396, 1328, 1275, 1195, 1173, 1107, 1071, 1006, 956, 848, 824, 754, 685.

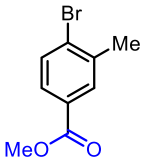

Chemical Formula: $C_9H_9BrO_2$

9c: Following the general procedure, 184 mg (1 mmol, 1 equiv.) starting material and 165 mg (50 mol%) electrolyte **N1** was used, the current was 15 mA. The crude mixture is purified by SiO_2 gel column chromatography with DCM. 120 mg product was obtained by 53% isolated yield as colorless oil.
^1H NMR (600 MHz, Chloroform-*d*) δ 8.22 (d, *J* = 1.7 Hz, 1H), 7.89 (dd, *J* = 7.9, 1.7 Hz, 1H), 7.32 (d, *J* = 7.9 Hz, 1H), 3.93 (s, 3H), 2.47 (s, 3H).
^{13}C NMR (151 MHz, Chloroform-*d*) δ 166.02, 143.47, 133.61, 130.85, 129.62, 128.51, 124.94, 52.39, 23.33.
APCI-HRMS: mass spectrometry: m/z calc. 228.98587 $[C_9H_{10}O_2{}^{79}Br]^+$, measured 228.98685.
IR (neat, cm^{-1}): ṽ: 3066, 2951, 2846, 1722, 1602, 1561, 1487, 1434, 1380, 1287, 1252, 1201, 1113, 1039, 972, 902, 843, 785, 756, 679.

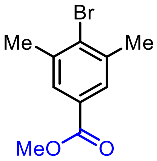

Chemical Formula: $C_{10}H_{11}BrO_2$

10c: Following the general procedure, 198 mg (1 mmol, 1 equiv.) starting material and 165 mg (50 mol%) electrolyte **N1** was used, the current was 15 mA. The crude mixture is purified by SiO_2 gel column chromatography with DCM. 120 mg product was obtained by 50% isolated yield as white solid.
^1H NMR (600 MHz, Chloroform-*d*) δ 7.75 (s, 2H), 3.92 (s, 3H), 2.47 (s, 6H).
^{13}C NMR (151 MHz, Chloroform-*d*) δ 166.94, 138.81, 133.18, 129.08, 128.53, 52.29, 23.99.
ESI-HRMS: mass spectrometry: m/z calc. 264.98346 $[C_{10}H_{11}O_2BrNa]^+$, measured 264.98377.
IR (neat, cm^{-1}): ṽ: 2956, 2925, 2853, 1761, 1715, 1592, 1441, 1381, 1315, 1225, 1119, 1013, 894, 762, 692.

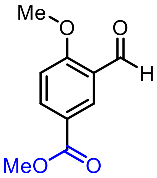

Chemical Formula: $C_{10}H_{10}O_4$

11c: Following the general procedure, 150 mg (1 mmol, 1 equiv.) starting material and 495 mg (1.0

equiv.) electrolyte **P2** was used, the current was 15 mA. The crude mixture is purified by SiO_2 gel column chromatography with pentane/EA (from 8:1 to 4:1). 78 mg product was obtained by 40% isolated yield as yellow solid.

^{1}H NMR (600 MHz, Chloroform-*d*) δ 10.46 (s, 1H), 8.51 (d, *J* = 2.3 Hz, 1H), 8.25 (dd, *J* = 8.8, 2.3 Hz, 1H), 7.06 (d, *J* = 8.8 Hz, 1H), 4.02 (s, 3H), 3.92 (s, 3H).

^{13}C NMR (151 MHz, Chloroform-*d*) δ 189.01, 166.12, 164.87, 137.25, 130.81, 124.60, 123.08, 111.70, 56.21, 52.26.

ESI-HRMS: mass spectrometry: m/z calc. 217.04713 $[C_{10}H_{10}O_4Na]^+$, measured 217.04666.

IR (neat, cm^{-1}): ṽ: 2952, 2872, 1715, 1678, 1602, 1493, 1435, 1300, 1260, 1182, 1120, 1011, 979, 920, 837, 760, 718.

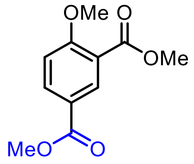

Chemical Formula: $C_{11}H_{12}O_5$

12c: Following the general procedure, 180 mg (1 mmol, 1 equiv.) starting material and 247 mg (50 mol%) electrolyte **P2** was used, the current was 15 mA. The crude mixture is purified by SiO_2 gel column chromatography with pentane/EA (from 8:1 to 4:1). 160 mg product was obtained by 71% isolated yield as white solid.

^{1}H NMR (600 MHz, Chloroform-*d*) δ 8.45 (d, *J* = 2.3 Hz, 1H), 8.13 (dd, *J* = 8.8, 2.3 Hz, 1H), 6.99 (d, *J* = 8.8 Hz, 1H), 3.95 (s, 3H), 3.91 – 3.86 (m, 6H).

^{13}C NMR (151 MHz, Chloroform-*d*) δ 166.05, 165.75, 162.54, 135.06, 133.53, 122.19, 119.99, 111.67, 56.30, 52.20, 52.10.

ESI-HRMS: mass spectrometry: m/z calc. 247.05769 $[C_{11}H_{12}O_5Na]^+$, measured 247.05729.

IR (neat, cm^{-1}): ṽ: 2956, 2845, 1705, 1605, 1502, 1435, 1310, 1272, 1226, 1187, 1120, 1077, 1012, 986, 878, 828, 762, 704, 679.

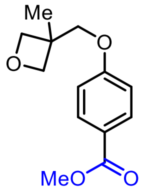

Chemical Formula: $C_{13}H_{16}O_4$

13c: Following the general procedure, 192 mg (1 mmol, 1 equiv.) starting material and 247 mg (50 mol%) electrolyte **P2** was used, the current was 15 mA. The crude mixture is purified by SiO_2 gel column chromatography with pentane/EA (from 10:1 to 5:1). 155 mg product was obtained by 66% isolated yield as yellow solid.

^{1}H NMR (600 MHz, Chloroform-*d*) δ 8.02 (d, *J* = 8.9 Hz, 2H), 6.97 (d, *J* = 8.9 Hz, 2H), 4.63 (d, *J* = 6.0 Hz, 2H), 4.48 (d, *J* = 6.0 Hz, 2H), 4.09 (s, 2H), 3.90 (s, 3H), 1.46 (s, 3H).

^{13}C NMR (151 MHz, Chloroform-*d*) δ 166.87, 162.83, 131.73, 123.04, 114.22, 79.75, 72.97, 51.97, 39.72, 21.31.

ESI-HRMS: mass spectrometry: m/z calc. 259.09408 $[C_{13}H_{16}O_4Na]^+$, measured 259.09382.

IR (neat, cm^{-1}): ṽ: 3065, 2956, 2878, 1701, 1600, 1510, 1456, 1427, 1392, 1316, 1246, 1164, 1106, 1035, 978, 944, 852, 834, 770, 732, 693.

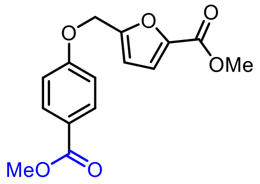

Chemical Formula: $C_{15}H_{14}O_6$

14c: Following the general procedure, 246 mg (1 mmol, 1 equiv.) starting material and 247 mg (50 mol%) electrolyte **P2** was used, the current was 15 mA. The crude mixture is purified by SiO_2 gel column chromatography with pentane/EA (from 10:1 to 5:1). 93 mg product was obtained by 32% isolated yield as white solid.

^{1}H NMR (400 MHz, Chloroform-*d*) δ 7.98 (d, *J* = 8.7 Hz, 2H), 7.14 (d, *J* = 3.5 Hz, 1H), 6.95 (d, *J* = 9.0 Hz, 2H), 6.52 (d, *J* = 3.5 Hz, 1H), 5.09 (s, 2H), 3.88 (s, 3H), 3.86 (s, 3H).

^{13}C NMR (101 MHz, Chloroform-*d*) δ 166.80, 161.73, 159.07, 153.81, 144.86, 131.79, 123.58, 118.87, 114.44, 111.79, 62.43, 52.19, 52.07.

ESI-HRMS: mass spectrometry: m/z calc. 313.06826 $[C_{15}H_{14}O_6Na]^+$, measured 313.06894.

IR (neat, cm^{-1}): ṽ: 2955, 1715, 1603, 1529, 1436, 1375, 1280, 1241, 1169, 1138, 1106, 1032, 995, 947, 854, 814, 761, 698, 625.

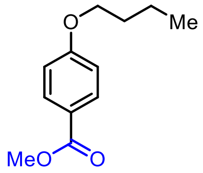

Chemical Formula: $C_{12}H_{16}O_3$

15c: Following the general procedure, 164 mg (1 mmol, 1 equiv.) starting material and 247 mg (50 mol%) electrolyte **P2** was used, the current was 15 mA. The crude mixture is purified by SiO_2 gel column chromatography with pentane/EA (from 40:1 to 20:1). 135 mg product was obtained by 65% isolated yield as colorless oil.

^{1}H NMR (600 MHz, Chloroform-*d*) δ 7.89 (d, *J* = 8.8 Hz, 2H), 6.81 (d, *J* = 8.8 Hz, 2H), 3.92 (t, *J* = 6.5 Hz, 2H), 3.79 (s, 3H), 1.78 – 1.62 (m, 2H), 1.51 – 1.36 (m, 2H), 0.90 (t, *J* = 7.4 Hz, 3H).

^{13}C NMR (151 MHz, Chloroform-*d*) δ 167.01, 163.08, 131.66, 122.44, 114.17, 67.98, 51.88, 31.26, 19.29, 13.90.

ESI-HRMS: mass spectrometry: m/z calc. 231.09917 $[C_{12}H_{16}O_3Na]^+$, measured 231.09896.

IR (neat, cm^{-1}): ṽ: 2954, 2873, 1715, 1604, 1510, 1465, 1434, 1389, 1312, 1251, 1167, 1106, 1069, 1009, 971, 847, 769, 695.

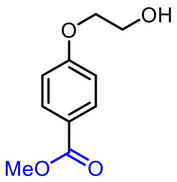

Chemical Formula: $C_{10}H_{12}O_4$

16c: Following the general procedure, 152 mg (1 mmol, 1 equiv.) starting material and 247 mg (50 mol%) electrolyte **P2** was used, the current was 15 mA. The crude mixture is purified by SiO_2 gel column chromatography with pentane/EA (from 5:1 to 2:1). 100 mg product was obtained by 51% isolated yield as yellow solid.

^{1}H NMR (600 MHz, Chloroform-*d*) δ 7.90 (d, *J* = 8.7 Hz, 2H), 6.84 (d, *J* = 8.7 Hz, 2H), 4.10 – 4.01 (m, 2H), 3.96 – 3.88 (m, 2H), 3.80 (s, 3H), 2.32 (s, 1H).

^{13}C NMR (151 MHz, Chloroform-*d*) δ 166.94, 162.51, 131.73, 123.04, 114.23, 69.47, 61.31, 51.99.

ESI-HRMS: mass spectrometry: m/z calc. 219.06278 $[C_{10}H_{12}O_4Na]^+$, measured 219.06247.

IR (neat, cm^{-1}): ṽ: 3293 (broad), 2949, 2878, 1717, 1604, 1508, 1431, 1379, 1314, 1278, 1250, 1190, 1167, 1089, 1049, 1012, 953, 920, 894, 847, 766, 697.

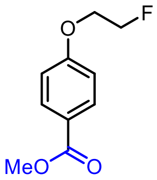

Chemical Formula: $C_{10}H_{11}FO_3$

17c: Following the general procedure, 154 mg (1 mmol, 1 equiv.) starting material and 247 mg (50 mol%) electrolyte **P2** was used, the current was 15 mA. The crude mixture is purified by SiO_2 gel column chromatography with pentane/EA (from 40:1 to 20:1). 140 mg product was obtained by 71% isolated yield as white solid.

^{1}H NMR (600 MHz, Chloroform-*d*) δ 8.02 (d, *J* = 8.5 Hz, 2H), 6.96 (d, *J* = 8.5 Hz, 2H), 4.90 – 4.67 (m, 2H), 4.36 – 4.21 (m, 2H), 3.90 (s, 3H).

^{13}C NMR (151 MHz, Chloroform-*d*) δ 166.85, 162.23, 131.77, 123.33, 114.29, 81.76 (d, J_{C-F} = 171.2 Hz), 67.28 (d, J_{C-F} = 20.6 Hz), 52.01.

ESI-HRMS: mass spectrometry: m/z calc. 221.05844 $[C_{10}H_{11}O_3FNa]^+$, measured 221.05839.

IR (neat, cm^{-1}): ṽ: 2999, 2951, 2924, 2852, 1705, 1603, 1510, 1435, 1372, 1313, 1280, 1251, 1196, 1165, 1107, 1069, 1037, 962, 917, 876, 846, 765, 693.

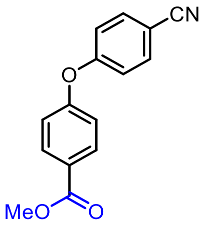

Chemical Formula: $C_{15}H_{11}NO_3$

18c: Following the general procedure, 209 mg (1 mmol, 1 equiv.) starting material and 247 mg (50 mol%) electrolyte **P2** was used, the current was 15 mA. The crude mixture is purified by SiO_2 gel

column chromatography with DCM. 110 mg product was obtained by 43% isolated yield as white solid.

^{1}H NMR (600 MHz, Chloroform-*d*) δ 8.09 (d, *J* = 8.5 Hz, 2H), 7.67 (d, *J* = 8.7 Hz, 2H), 7.17 – 7.03 (m, 4H), 3.94 (s, 3H).

^{13}C NMR (151 MHz, Chloroform-*d*) δ 166.31, 160.27, 159.32, 134.44, 132.11, 126.62, 119.28, 119.26, 118.60, 107.29, 52.30.

ESI-HRMS: mass spectrometry: m/z calc. 276.06284 $[C_{15}H_{11}O_3NNa]^+$, measured 276.06370.

IR (neat, cm^{-1}): ṽ: 2998, 2950, 2230, 1708, 1592, 1496, 1431, 1319, 1279, 1238, 1170, 1105, 1013, 962, 862, 760, 688.

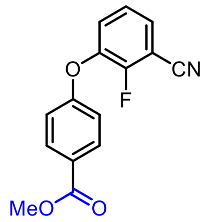

Chemical Formula: $C_{15}H_{10}FNO_3$

19c: Following the general procedure, 227 mg (1 mmol, 1 equiv.) starting material and 247 mg (50 mol%) electrolyte **P2** was used, the current was 15 mA. The crude mixture is purified by SiO_2 gel column chromatography with DCM. 130 mg product was obtained by 48% isolated yield as white solid.

^{1}H NMR (600 MHz, Chloroform-*d*) δ 8.12 (d, *J* = 8.2 Hz, 2H), 7.59 – 7.46 (m, 1H), 7.15 (d, *J* = 8.3 Hz, 2H), 7.00 (t, *J* = 8.2 Hz, 1H), 6.75 (d, *J* = 8.6 Hz, 1H), 3.95 (s, 3H).

^{13}C NMR (151 MHz, Chloroform-*d*) δ 166.24, 164.24 (d, $J_{C\text{-}F}$ = 260.6 Hz), 159.68 (d, $J_{C\text{-}F}$ = 3.6 Hz), 158.74, 135.19 (d, $J_{C\text{-}F}$ = 10.5 Hz), 132.18, 127.30, 119.44, 113.39 (d, $J_{C\text{-}F}$ = 3.5 Hz), 110.99 (d, $J_{C\text{-}F}$ = 19.4 Hz), 110.85, 94.90 (d, $J_{C\text{-}F}$ = 18.1 Hz), 52.38.

ESI-HRMS: mass spectrometry: m/z calc. 294.05306 $[C_{15}H_{10}O_3NFNa]^+$, measured 294.05411.

IR (neat, cm^{-1}): ṽ: 3105, 3072, 2961, 2924, 2854, 2236, 1713, 1610, 1583, 1503, 1459, 1288, 1245, 1158, 1110, 1015, 955, 865, 813, 780, 710, 680.

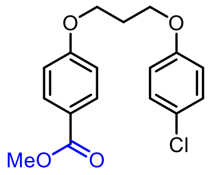

Chemical Formula: $C_{17}H_{17}ClO_4$

20c: Following the general procedure, 276 mg (1 mmol, 1 equiv.) starting material and 247 mg (50 mol%) electrolyte **P2** was used, the current was 15 mA. The crude mixture is purified by SiO_2 gel column chromatography with pentane/EA (from 20:1 to 10:1). 160 mg product was obtained by 50% isolated yield as white solid.

^{1}H NMR (600 MHz, Chloroform-*d*) δ 8.01 (d, *J* = 8.5 Hz, 2H), 7.25 (d, *J* = 8.6 Hz, 2H), 6.94 (d, *J* = 8.6 Hz, 2H), 6.86 (d, *J* = 8.5 Hz, 2H), 4.23 (t, *J* = 6.1 Hz, 2H), 4.15 (t, *J* = 6.0 Hz, 2H), 3.91 (s, 3H), 2.36 – 2.23 (m, 2H).

^{13}C NMR (151 MHz, Chloroform-*d*) δ 166.96, 162.72, 157.53, 131.75, 129.48, 125.85, 122.86, 115.89, 114.21, 64.67, 64.63, 51.99, 29.25.

ESI-HRMS: mass spectrometry: m/z calc. 343.07076 $[C_{17}H_{17}O_4ClNa]^+$, measured 343.07053.

IR (neat, cm^{-1}): ṽ: 2929, 2879, 1716, 1603, 1490, 1462, 1433, 1378, 1315, 1285, 1237, 1195, 1162, 1108, 1061, 994, 970, 827, 766, 694, 670.

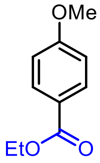

Chemical Formula: $C_{10}H_{12}O_3$

21c: Following the general procedure, 122 mg (1 mmol, 1 equiv.) starting material and 247 mg (50 mol%) electrolyte **P2** was used, the current was 15 mA. The crude mixture is purified by SiO_2 gel column chromatography with pentane/EA (30:1). 126 mg product was obtained by 70% isolated yield as colorless oil.

^1H NMR (600 MHz, Chloroform-*d*) δ 8.08 – 7.97 (m, 2H), 6.99 – 6.85 (m, 2H), 4.36 (q, *J* = 7.1 Hz, 2H), 3.86 (s, 3H), 1.39 (t, *J* = 7.1 Hz, 3H).

^{13}C NMR (151 MHz, Chloroform-*d*) δ 166.47, 163.36, 131.62, 123.07, 113.63, 60.70, 55.47, 14.46.

ESI-HRMS: mass spectrometry: m/z calc. 203.06787 $[C_{10}H_{12}O_3Na]^+$, measured 203.06755.

IR (neat, cm^{-1}): ṽ: 2979, 2841, 1708, 1605, 1510, 1461, 1421, 1367, 1314, 1253, 1166, 1102, 1027, 848, 769, 695.

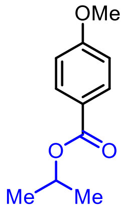

Chemical Formula: $C_{11}H_{14}O_3$

22c: Following the general procedure, 122 mg (1 mmol, 1 equiv.) starting material and 247 mg (50 mol%) electrolyte **P2** was used, the current was 15 mA. The crude mixture is purified by SiO_2 gel column chromatography with pentane/EA (from 40:1 to 20:1). 110 mg product was obtained by 57% isolated yield as yellow oil.

^1H NMR (600 MHz, Chloroform-*d*) δ 7.97 – 7.89 (m, 2H), 6.89 – 6.76 (m, 2H), 5.14 (hept, *J* = 6.3 Hz, 1H), 3.77 (s, 3H), 1.27 (d, *J* = 6.3 Hz, 6H).

^{13}C NMR (151 MHz, Chloroform-*d*) δ 165.99, 163.30, 131.61, 123.51, 113.60, 68.02, 55.49, 22.10.

ESI-HRMS: mass spectrometry: m/z calc. 217.08352 $[C_{11}H_{14}O_3Na]^+$, measured 217.08319.

IR (neat, cm^{-1}): ṽ: 2979, 2936, 2842, 1706, 1605, 1510, 1461, 1420, 1351, 1315, 1253, 1167, 1099, 1028, 920, 848, 771, 696.

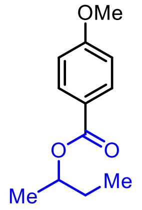

23c: Following the general procedure, 122 mg (1 mmol, 1 equiv.) starting material and 247 mg (50 mol%) electrolyte **P2** was used, the current was 15 mA. The crude mixture is purified by SiO_2 gel column chromatography with pentane/EA (from 40:1 to 20:1). 85 mg product was obtained by 41% isolated yield as colorless oil.

^{1}H NMR (600 MHz, Chloroform-*d*) δ 7.93 (d, *J* = 8.9 Hz, 2H), 6.84 (d, *J* = 8.8 Hz, 2H), 5.11 – 4.86 (m, 1H), 3.79 (s, 3H), 1.71 – 1.55 (m, 2H), 1.25 (d, *J* = 6.3 Hz, 3H), 0.89 (t, *J* = 7.5 Hz, 3H).

^{13}C NMR (151 MHz, Chloroform-*d*) δ 166.05, 163.20, 131.51, 123.43, 113.52, 72.45, 55.42, 29.01, 19.62, 9.75.

APCI-HRMS: mass spectrometry: m/z calc. 209.11722 $[C_{12}H_{17}O_3]^+$, measured 209.11818.

IR (neat, cm^{-1}): ṽ: 2970, 2935, 2879, 1707, 1605, 1509, 1459, 1378, 1354, 1312, 1253, 1167, 1098, 1029, 890, 848, 770, 696.

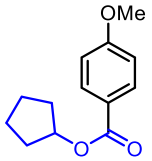

24c: Following the general procedure, 122 mg (1 mmol, 1 equiv.) starting material and 247 mg (50 mol%) electrolyte **P2** was used, the current was 15 mA. The crude mixture is purified by SiO_2 gel column chromatography with pentane/EA (from 40:1 to 20:1). 100 mg product was obtained by 45% isolated yield as yellow oil.

^{1}H NMR (600 MHz, Chloroform-*d*) δ 8.04 – 7.95 (m, 2H), 6.97 – 6.82 (m, 2H), 5.40 (tt, *J* = 6.3, 2.8 Hz, 1H), 3.87 (s, 3H), 2.02 – 1.92 (m, 2H), 1.89 – 1.78 (m, 4H), 1.70 – 1.64 (m, 2H).

^{13}C NMR (151 MHz, Chloroform-*d*) δ 166.27, 163.30, 131.60, 123.52, 113.61, 55.52, 32.92, 23.95.

ESI-HRMS: mass spectrometry: m/z calc. 243.09917 $[C_{13}H_{16}O_3Na]^+$, measured 243.09878.

IR (neat, cm^{-1}): ṽ: 2959, 2872, 1706, 1605, 1510, 1458, 1421, 1362, 1317, 1253, 1163, 1106, 1029, 962, 899, 847, 770, 696.

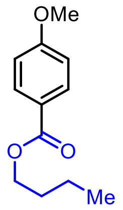

25c: Following the general procedure, 122 mg (1 mmol, 1 equiv.) starting material and 247 mg (50 mol%) electrolyte **P2** was used, the current was 15 mA. The crude mixture is purified by SiO_2 gel

column chromatography with pentane/EA (from 40:1 to 20:1). 80 mg product was obtained by 38% isolated yield as colorless oil.

^{1}H NMR (600 MHz, Chloroform-*d*) δ 7.93 (d, *J* = 8.8 Hz, 2H), 6.84 (d, *J* = 8.7 Hz, 2H), 4.22 (t, *J* = 6.6 Hz, 2H), 3.79 (s, 3H), 1.74 – 1.63 (m, 2H), 1.46 – 1.36 (m, 2H), 0.91 (t, *J* = 7.9, 6.9 Hz, 3H).

^{13}C NMR (151 MHz, Chloroform-*d*) δ 166.62, 163.40, 131.68, 123.16, 113.71, 64.68, 55.56, 19.44, 13.92.

ESI-HRMS: mass spectrometry: m/z calc. 209.11722 $[C_{12}H_{17}O_3]^+$, measured 209.11710.

IR (neat, cm^{-1}): ṽ: 2958, 2873, 1710, 1605, 1509, 1460, 1385, 1315, 1253, 1166, 1103, 1028, 947, 846, 813, 769, 695.

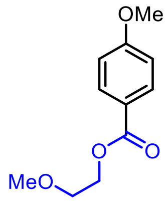

Chemical Formula: $C_{11}H_{14}O_4$

26c: Following the general procedure, 122 mg (1 mmol, 1 equiv.) starting material and 247 mg (50 mol%) electrolyte **P2** was used, the current was 15 mA. The crude mixture is purified by SiO_2 gel column chromatography with pentane/EA (from 40:1 to 20:1). 82 mg product was obtained by 39% isolated yield as colorless oil.

^{1}H NMR (600 MHz, Chloroform-*d*) δ 7.95 (d, *J* = 8.9 Hz, 2H), 6.84 (d, *J* = 8.8 Hz, 2H), 4.45 – 4.30 (m, 2H), 3.79 (s, 3H), 3.69 – 3.62 (m, 2H), 3.36 (s, 3H).

^{13}C NMR (151 MHz, Chloroform-*d*) δ 166.47, 163.56, 131.89, 122.66, 113.71, 70.82, 63.90, 59.19, 55.55.

ESI-HRMS: mass spectrometry: m/z calc. 233.07843 $[C_{11}H_{14}O_4Na]^+$, measured 233.07831.

IR (neat, cm^{-1}): ṽ: 2932, 2841, 1710, 1605, 1511, 1456, 1369, 1314, 1252, 1167, 1098, 1027, 845, 769, 695.

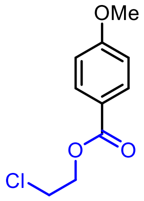

Chemical Formula: $C_{10}H_{11}ClO_3$

27c: Following the general procedure, 122 mg (1 mmol, 1 equiv.) starting material and 247 mg (50 mol%) electrolyte **P2** was used, the current was 15 mA. The crude mixture is purified by SiO_2 gel column chromatography with pentane/EA (from 40:1 to 20:1). 100 mg product was obtained by 47% isolated yield as grey solid.

^{1}H NMR (600 MHz, Chloroform-*d*) δ 7.95 (d, *J* = 8.5 Hz, 2H), 6.86 (d, *J* = 8.5 Hz, 2H), 4.47 (t, *J* = 5.8 Hz, 2H), 3.79 (s, 3H), 3.73 (t, *J* = 5.8 Hz, 2H).

^{13}C NMR (151 MHz, Chloroform-*d*) δ 166.05, 163.78, 131.96, 113.85, 64.32, 55.59, 41.93.

ESI-HRMS: mass spectrometry: m/z calc. 237.02889 $[C_{10}H_{11}O_3ClNa]^+$, measured 237.02875.

IR (neat, cm^{-1}): ṽ: 2960, 2843, 1713, 1604, 1510, 1456, 1381, 1308, 1253, 1167, 1104, 1026, 847, 767, 695, 666.

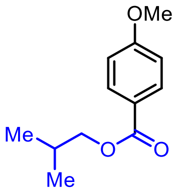

Chemical Formula: $C_{12}H_{16}O_3$

28c: Following the general procedure, 122 mg (1 mmol, 1 equiv.) starting material and 247 mg (50 mol%) electrolyte **P2** was used, the current was 15 mA. The crude mixture is purified by SiO_2 gel column chromatography with pentane/EA (from 40:1 to 20:1). 125 mg product was obtained by 60% isolated yield as yellow oil.

^{1}H NMR (600 MHz, Chloroform-*d*) δ 8.03 (d, *J* = 8.7 Hz, 2H), 6.94 (d, *J* = 8.6 Hz, 2H), 4.10 (d, *J* = 6.6 Hz, 2H), 3.88 (s, 3H), 2.20 – 2.01 (m, 1H), 1.04 (d, *J* = 6.7 Hz, 6H).

^{13}C NMR (151 MHz, Chloroform-*d*) δ 166.53, 163.41, 131.66, 123.13, 113.70, 70.84, 55.52, 28.06, 19.34.

ESI-HRMS: mass spectrometry: m/z calc. 231.09917 $[C_{12}H_{16}O_3Na]^+$, measured 231.09879.

IR (neat, cm^{-1}): ṽ: 2960, 2877, 1711, 1606, 1510, 1464, 1420, 1374, 1312, 1254, 1166, 1103, 1028, 984, 847, 769, 695.

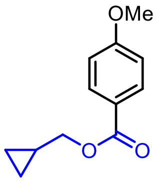

Chemical Formula: $C_{12}H_{14}O_3$

29c: Following the general procedure, 122 mg (1 mmol, 1 equiv.) starting material and 247 mg (50 mol%) electrolyte **P2** was used, the current was 15 mA. The crude mixture is purified by SiO_2 gel column chromatography with pentane/EA (from 40:1 to 20:1). 145 mg product was obtained by 70% isolated yield as colorless oil.

^{1}H NMR (600 MHz, Chloroform-*d*) δ 7.81 (d, *J* = 8.9 Hz, 2H), 6.71 (d, *J* = 8.9 Hz, 2H), 3.92 (s, 2H), 3.65 (s, 3H), 1.09 – 0.98 (m, 1H), 0.44 – 0.36 (m, 2H), 0.21 – 0.10 (m, 2H).

^{13}C NMR (151 MHz, Chloroform-*d*) δ 166.63, 163.40, 131.74, 123.14, 113.68, 69.47, 55.53, 10.09, 3.38.

ESI-HRMS: mass spectrometry: m/z calc. 229.08352 $[C_{12}H_{14}O_3Na]^+$, measured 229.08338.

IR (neat, cm^{-1}): ṽ: 3081, 3008, 2948, 2842, 1707, 1605, 1510, 1460, 1420, 1345, 1314, 1252, 1166, 1098, 1026, 964, 846, 769, 695.

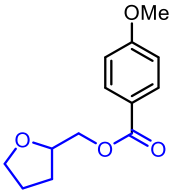

Chemical Formula: $C_{13}H_{16}O_4$

30c: Following the general procedure, 122 mg (1 mmol, 1 equiv.) starting material and 247 mg (50 mol%) electrolyte **P2** was used, the current was 15 mA. The crude mixture is purified by SiO_2 gel

column chromatography with pentane/EA (from 10:1) and second time with DCM. 150 mg product was obtained by 64% isolated yield as colorless oil.

^{1}H NMR (600 MHz, Chloroform-*d*) δ 7.94 (d, *J* = 8.9 Hz, 2H), 6.84 (d, *J* = 8.8 Hz, 2H), 4.34 – 4.24 (m, 1H), 4.23 – 4.13 (m, 2H), 3.91 – 3.83 (m, 1H), 3.78 (s, 3H), 3.78 – 3.72 (m, 1H), 2.05 – 1.95 (m, 1H), 1.95 – 1.80 (m, 2H), 1.69 – 1.60 (m, 1H).

^{13}C NMR (151 MHz, Chloroform-*d*) δ 166.43, 163.54, 131.87, 122.66, 113.71, 76.83, 68.66, 66.81, 55.54, 28.25, 25.89.

ESI-HRMS: mass spectrometry: m/z calc. 259.09408 $[C_{13}H_{16}O_4Na]^+$, measured 259.09381.

IR (neat, cm^{-1}): ṽ: 2953, 2872, 1710, 1605, 1510, 1456, 1384, 1314, 1254, 1167, 1082, 1024, 925, 848, 769, 696.

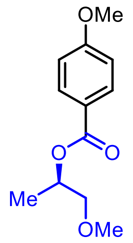

Chemical Formula: $C_{12}H_{16}O_4$

31c: Following the general procedure, 122 mg (1 mmol, 1 equiv.) starting material and 247 mg (50 mol%) electrolyte **P2** was used, the current was 15 mA. The crude mixture is purified by SiO_2 gel column chromatography with pentane/EA (from 40:1 to 20:1). 116 mg product was obtained by 52% isolated yield as yellow oil.

^{1}H NMR (600 MHz, Chloroform-*d*) δ 8.03 (d, *J* = 8.8 Hz, 2H), 6.93 (d, *J* = 8.9 Hz, 2H), 5.40 – 5.26 (m, 1H), 3.87 (s, 3H), 3.61 (dd, *J* = 10.6, 6.0 Hz, 1H), 3.53 (dd, *J* = 10.6, 4.1 Hz, 1H), 3.42 (s, 3H), 1.37 (d, *J* = 6.4 Hz, 3H).

^{13}C NMR (151 MHz, Chloroform-*d*) δ 165.99, 163.45, 131.80, 123.13, 113.65, 75.35, 69.69, 59.35, 55.54, 16.91.

ESI-HRMS: mass spectrometry: m/z calc. 247.09408 $[C_{12}H_{16}O_4Na]^+$, measured 247.09375.

IR (neat, cm^{-1}): ṽ: 2931, 2830, 2726, 1690, 1595, 1480, 1383, 1299, 1226, 1131, 1005, 966, 877, 775, 746, 678.

ee: 95.7%, enantiomeric excess was determined by HPLC with ADH column, hexane/isopropanol = 98/2, 25 °C, 1.0 mL/min, 0.1 MPa, detected by 254 nm wavelength.

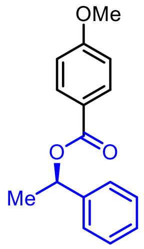

Chemical Formula: $C_{16}H_{16}O_3$

32c: Following the general procedure, 122 mg (1 mmol, 1 equiv.) starting material and 495 mg (1.0 equiv.) electrolyte **P2** was used, the current was 10 mA. The crude mixture is purified by SiO_2 gel column chromatography with pentane/EA (from 40:1 to 20:1). 130 mg product was obtained by 51%

isolated yield as colorless oil.

^{1}H NMR (600 MHz, Chloroform-*d*) δ 7.96 (d, *J* = 8.8 Hz, 2H), 7.40 – 7.34 (m, 2H), 7.32 – 7.27 (m, 2H), 7.24 – 7.20 (m, 1H), 6.84 (d, *J* = 8.9 Hz, 2H), 6.04 (q, *J* = 6.6 Hz, 1H), 3.78 (s, 3H), 1.58 (d, *J* = 6.6 Hz, 3H).

^{13}C NMR (151 MHz, Chloroform-*d*) δ 165.71, 163.51, 142.19, 131.82, 128.67, 127.94, 126.17, 123.14, 113.72, 72.67, 55.57, 22.60.

ESI-HRMS: mass spectrometry: m/z calc. 279.09917 $[C_{16}H_{16}O_3Na]^+$, measured 279.09872.

IR (neat, cm^{-1}): ṽ: 2976, 2935, 2840, 1709, 1605, 1508, 1453, 1319, 1254, 1166, 1100, 1061, 1028, 847, 764, 697.

ee: 97.9%, enantiomeric excess was determined by HPLC with ADH column, hexane/isopropanol = 98/2, 25 °C, 1.0 mL/min, 0.1 MPa, detected by 254 nm wavelength.

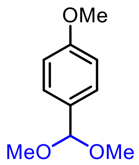

Chemical Formula: $C_{10}H_{14}O_3$

1e: Following the general procedure, 122 mg (1 mmol, 1 equiv.) starting material and 207 mg (50 mol%) electrolyte **N2** was used, the current was 5 mA. The crude mixture is purified by SiO_2 gel (washed by Et_3N) column chromatography with pentane/EA (from 60:1 to 40:1). 113 mg product was obtained by 62% isolated yield as colorless oil.

^{1}H NMR (600 MHz, Chloroform-*d*) δ 7.29 (d, *J* = 8.7 Hz, 2H), 6.82 (d, *J* = 8.7 Hz, 2H), 5.28 (s, 1H), 3.74 (s, 3H), 3.24 (s, 6H).

^{13}C NMR (151 MHz, Chloroform-*d*) δ 159.82, 130.54, 128.06, 113.67, 103.21, 55.38, 52.73.

EI-HRMS: mass spectrometry: m/z calc. 182.09375 $[C_{10}H_{14}O_3]^{\bullet+}$, measured 182.09384.

IR (neat, cm^{-1}): ṽ: 2940, 2840, 2663, 2543, 1737, 1679, 1598, 1507, 1424, 1300, 1256, 1160, 1105, 1022, 927, 834, 768, 696.

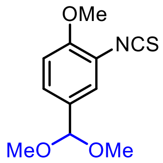

Chemical Formula: $C_{11}H_{13}NO_3S$

2e: Following the general procedure, 179 mg (1 mmol, 1 equiv.) starting material and 247 mg (50 mol%) electrolyte **P2** was used, the current was 15 mA. The crude mixture is purified by SiO_2 gel column chromatography with pentane/EA (from 20:1 to 10:1). 112 mg product was obtained by 47% isolated yield as white solid.

^{1}H NMR (600 MHz, Chloroform-*d*) δ 7.23 (dd, *J* = 8.6, 2.1 Hz, 1H), 7.15 (d, *J* = 2.1 Hz, 1H), 6.82 (d, *J* = 8.6 Hz, 1H), 5.24 (s, 1H), 3.84 (s, 3H), 3.22 (s, 6H).

^{13}C NMR (151 MHz, Chloroform-*d*) δ 155.99, 139.99, 131.12, 126.64, 124.22, 120.63, 111.20, 102.04, 56.21, 52.67.

ESI-HRMS: mass spectrometry: m/z calc. 262.05084 $[C_{11}H_{13}O_3NSNa]^+$, measured 262.05154.

IR (neat, cm^{-1}): ṽ: 2938, 2832, 2057, 1723, 1608, 1504, 1443, 1351, 1275, 1232, 1190, 1164, 1134, 1099, 1053, 1025, 980, 894, 812, 787, 764, 693.

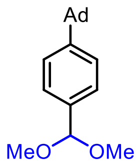

Chemical Formula: $C_{19}H_{26}O_2$

3e: Following the general procedure, 226 mg (1 mmol, 1 equiv.) starting material and 207 mg (50 mol%) electrolyte **N2** was used, the current was 15 mA. The crude mixture is purified by SiO_2 gel (washed by Et_3N) column chromatography with pentane. 120 mg product was obtained by 42% isolated yield as white solid.

^{1}H NMR (600 MHz, Chloroform-*d*) δ 7.45 – 7.34 (m, 4H), 5.39 (s, 1H), 3.36 (s, 6H), 2.15 – 2.10 (m, 3H), 1.95 – 1.93 (m, 6H), 1.84 – 1.76 (m, 6H).

^{13}C NMR (151 MHz, Chloroform-*d*) δ 151.80, 135.32, 126.51, 124.86, 103.59, 53.00, 43.31, 36.94, 36.27, 29.09.

EI-HRMS: mass spectrometry: m/z calc. 286.19273 $[C_{19}H_{26}O_2]^{\bullet+}$, measured 286.19237.

IR (neat, cm^{-1}): ṽ: 2904, 2849, 1722, 1607, 1512, 1448, 1408, 1352, 1318, 1277, 1191, 1101 1049, 1016, 981, 913, 827, 799, 770, 704, 667.

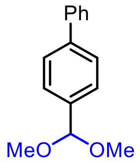

Chemical Formula: $C_{15}H_{16}O_2$

4e: Following the general procedure, 168 mg (1 mmol, 1 equiv.) starting material and 207 mg (50 mol%) electrolyte **N2** was used, the current was 15 mA. The crude mixture is purified by SiO_2 gel column chromatography with pentane/EA (from 50:1 to 30:1). 92 mg product was obtained by 40% isolated yield as white solid.

^{1}H NMR (600 MHz, Chloroform-*d*) δ 7.55 – 7.49 (m, 4H), 7.47 – 7.42 (m, 2H), 7.37 – 7.33 (m, 2H), 7.30 – 7.23 (m, 1H), 5.36 (s, 1H), 3.28 (s, 6H).

^{13}C NMR (151 MHz, Chloroform-*d*) δ 141.46, 140.94, 137.24, 128.90, 127.50, 127.27, 127.10, 103.18, 52.87.

EI-HRMS: mass spectrometry: m/z calc. 228.11448 $[C_{15}H_{16}O_2Na]^{\bullet+}$, measured 228.11513.

IR (neat, cm^{-1}): ṽ: 3030, 2990, 2938, 2828, 2326, 2087, 1915, 1808, 1702, 1603, 1566, 1485, 1447, 1403, 1353, 1310, 1279, 1211, 1189, 1100, 1052, 982, 910, 826, 760, 697.

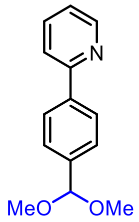

Chemical Formula: $C_{14}H_{15}NO_2$

5e: Following the general procedure, 169 mg (1 mmol, 1 equiv.) starting material and 207 mg (50 mol%) electrolyte **N2** was used, the current was 15 mA. The crude mixture is purified by SiO_2 gel

column chromatography with pentane/EA (from 10:1 to 5:1). 150 mg product was obtained by 66% isolated yield as colorless oil.

^{1}H NMR (600 MHz, Chloroform-*d*) δ 8.71 (d, *J* = 4.6, 1.4 Hz, 1H), 8.02 (d, *J* = 8.0 Hz, 2H), 7.80 – 7.72 (m, 2H), 7.58 (d, *J* = 8.0 Hz, 2H), 7.30 – 7.18 (m, 1H), 5.49 (s, 1H), 3.37 (s, 6H).

^{13}C NMR (151 MHz, Chloroform-*d*) δ 157.25, 149.84, 139.65, 138.90, 136.87, 127.29, 126.89, 122.31, 120.72, 102.93, 52.73.

ESI-HRMS: mass spectrometry: m/z calc. 230.11756 $[C_{14}H_{16}O_2N]^+$, measured 230.11775.

IR (neat, cm^{-1}): ṽ: 3053, 2991, 2937, 2829, 2326, 2086, 1584, 1465, 1436, 1403, 1353, 1300, 1269, 1209, 1154, 1099, 1052, 982, 909, 833, 778, 670.

6. Preparation of the esters from aldehydes

General procedure for the esterification of aldehydes.

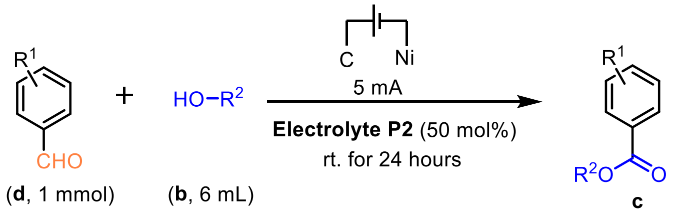

Unless otherwise specified, the aldehyde **d** (1.0 mmol scale) and the electrolyte **P2** (0.5 mmol) are added into the alcohol **b** (6 mL) in a reaction vial. The reaction vial is then sealed with aluminous headspace cap with electrodes (see the pictures above). The current is set to 5 mA. Keep the reaction stirring in room temperature for 24 hours. After that, the alcohol is removed and the crude is directly engaged on SiO_2 gel column chromatography for purification.

1c: Following the general procedure, 136 mg (1 mmol, 1 equiv.) starting material was used. The crude mixture was purified by SiO_2 gel column chromatography with pentane/EA (from 40:1 to 20:1). 120 mg product was obtained by 72% isolated yield as white solid.

2c: Following the general procedure, 215 mg (1 mmol, 1 equiv.) starting material was used. The crude mixture is purified by SiO_2 gel column chromatography with pentane/EA (20:1). 180 mg product was obtained by 74% isolated yield as grey solid.

8c: Following the general procedure, 185 mg (1 mmol, 1 equiv.) starting material was used. The crude mixture is purified by SiO_2 gel column chromatography with DCM. 30 mg product was obtained by 14% isolated yield as white solid.

16c: Following the general procedure, 166 mg (1 mmol, 1 equiv.) starting material was used. The crude mixture is purified by SiO_2 gel column chromatography with pentane/EA (from 5:1 to 2:1). 130 mg product was obtained by 66% isolated yield as yellow solid.

21c: Following the general procedure, 136 mg (1 mmol, 1 equiv.) starting material was used. The

crude mixture is purified by SiO_2 gel column chromatography with pentane/EA (30:1). 140 mg product was obtained by 78% isolated yield as colorless oil.

22c: Following the general procedure, 136 mg (1 mmol, 1 equiv.) starting material was used. The crude mixture is purified by SiO_2 gel column chromatography with pentane/EA (from 40:1 to 20:1). 140 mg product was obtained by 72% isolated yield as yellow oil.

23c: Following the general procedure, 136 mg (1 mmol, 1 equiv.) starting material was used. The crude mixture is purified by SiO_2 gel column chromatography with pentane/EA (from 40:1 to 20:1). 140 mg product was obtained by 67% isolated yield as colorless oil.

24c: Following the general procedure, 136 mg (1 mmol, 1 equiv.) starting material was used. The crude mixture is purified by SiO_2 gel column chromatography with pentane/EA (from 40:1 to 20:1). 100 mg product was obtained by 45% isolated yield as yellow oil.

25c: Following the general procedure, 136 mg (1 mmol, 1 equiv.) starting material was used. The crude mixture is purified by SiO_2 gel column chromatography with pentane/EA (from 40:1 to 20:1). 135 mg product was obtained by 65% isolated yield as colorless oil.

26c: Following the general procedure, 136 mg (1 mmol, 1 equiv.) starting material was used. The crude mixture is purified by SiO_2 gel column chromatography with pentane/EA (from 40:1 to 20:1). 130 mg product was obtained by 62% isolated yield as colorless oil.

31c: Following the general procedure, 136 mg (1 mmol, 1 equiv.) starting material was used. The crude mixture is purified by SiO_2 gel column chromatography with pentane/EA (from 40:1 to 20:1). 130 mg product was obtained by 58% isolated yield as yellow oil.

32c: Following the general procedure, 136 mg (1 mmol, 1 equiv.) starting material was used. The crude mixture is purified by SiO_2 gel column chromatography with pentane/EA (from 40:1 to 20:1). 120 mg product was obtained by 47% isolated yield as colorless oil.

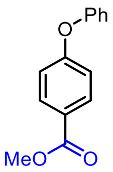

Chemical Formula: $C_{14}H_{12}O_3$

33c: Following the general procedure, 198 mg (1 mmol, 1 equiv.) starting material was used. The crude mixture is purified by SiO_2 gel column chromatography with pentane/EA (from 40:1 to 20:1). 160 mg product was obtained by 70% isolated yield as yellow solid.

^{1}H NMR (600 MHz, Chloroform-*d*) δ 7.92 (d, *J* = 8.9 Hz, 2H), 7.36 – 7.27 (m, 2H), 7.16 – 7.08 (m, 1H), 7.02 – 6.97 (m, 2H), 6.90 (d, *J* = 8.8 Hz, 2H), 3.82 (s, 3H).

^{13}C NMR (151 MHz, Chloroform-*d*) δ 166.73, 161.95, 155.77, 131.81, 130.15, 124.62, 120.23, 117.42, 52.12.

ESI-HRMS: mass spectrometry: m/z calc. 251.06787 $[C_{14}H_{12}O_3Na]^+$, measured 251.06774.

IR (neat, cm^{-1}): ṽ: 3403, 3075, 3016, 2946, 2849, 2325, 2160, 2073, 1992, 1927, 1796, 1790, 1588, 1484, 1434, 1246, 1193, 1158, 1101, 1006, 958, 912, 857, 788, 756, 691.

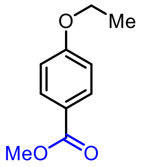

Chemical Formula: $C_{10}H_{12}O_3$

34c: Following the general procedure, 150 mg (1 mmol, 1 equiv.) starting material was used. The crude mixture is purified by SiO_2 gel column chromatography with pentane/EA (from 40:1 to 20:1). 130 mg product was obtained by 72% isolated yield as colorless oil.

^{1}H NMR (600 MHz, Chloroform-*d*) δ 7.96 – 7.84 (m, 2H), 6.88 – 6.76 (m, 2H), 3.98 (q, *J* = 7.0 Hz, 2H), 3.79 (s, 3H), 1.34 (t, *J* = 7.0 Hz, 3H).

^{13}C NMR (151 MHz, Chloroform-*d*) δ 166.95, 162.85, 131.64, 122.47, 114.11, 63.73, 51.86, 14.73.

ESI-HRMS: mass spectrometry: m/z calc. 203.06787 $[C_{10}H_{12}O_3Na]^+$, measured 203.06757.

IR (neat, cm^{-1}): ṽ: 3420, 2983, 2949, 2327, 2083, 1991, 1917, 1717, 1604, 1510, 1475, 1434, 1394, 1312, 1250, 1167, 1106, 1042, 966, 921, 848, 769, 695.

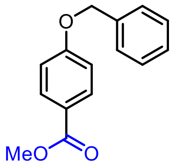

Chemical Formula: $C_{15}H_{14}O_3$

35c: Following the general procedure, 212 mg (1 mmol, 1 equiv.) starting material was used. The crude mixture is purified by SiO_2 gel column chromatography with pentane/EA (from 40:1 to 20:1). 200 mg product was obtained by 83% isolated yield as yellow solid.

^{1}H NMR (600 MHz, Chloroform-*d*) δ 7.97 – 7.85 (m, 2H), 7.40 – 7.23 (m, 5H), 6.94 – 6.87 (m, 2H), 5.03 (s, 2H), 3.79 (s, 3H).

^{13}C NMR (151 MHz, Chloroform-*d*) δ 166.93, 162.61, 136.39, 131.74, 128.80, 128.33, 127.61, 122.98, 114.59, 70.21, 51.97.

ESI-HRMS: mass spectrometry: m/z calc. 265.08352 $[C_{15}H_{14}O_3Na]^+$, measured 265.08309.

IR (neat, cm^{-1}): ṽ: 3745, 3403, 2952, 2325, 2017, 1805, 1710, 1600, 1507, 1437, 1386, 1315, 1274, 1246, 1116, 1111, 1006, 970, 853, 747, 696, 656.

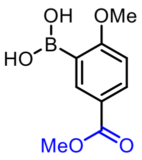

Chemical Formula: $C_9H_{11}BO_5$

36c: Following the general procedure, 180 mg (1 mmol, 1 equiv.) starting material was used. The crude mixture is purified by SiO_2 gel column chromatography with pentane/EA (from 10:1 to 2:1). 125 mg product was obtained by 60% isolated yield as white solid.

^{1}H NMR (600 MHz, Chloroform-*d*) δ 8.47 (d, *J* = 2.4 Hz, 1H), 8.08 (dd, *J* = 8.7, 2.4 Hz, 1H), 6.88 (d, *J* = 8.7 Hz, 1H), 6.02 (s, 2H), 3.91 (s, 3H), 3.83 (s, 3H).

^{13}C NMR (151 MHz, Chloroform-*d*) δ 167.94, 166.90, 138.90, 135.00, 123.38, 109.98, 56.04, 52.07.

EI-HRMS: mass spectrometry: m/z calc. 210.06941 $[C_9H_{11}O_5B]^{\bullet+}$, measured 210.06974.

IR (neat, cm^{-1}): ṽ: 3535, 3465, 2950, 2160, 1694, 1597, 1416, 1385, 1249, 1123, 1080, 1028, 969, 826, 770, 714.

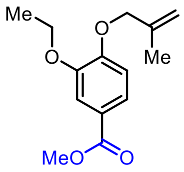

Chemical Formula: $C_{14}H_{18}O_4$

37c: Following the general procedure, 220 mg (1 mmol, 1 equiv.) starting material was used. The crude mixture is purified by SiO_2 gel column chromatography with pentane/EA (from 40:1 to 20:1). 110 mg product was obtained by 44% isolated yield as colorless oil.

^{1}H NMR (600 MHz, Chloroform-*d*) δ 7.55 (dd, *J* = 8.4, 2.0 Hz, 1H), 7.48 (d, *J* = 2.0 Hz, 1H), 6.80 (d, *J* = 8.4 Hz, 1H), 5.07 – 5.00 (m, 1H), 4.96 – 4.89 (m, 1H), 4.48 (broad s, 2H), 4.07 (q, *J* = 7.0 Hz, 2H), 3.81 (s, 3H), 1.76 (broad s, 3H), 1.39 (t, *J* = 7.0 Hz, 3H).

^{13}C NMR (151 MHz, Chloroform-*d*) δ 167.08, 152.70, 140.37, 123.51, 122.97, 114.28, 112.91, 112.71, 72.55, 64.77, 52.07, 19.39, 14.90.

APCI-HRMS: mass spectrometry: m/z calc. 251.12779 $[C_{14}H_{19}O_4]^+$, measured 251.12889.

IR (neat, cm^{-1}): ṽ: 3420, 3083, 2980, 2926, 2325, 2085, 1714, 1594, 1511, 1436, 1392, 1266, 1207, 1134, 1107, 1041, 1002, 903, 763, 728.

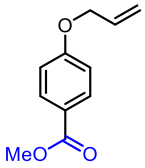

Chemical Formula: $C_{11}H_{12}O_3$

38c: Following the general procedure, 162 mg (1 mmol, 1 equiv.) starting material was used. The crude mixture is purified by SiO_2 gel column chromatography with pentane/EA (from 40:1 to 20:1). 130 mg product was obtained by 68% isolated yield as yellow oil.

^{1}H NMR (600 MHz, Chloroform-*d*) δ 7.98 – 7.85 (m, 2H), 6.92 – 6.78 (m, 2H), 6.04 – 5.92 (m, 1H), 5.34 (dq, *J* = 17.3, 1.5 Hz, 1H), 5.23 (dq, *J* = 10.5, 1.4 Hz, 1H), 4.51 (dt, *J* = 5.3, 1.6 Hz, 2H), 3.80 (s, 3H).

^{13}C NMR (151 MHz, Chloroform-*d*) δ 166.95, 162.46, 132.70, 131.69, 122.86, 118.22, 114.44, 68.96, 51.95.

ESI-HRMS: mass spectrometry: m/z calc. 215.06787 $[C_{11}H_{12}O_3Na]^+$, measured 215.06744.

IR (neat, cm^{-1}): ṽ: 3420, 3082, 2950, 2326, 2087, 1915, 1714, 1603, 1509, 1433, 1250, 1168, 1106, 998, 928, 846, 769, 695, 667.

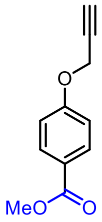

Chemical Formula: $C_{11}H_{10}O_3$

39c: Following the general procedure, 160 mg (1 mmol, 1 equiv.) starting material was used. The

crude mixture is purified by SiO_2 gel column chromatography with pentane/EA (from 40:1 to 20:1). 135 mg product was obtained by 71% isolated yield as white solid.

^{1}H NMR (600 MHz, Chloroform-*d*) δ 7.93 (d, *J* = 8.8 Hz, 2H), 6.92 (d, *J* = 8.9 Hz, 2H), 4.66 (d, *J* = 2.4 Hz, 2H), 3.81 (s, 3H), 2.48 (t, *J* = 2.4 Hz, 1H).

^{13}C NMR (151 MHz, Chloroform-*d*) δ 166.80, 161.28, 131.66, 123.58, 114.60, 77.94, 76.20, 55.93, 52.01.

ESI-HRMS: mass spectrometry: m/z calc. 213.05222 $[C_{11}H_{10}O_3Na]^+$, measured 213.05193.

IR (neat, cm^{-1}): ṽ: 3399, 3243, 2957, 2864, 2323, 2127, 1927, 1702, 1603, 1508, 1438, 1370, 1284, 1246, 1170, 1107, 1016, 958, 850, 814, 768, 734, 695, 659.

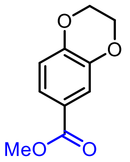

Chemical Formula: $C_{10}H_{10}O_4$

40c: Following the general procedure, 164 mg (1 mmol, 1 equiv.) starting material was used. The crude mixture is purified by SiO_2 gel column chromatography with pentane/EA (from 40:1 to 20:1). 130 mg product was obtained by 67% isolated yield as colorless oil.

^{1}H NMR (600 MHz, Chloroform-*d*) δ 7.52 – 7.44 (m, 2H), 6.80 (d, *J* = 8.2 Hz, 1H), 4.28 – 4.14 (m, 4H), 3.79 (s, 3H).

^{13}C NMR (151 MHz, Chloroform-*d*) δ 166.71, 147.87, 143.27, 123.53, 123.50, 119.10, 117.20, 64.71, 64.18, 52.02.

ESI-HRMS: mass spectrometry: m/z calc. 217.04713 $[C_{10}H_{10}O_4Na]^+$, measured 217.04659.

IR (neat, cm^{-1}): ṽ: 3418, 2948, 2883, 2646, 2328, 2081, 1712, 1585, 1435, 1287, 1192, 1095, 1062, 985, 934, 888, 830, 763, 716, 677.

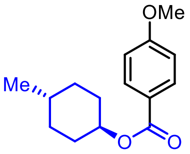

Chemical Formula: $C_{15}H_{20}O_3$

41c: Following the general procedure, 136 mg (1 mmol, 1 equiv.) starting material was used. The crude mixture is purified by SiO_2 gel column chromatography with pentane/EA (from 40:1 to 20:1). 130 mg product was obtained by 52% isolated yield as white solid.

^{1}H NMR (600 MHz, Chloroform-*d*) δ 7.91 (d, *J* = 8.9 Hz, 2H), 6.83 (d, *J* = 8.9 Hz, 2H), 4.81 (tt, *J* = 11.1, 4.4 Hz, 1H), 3.78 (s, 3H), 2.04 – 1.96 (m, 2H), 1.74 – 1.67 (m, 2H), 1.45 – 1.31 (m, 3H), 1.09 – 0.99 (m, 2H), 0.85 (d, *J* = 6.6 Hz, 3H).

^{13}C NMR (151 MHz, Chloroform-*d*) δ 166.04, 163.31, 131.66, 123.58, 113.61, 73.79, 55.54, 33.21, 31.91, 22.00.

ESI-HRMS: mass spectrometry: m/z calc. 271.13047 $[C_{15}H_{20}O_3Na]^+$, measured 271.13035.

IR (neat, cm^{-1}): ṽ: 3392, 3080, 2943, 2861, 2323, 2166, 2024, 1919, 1705, 1604, 1509, 1451, 1361, 1320, 1277, 1248, 1163, 1109, 1016, 987, 934, 845, 767, 696.

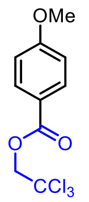

Chemical Formula: $C_{10}H_9Cl_3O_3$

42c: Following the general procedure, 136 mg (1 mmol, 1 equiv.) starting material was used. The crude mixture is purified by SiO_2 gel column chromatography with pentane/EA (from 40:1 to 20:1). 150 mg product was obtained by 53% isolated yield as colorless oil.

^{1}H NMR (600 MHz, Chloroform-*d*) δ 8.01 (d, *J* = 8.9 Hz, 2H), 6.89 (d, *J* = 8.9 Hz, 2H), 4.87 (s, 2H), 3.81 (s, 3H).

^{13}C NMR (151 MHz, Chloroform-*d*) δ 164.65, 164.11, 132.24, 121.04, 113.94, 95.31, 74.30, 55.53.

ESI-HRMS: mass spectrometry: m/z calc. 304.95095 $[C_{10}H_9O_3Cl_3Na]^+$, measured 304.95064.

IR (neat, cm^{-1}): ṽ: 3445, 2955, 2841, 2326, 2101, 1917, 1725, 1604, 1510, 1451, 1368, 1316, 1254, 1167, 1106, 1060, 1029, 845, 804, 767, 716.

7. References

[S1] M. Liu, T. Chen, Y. Zhou, S. Yin, Transition metal-free oxidative *ortho*-acylation of phenols with *N*-heteroarylmethanes *via* double C–H activation. *Catal. Sci. Technol.*, **2016,** 6, 5792-5796.

8. Copies of ^{1}H and ^{13}C spectra, HPLC spectra

^{1}H NMR (**5a**)

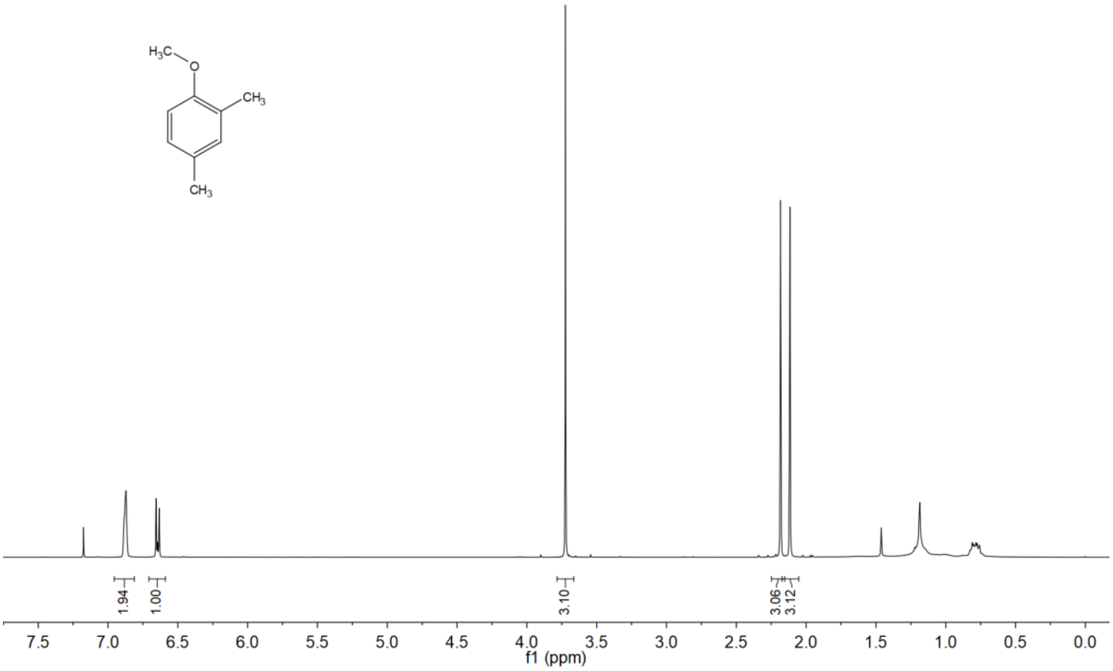

^{13}C NMR (**5a**)

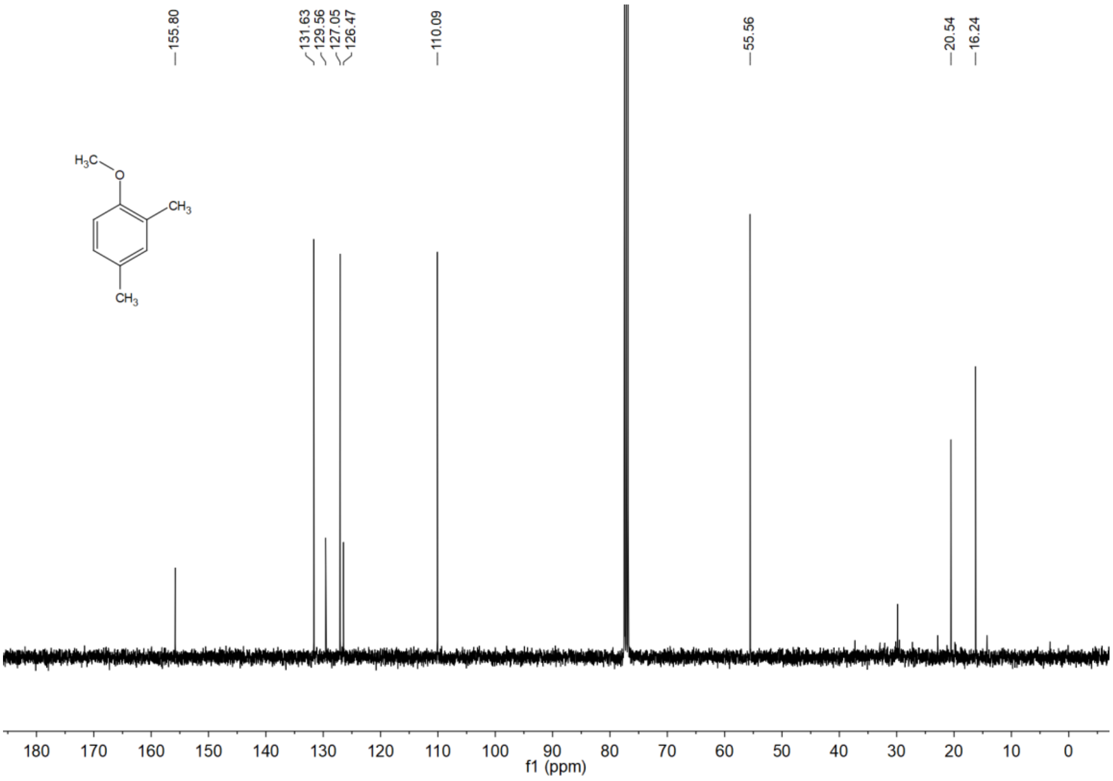

^{1}H NMR (**6a**)

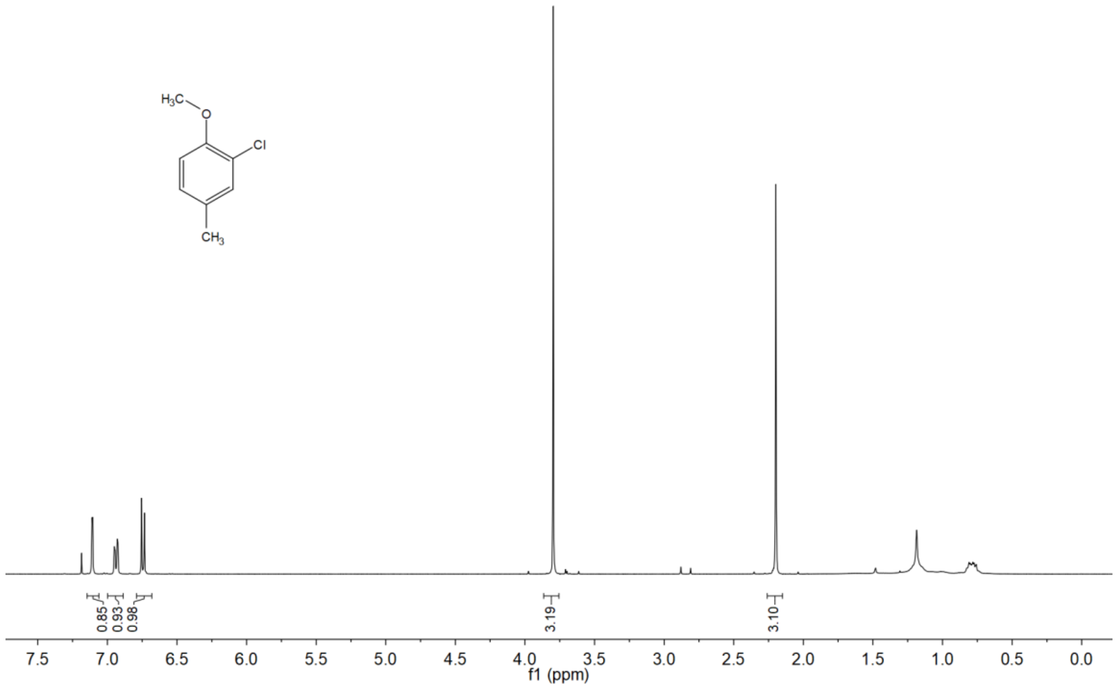

^{13}C NMR (**6a**)

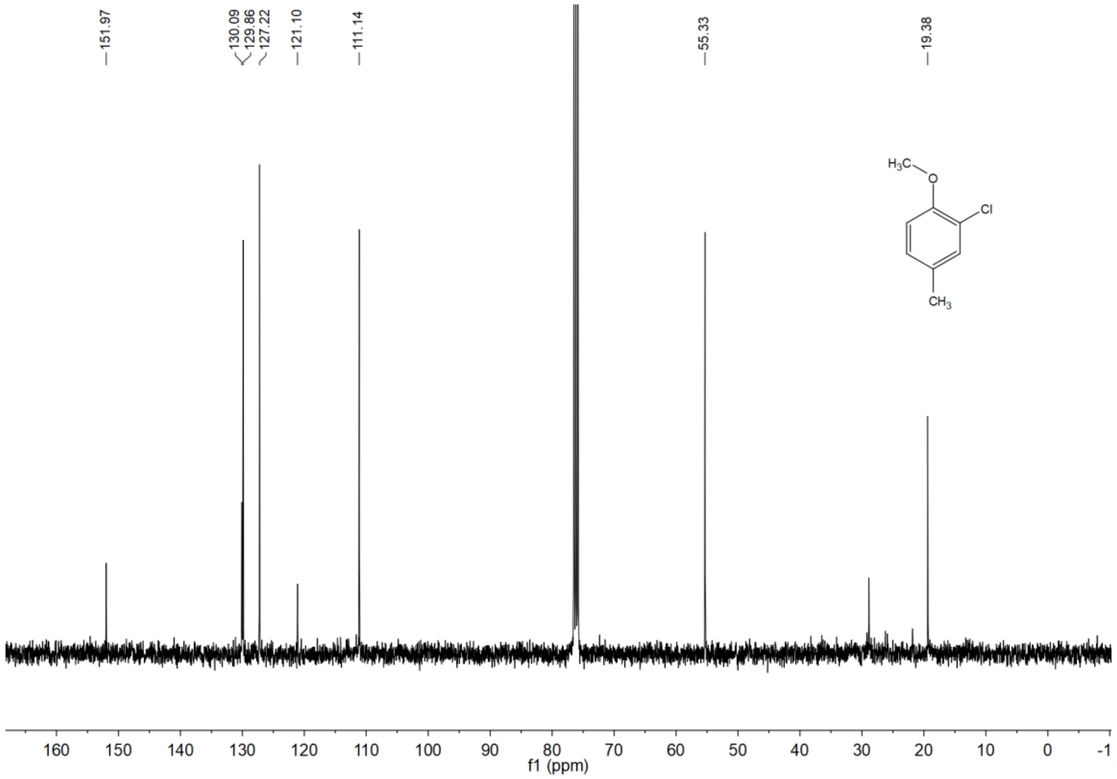

^{1}H NMR (**7a**)

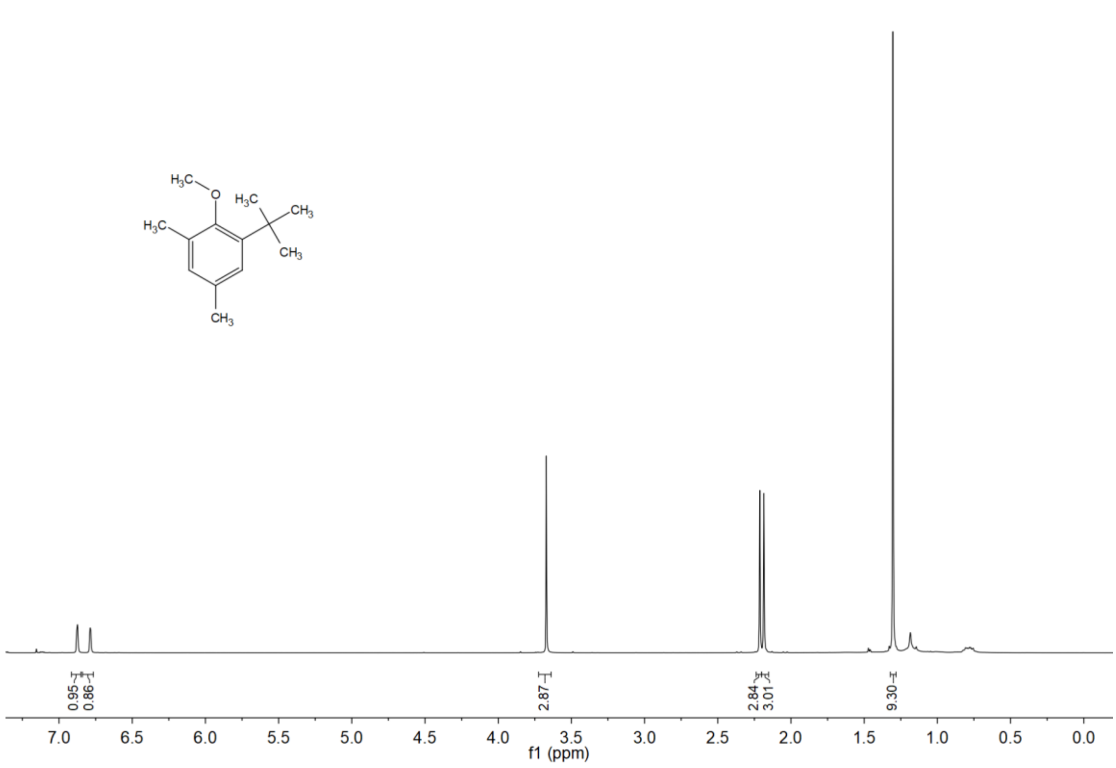

^{13}C NMR (**7a**)

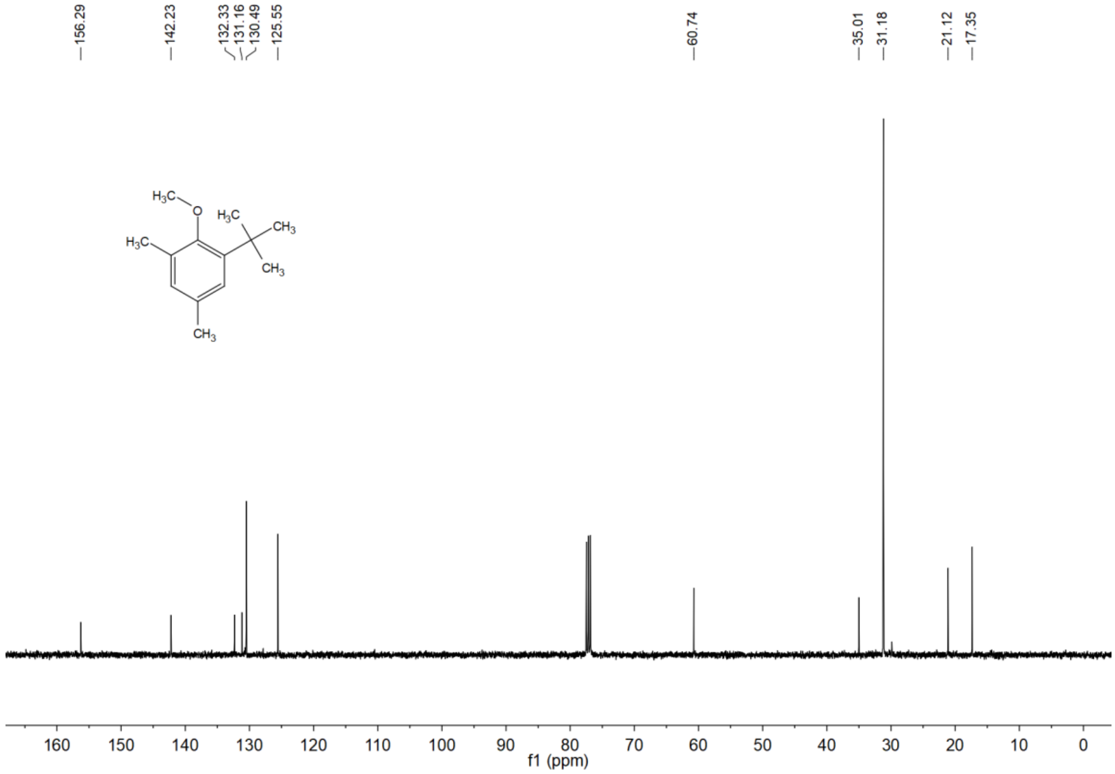

^{1}H NMR (**13a**)

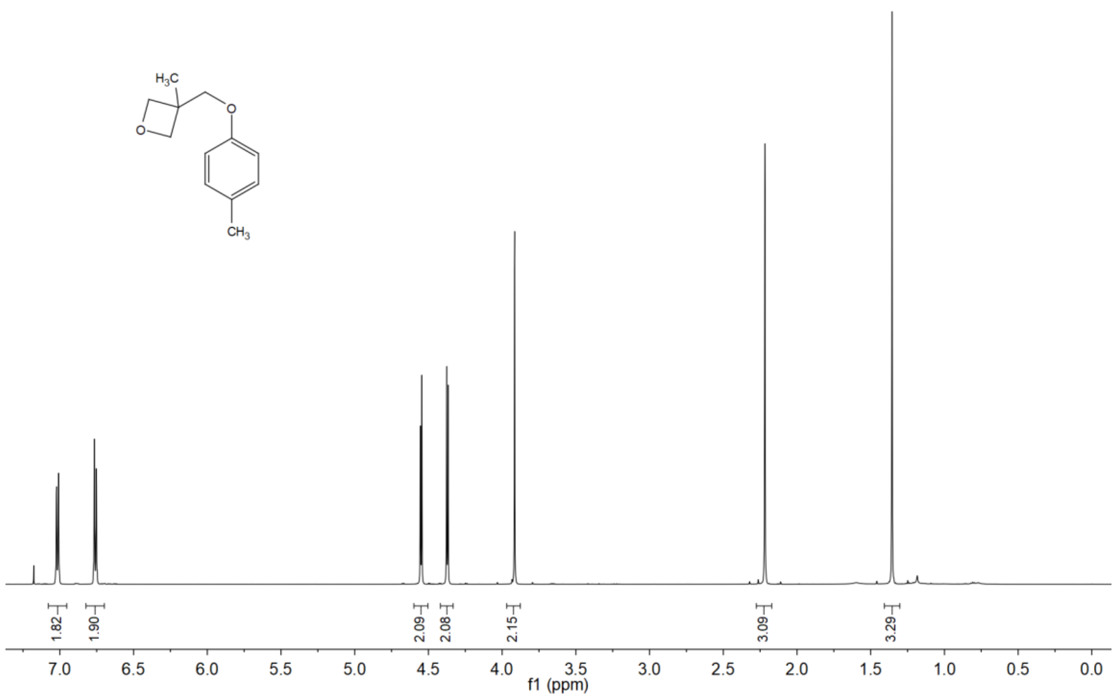

^{13}C NMR (**13a**)

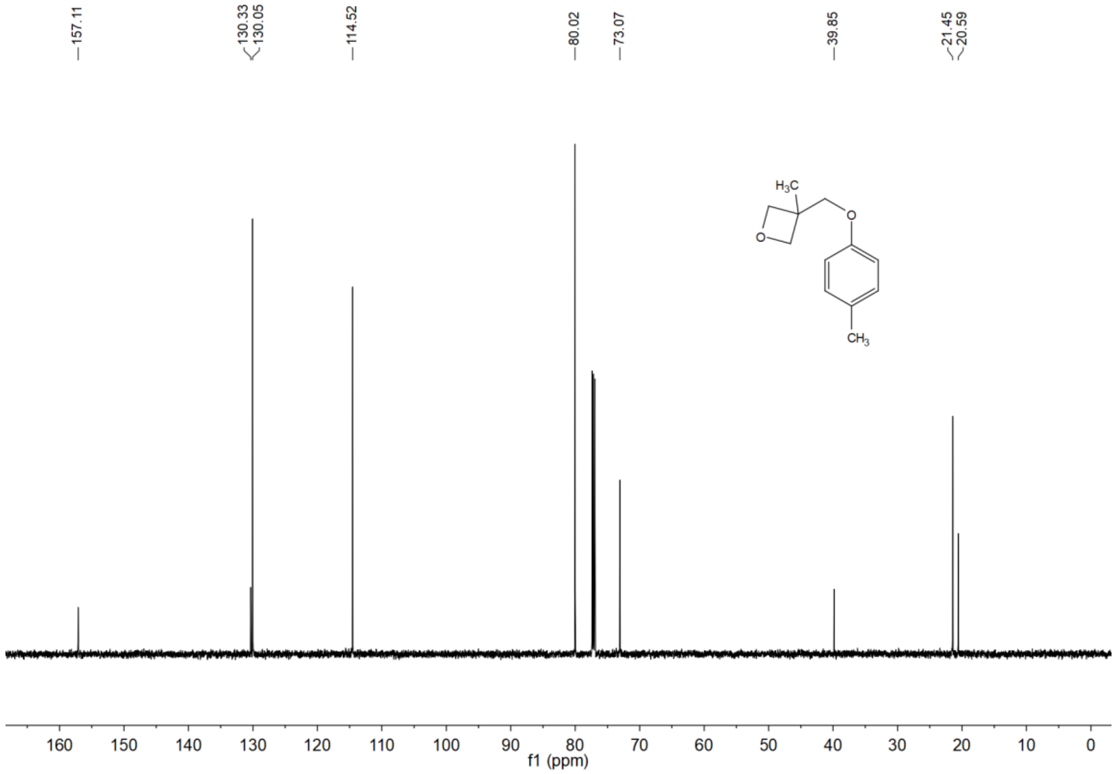

^{1}H NMR (**17a**)

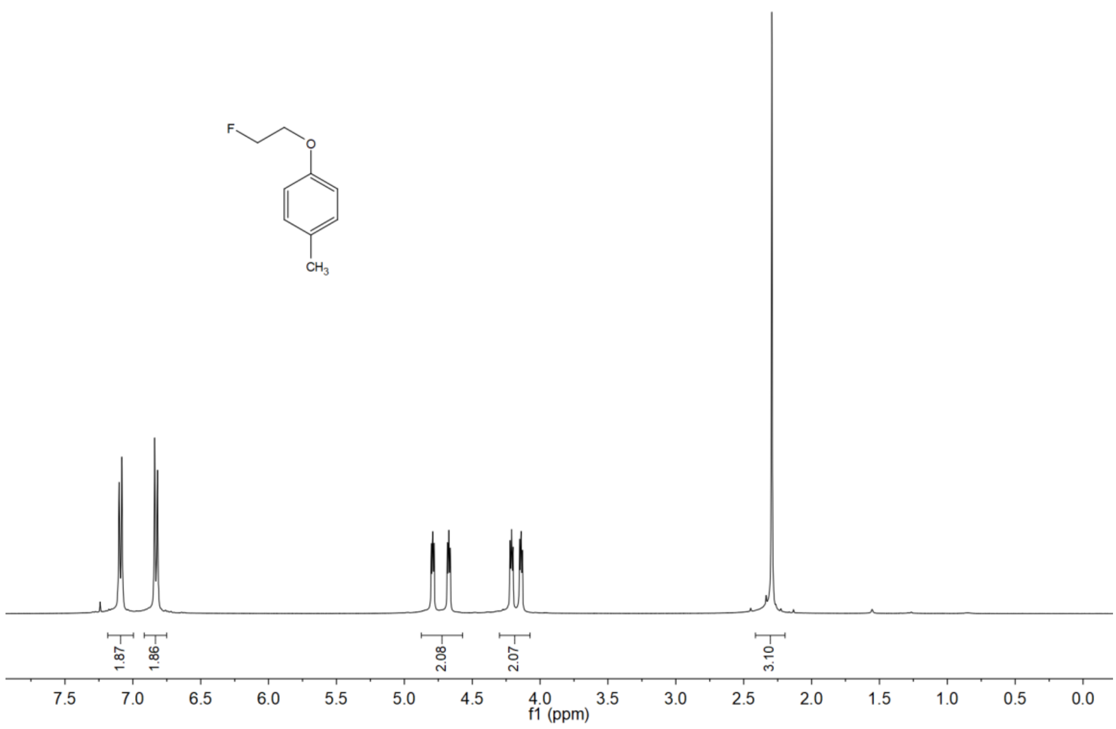

^{13}C NMR (**17a**)

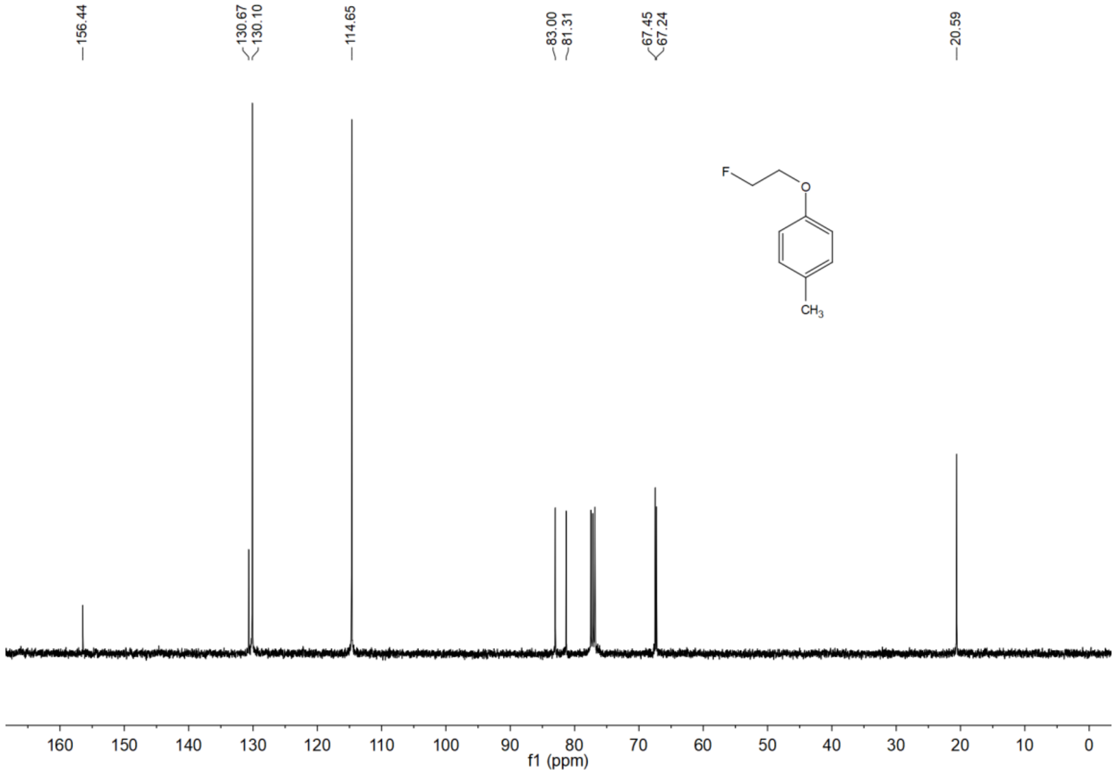

^{1}H NMR (**20a**)

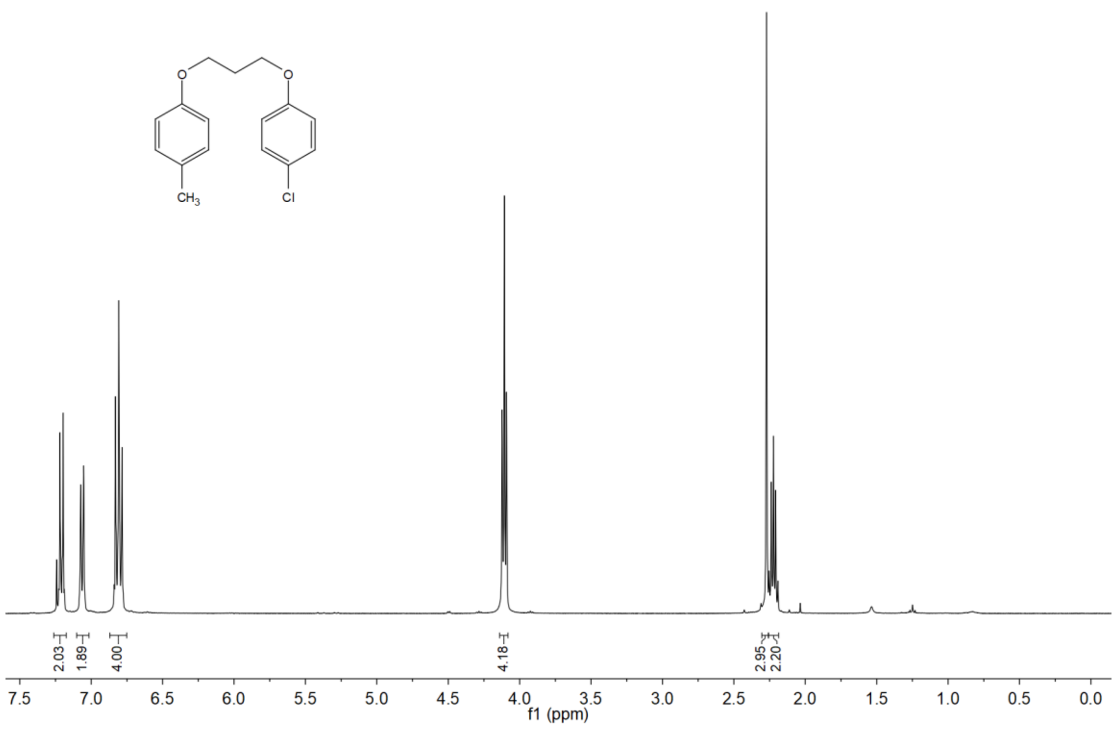

^{13}C NMR (**20a**)

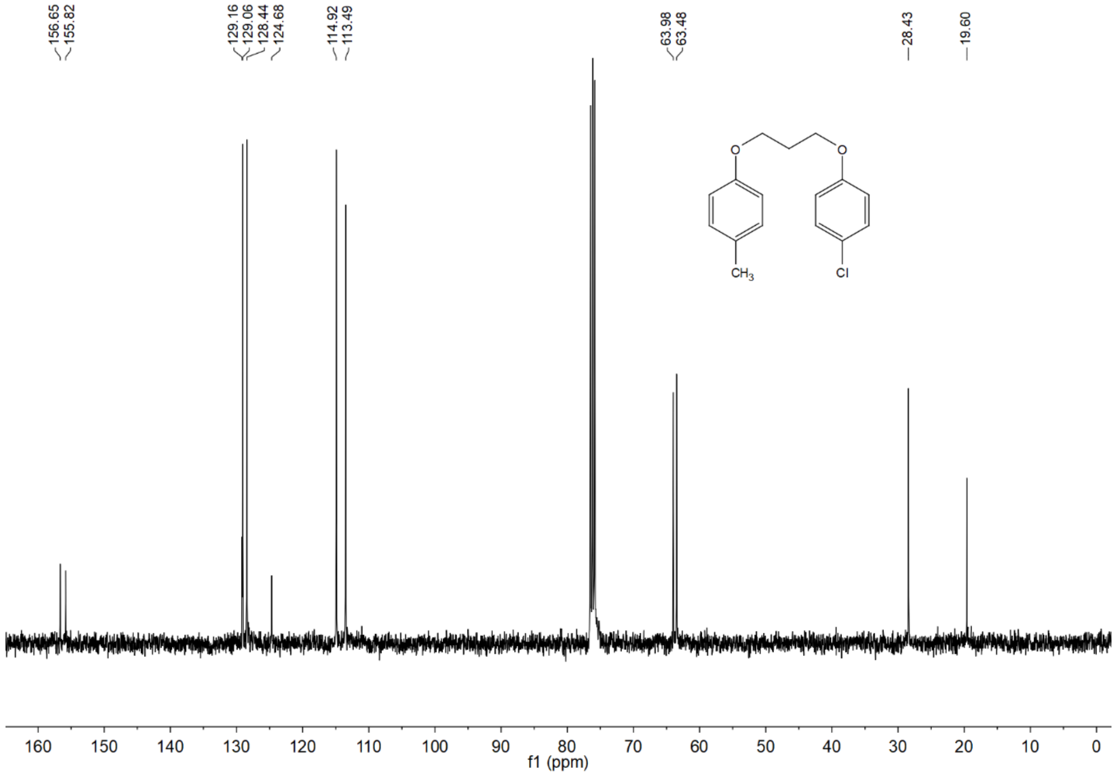

^{1}H NMR (**1c**)

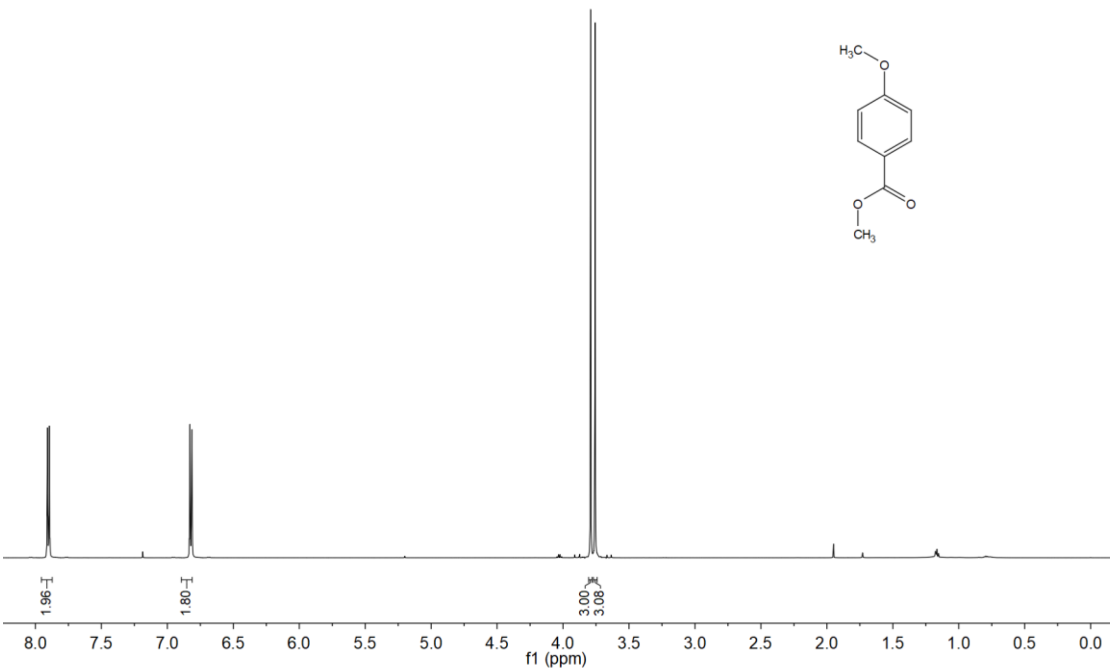

^{13}C NMR (**1c**)

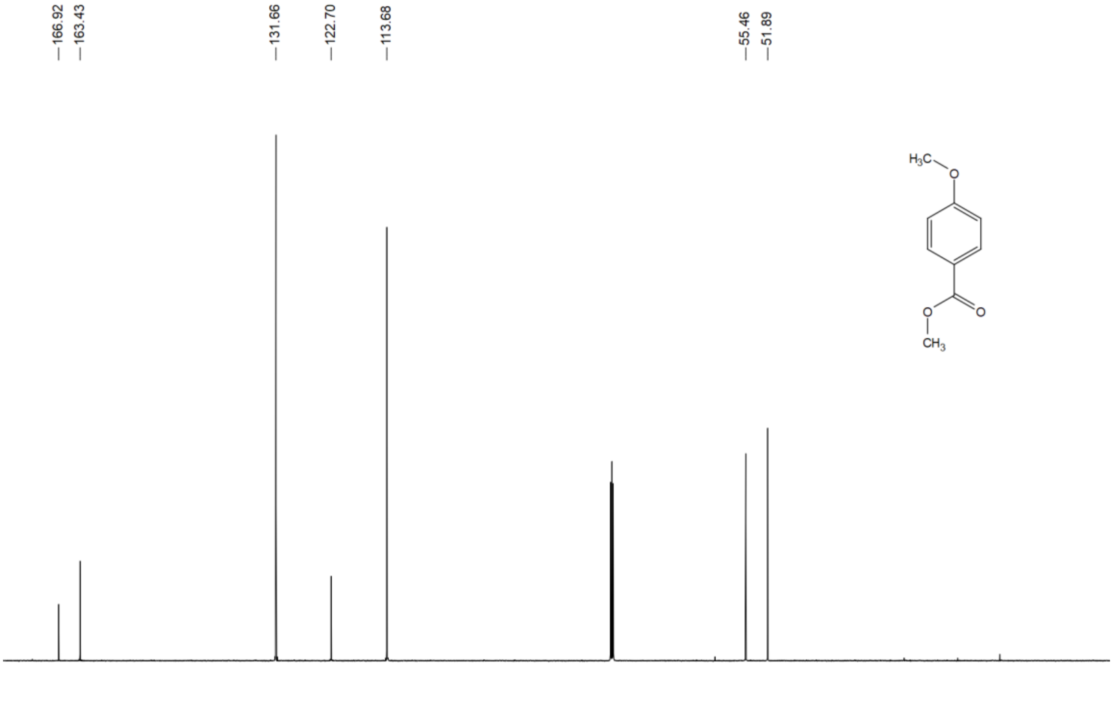

^{1}H NMR (**2c**)

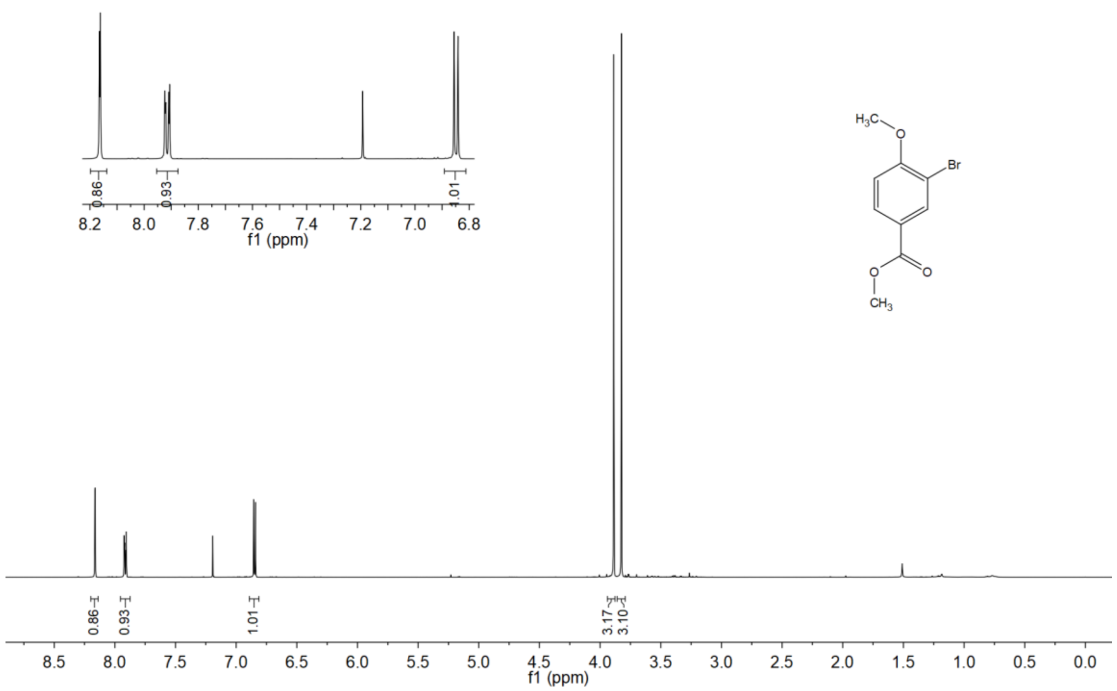

^{13}C NMR (**2c**)

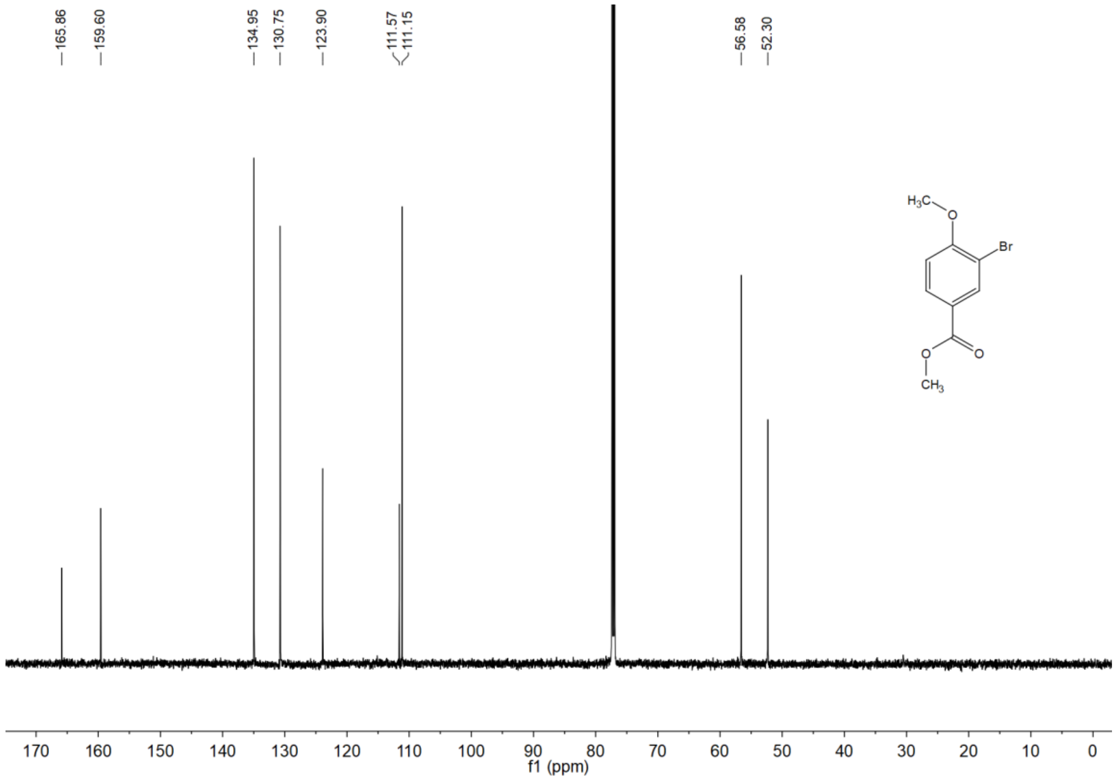

^{1}H NMR (**3c**)

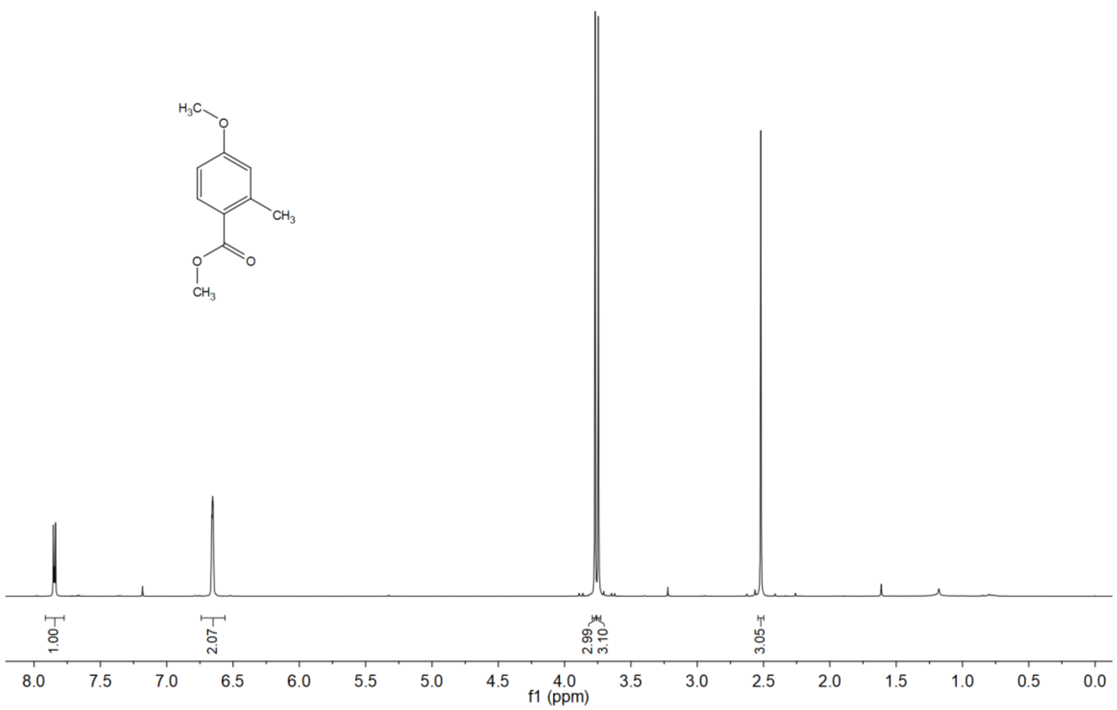

^{13}C NMR (**3c**)

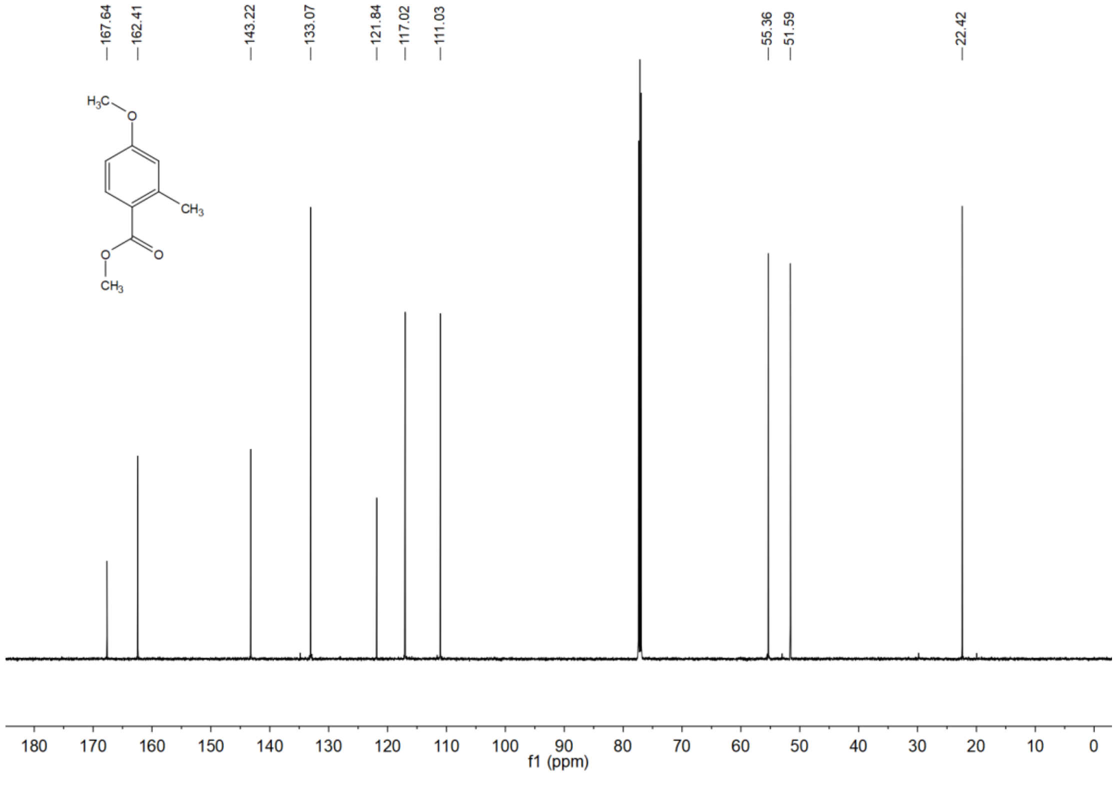

^{1}H NMR (**4c**)

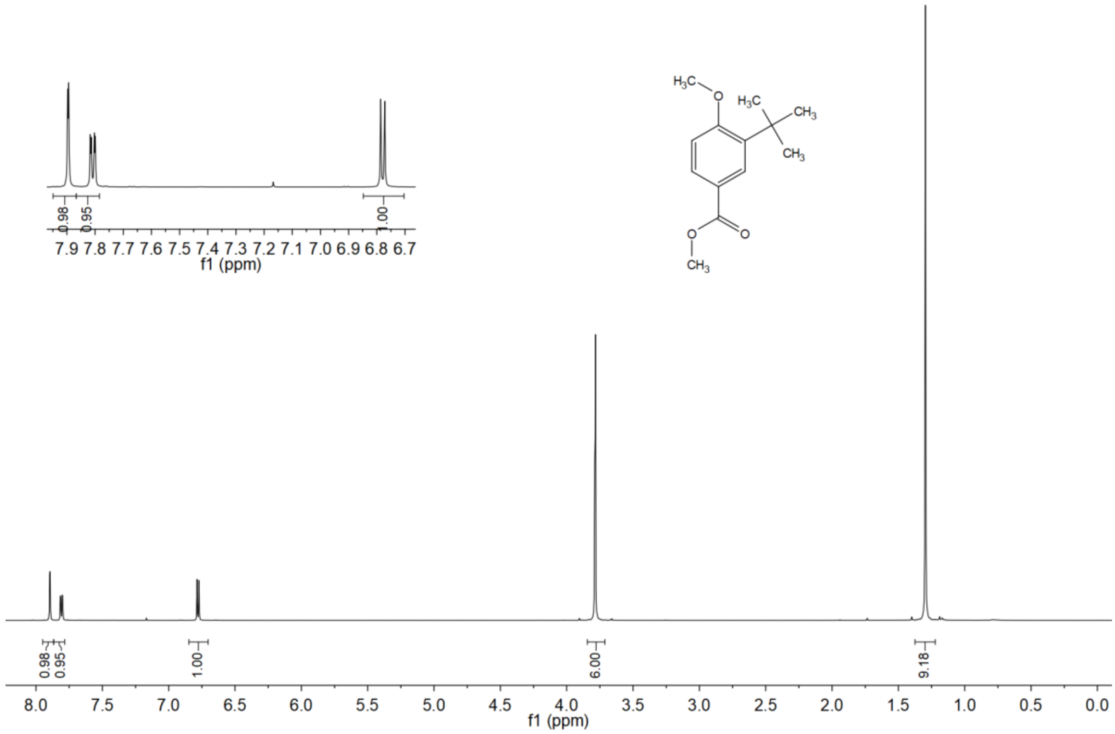

^{13}C NMR (**4c**)

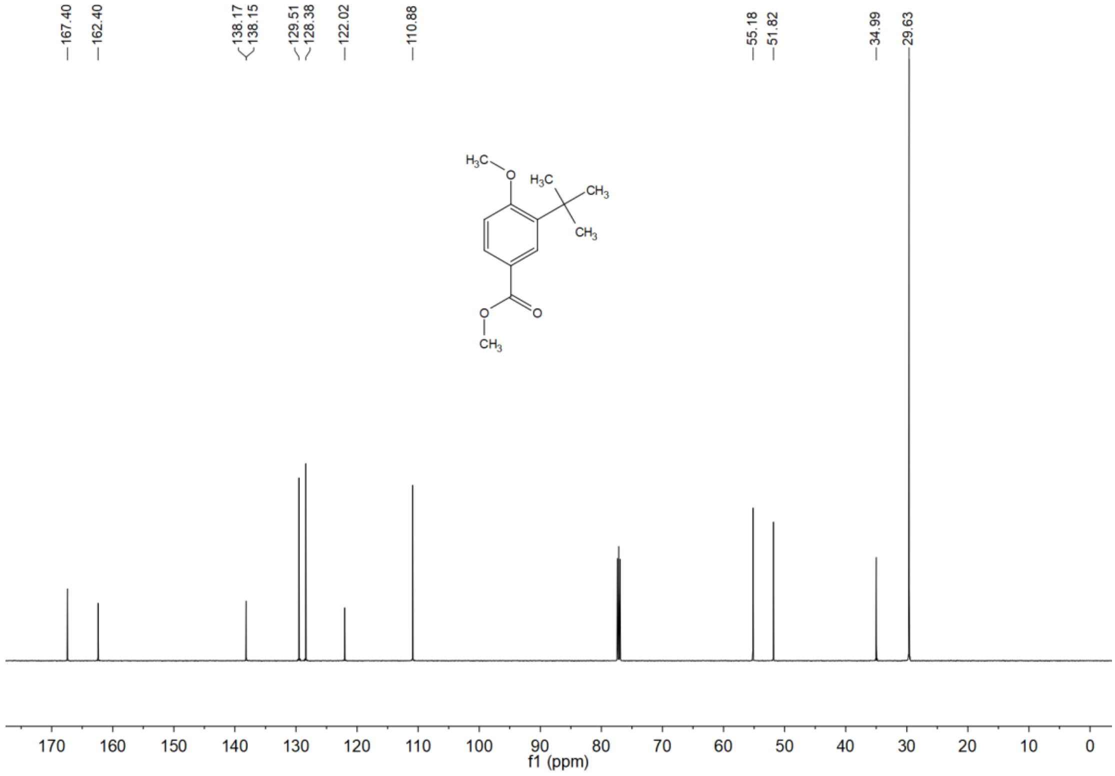

^{1}H NMR (**5c**)

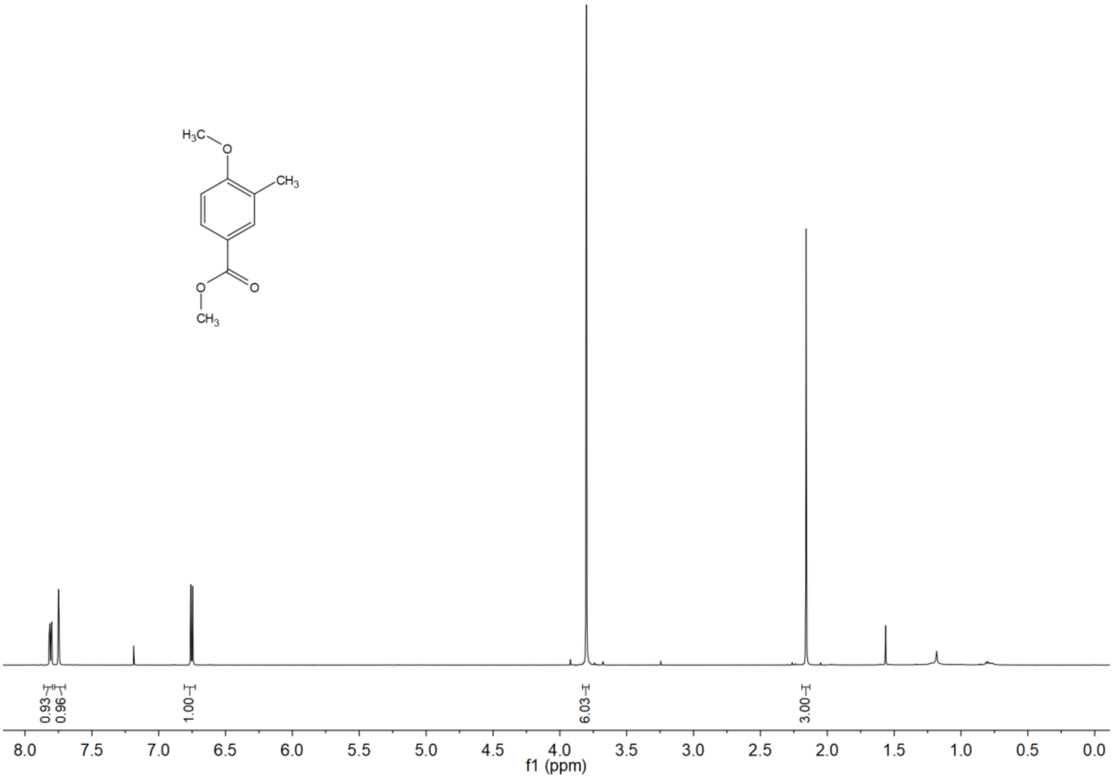

^{13}C NMR (**5c**)

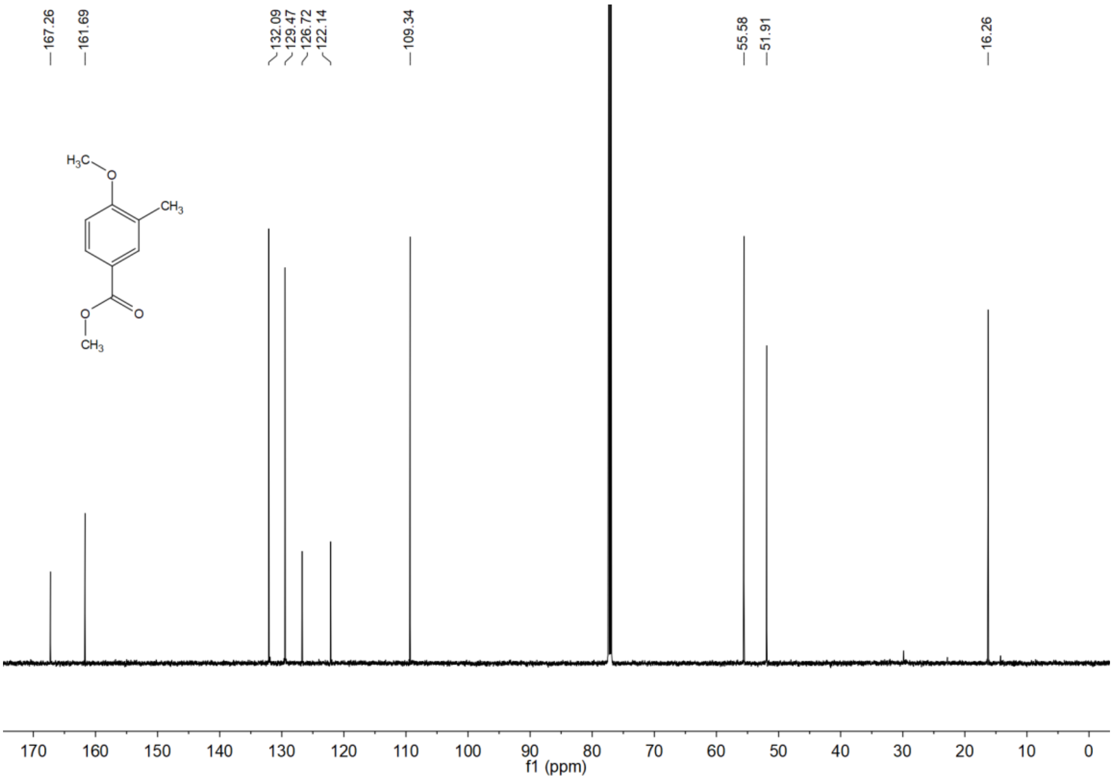

^{1}H NMR (**6c**)

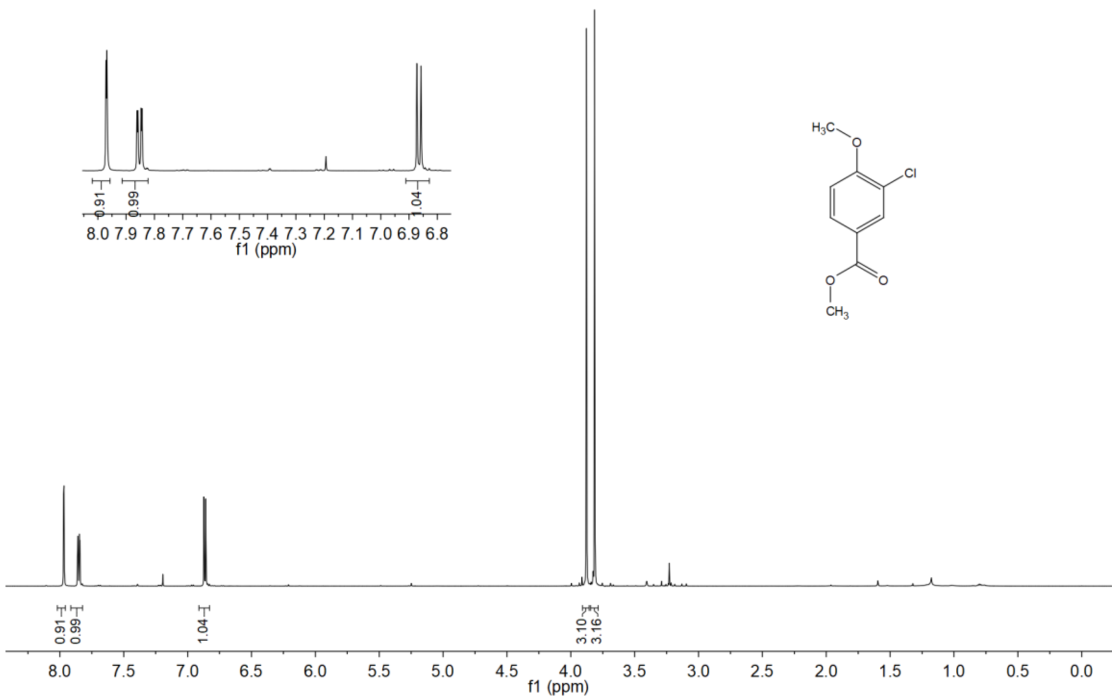

^{13}C NMR (**6c**)

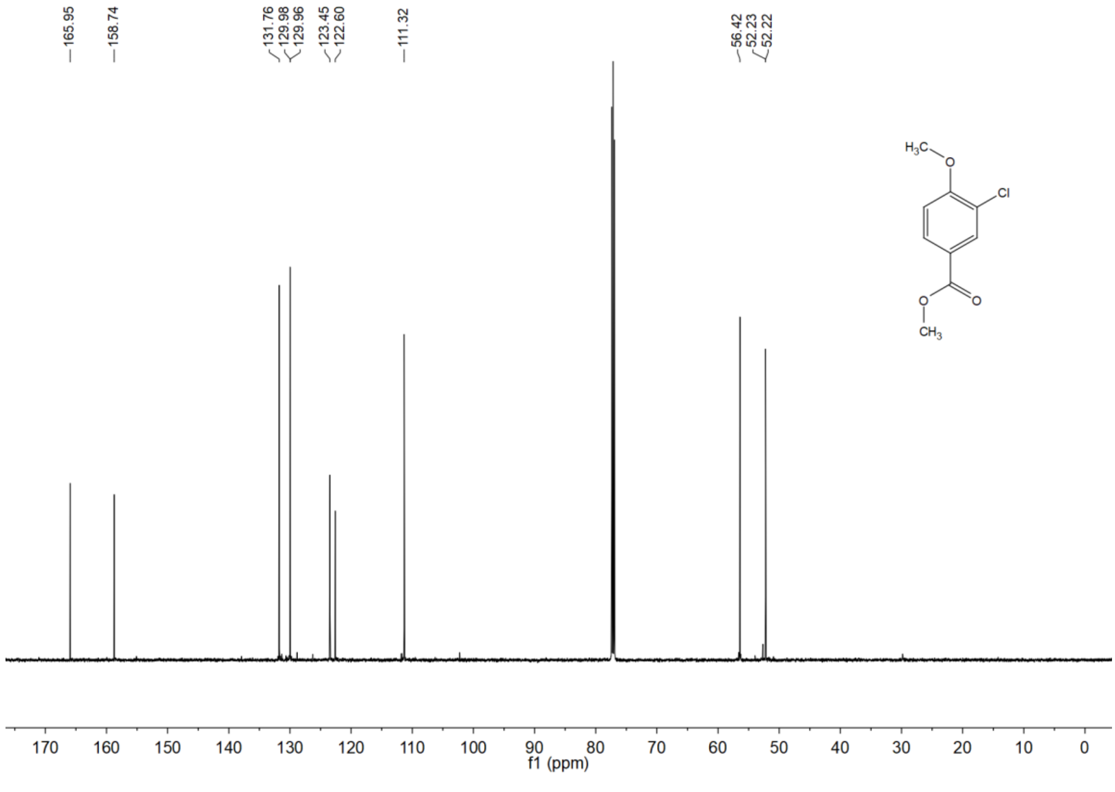

^{1}H NMR (**7c**)

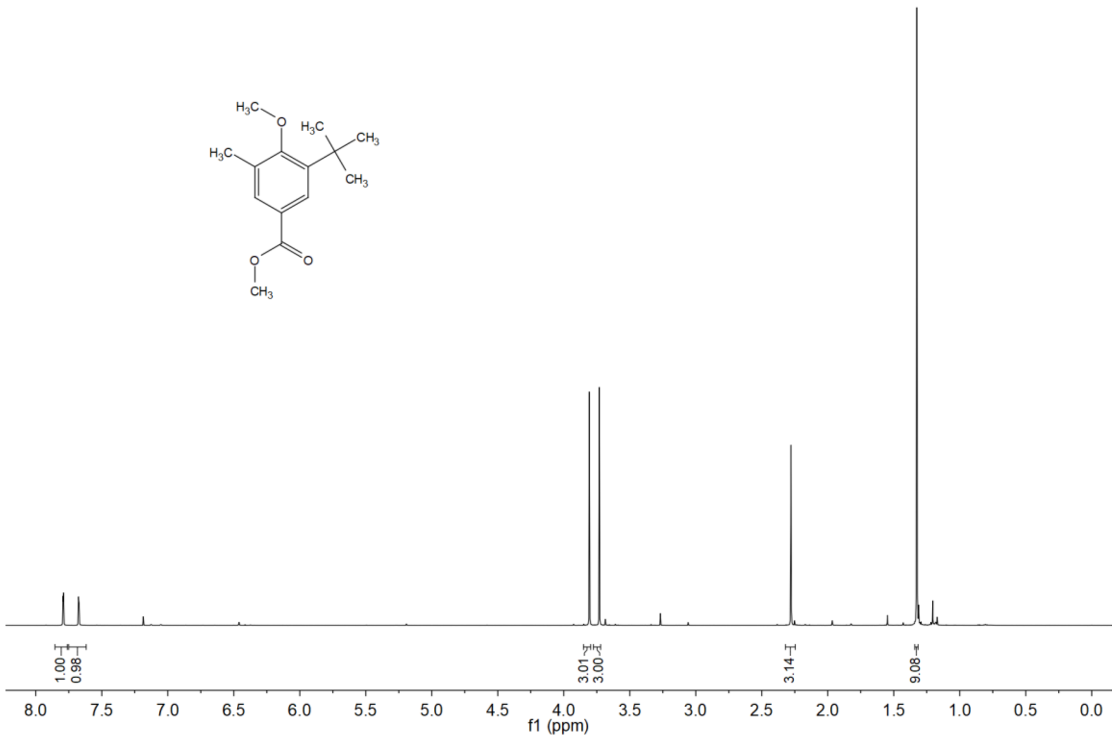

^{13}C NMR (**7c**)

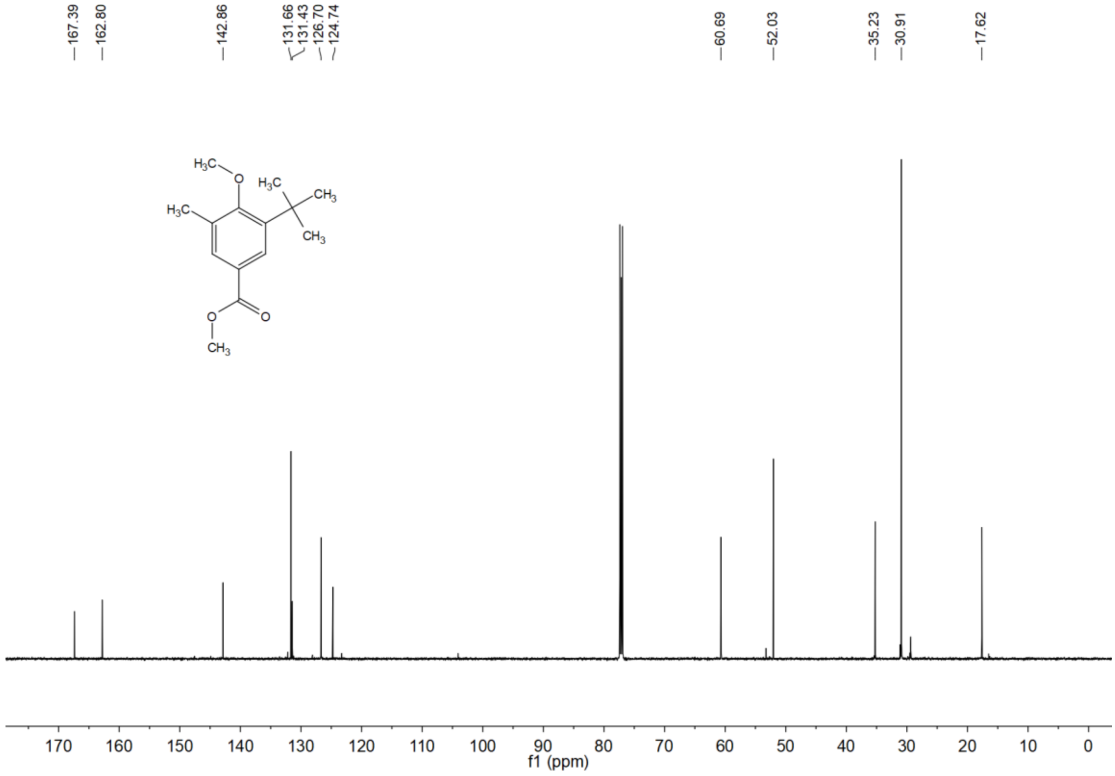

^{1}H NMR (**8c**)

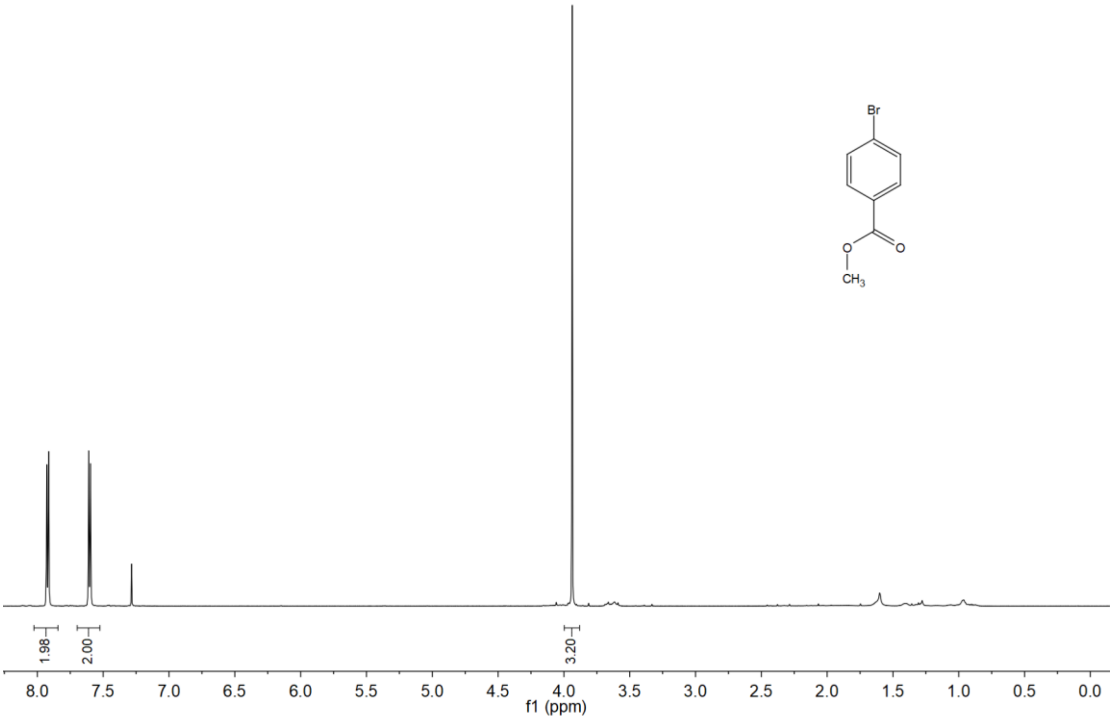

^{13}C NMR (**8c**)

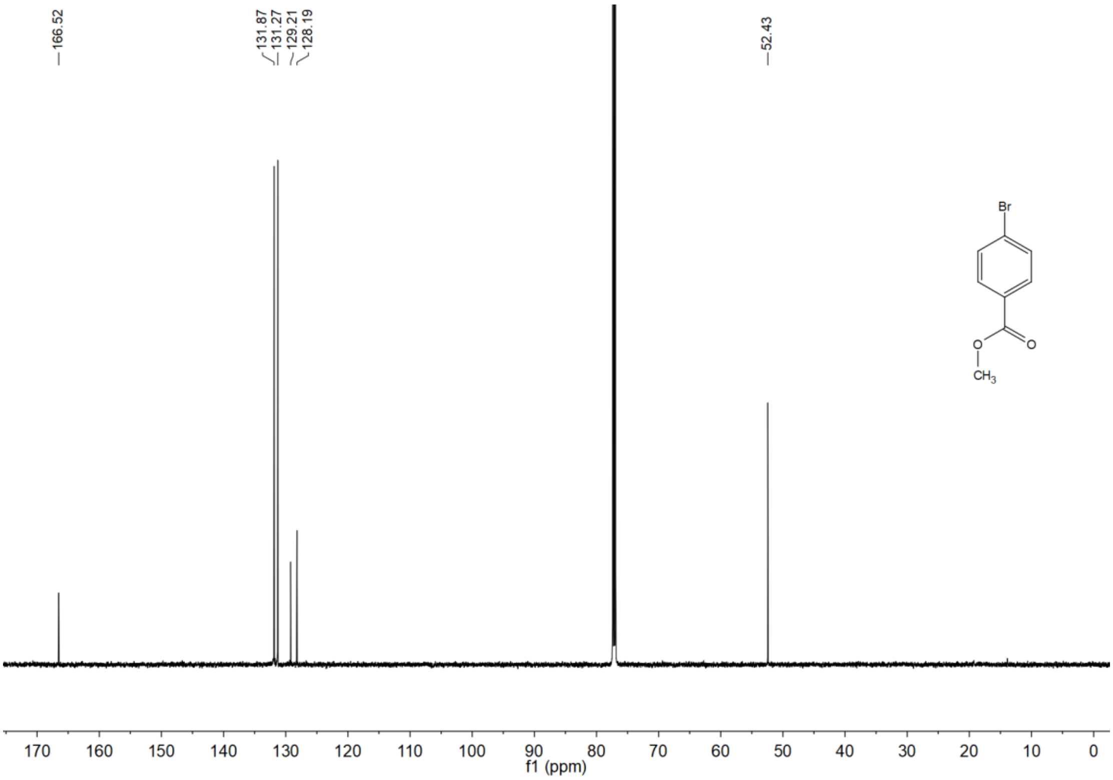

^{1}H NMR (**9c**)

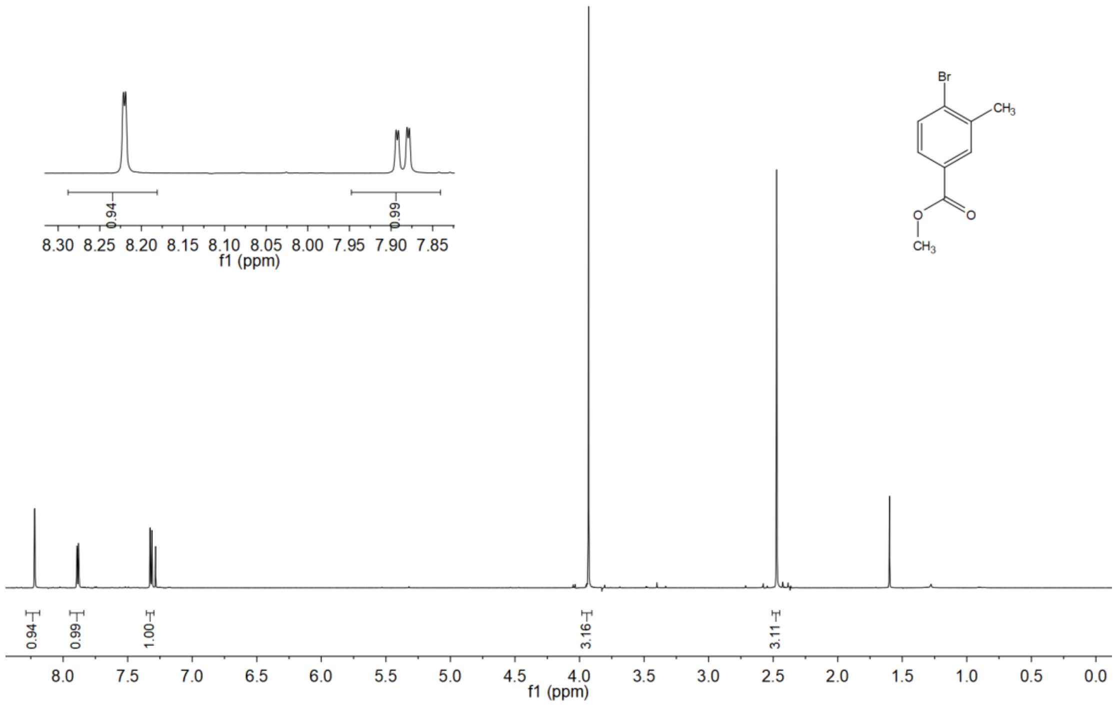

^{13}C NMR (**9c**)

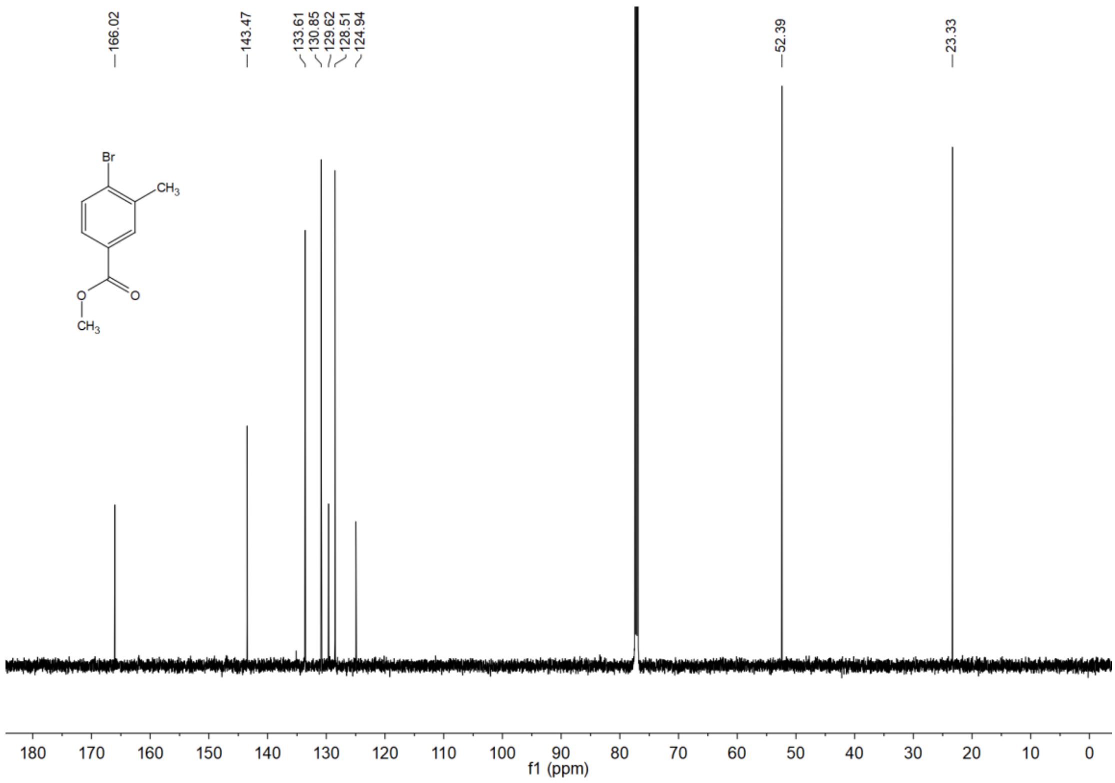

^{1}H NMR (**10c**)

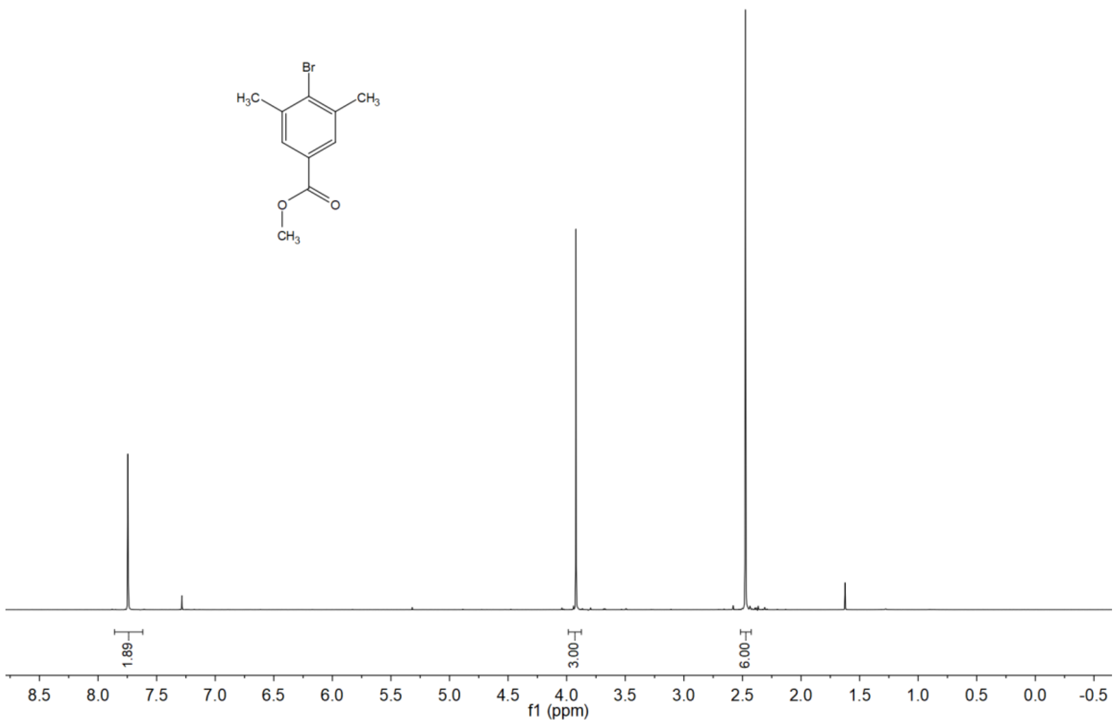

^{13}C NMR (**10c**)

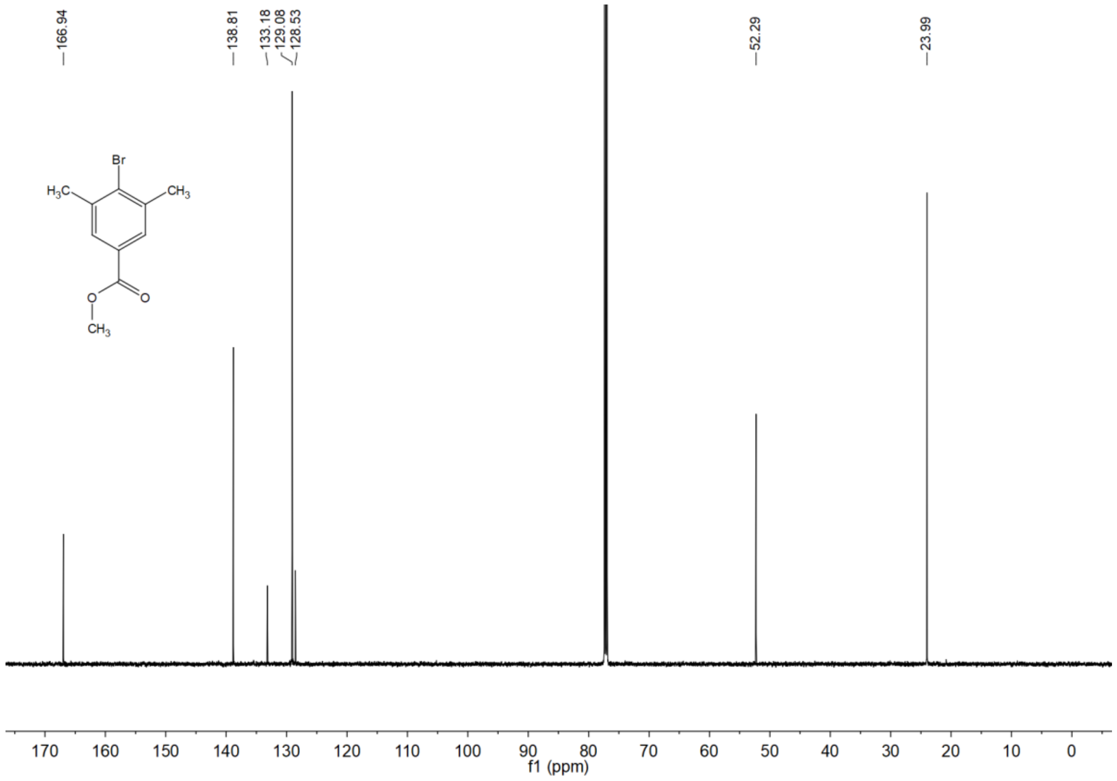

^{1}H NMR (**11c**)

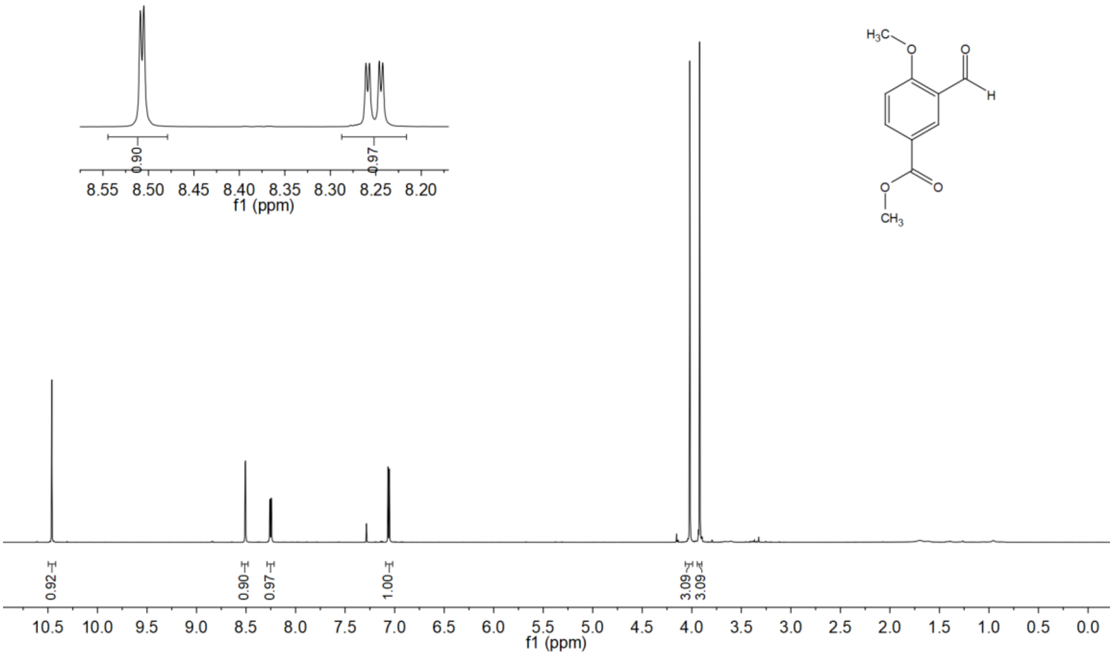

^{13}C NMR (**11c**)

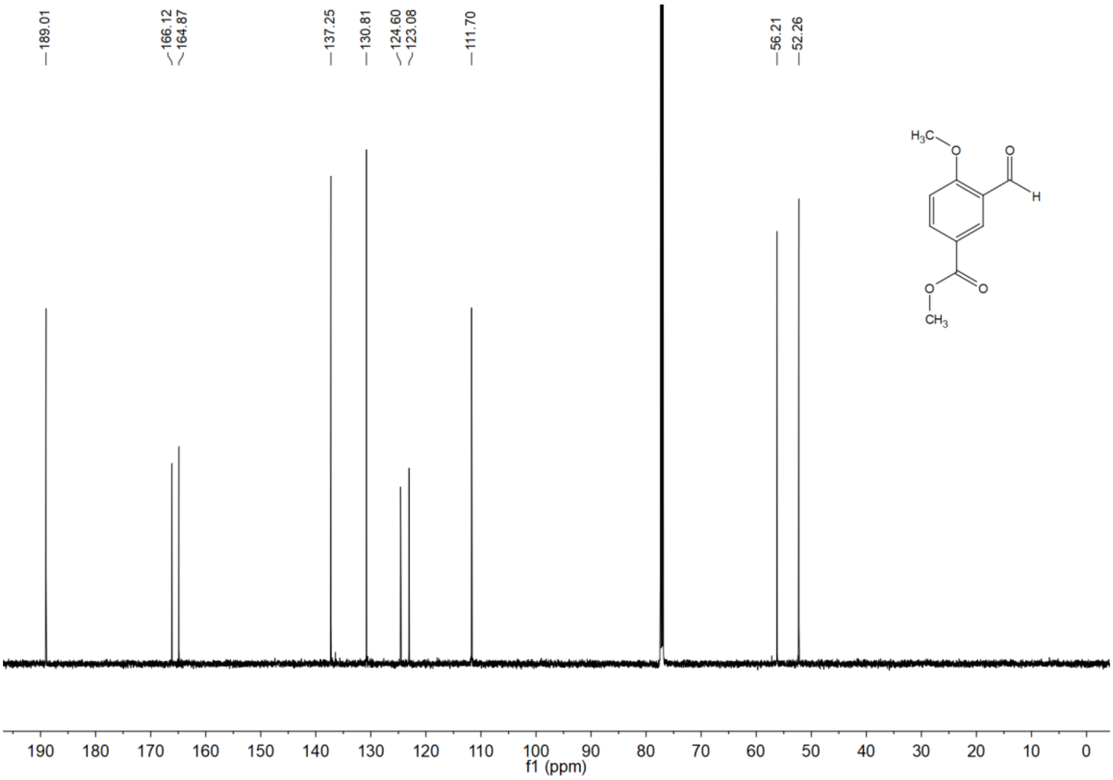

^{1}H NMR (**12c**)

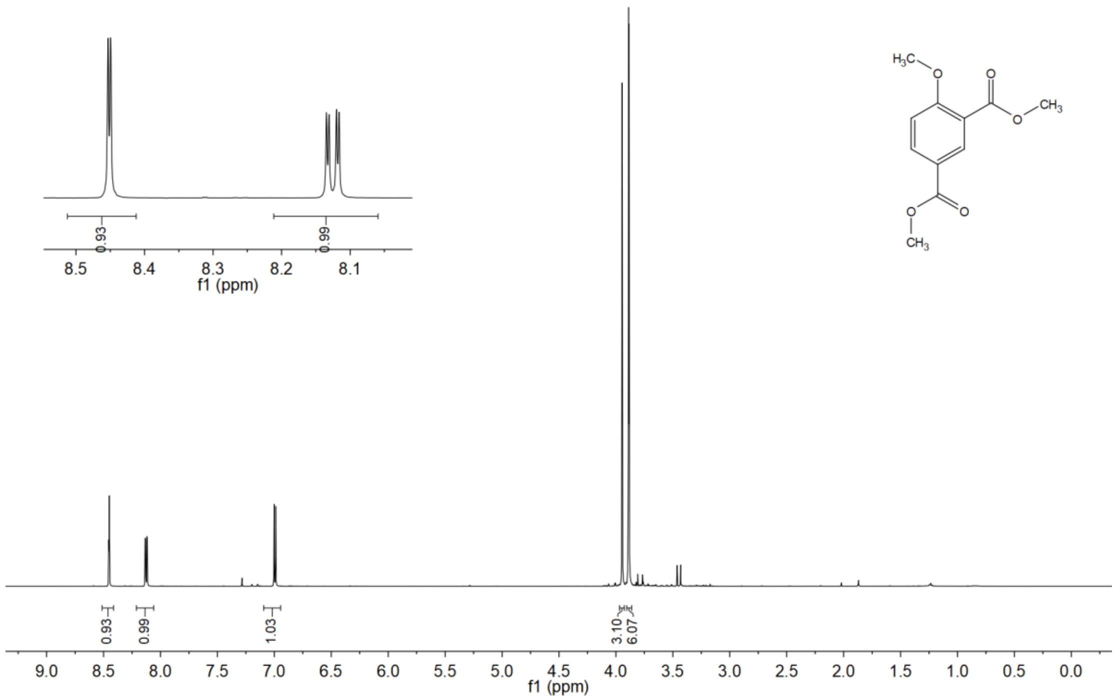

^{13}C NMR (**12c**)

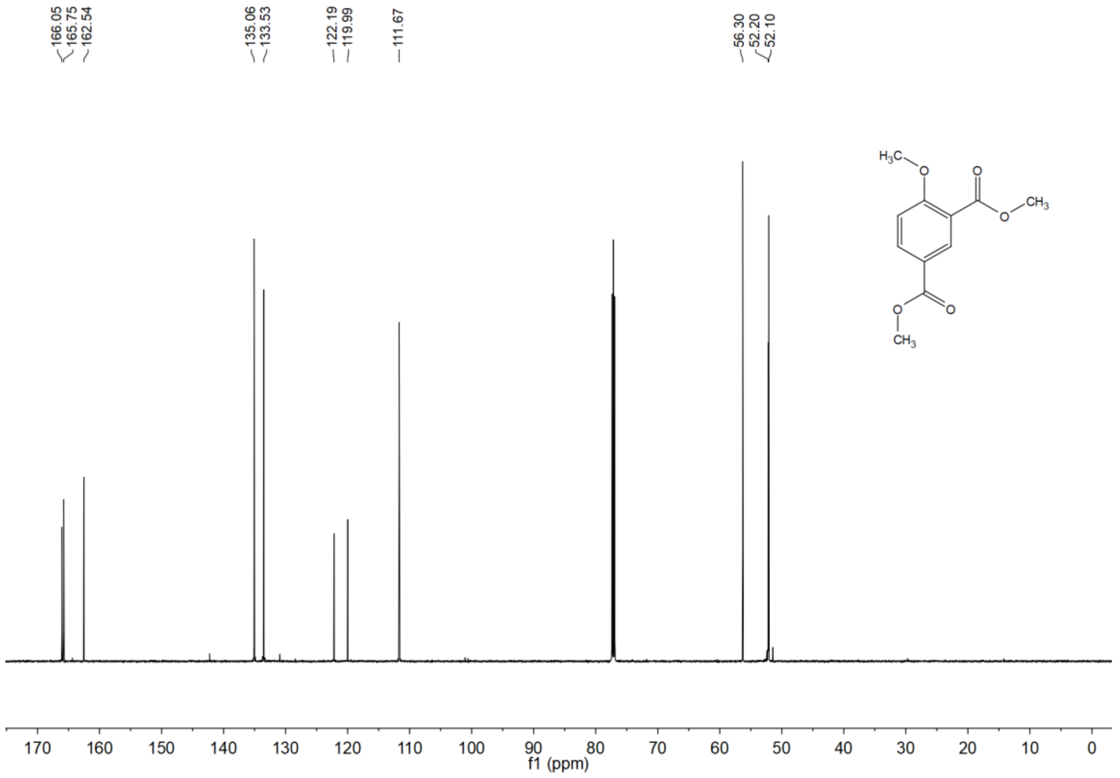

^{1}H NMR (**13c**)

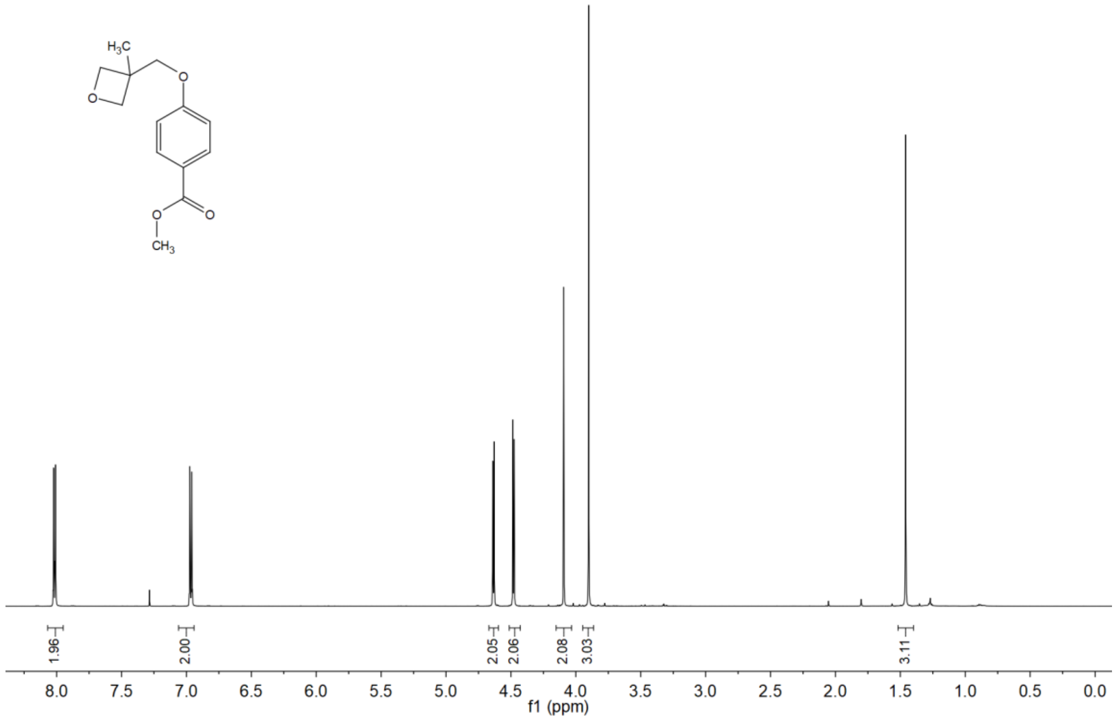

^{13}C NMR (**13c**)

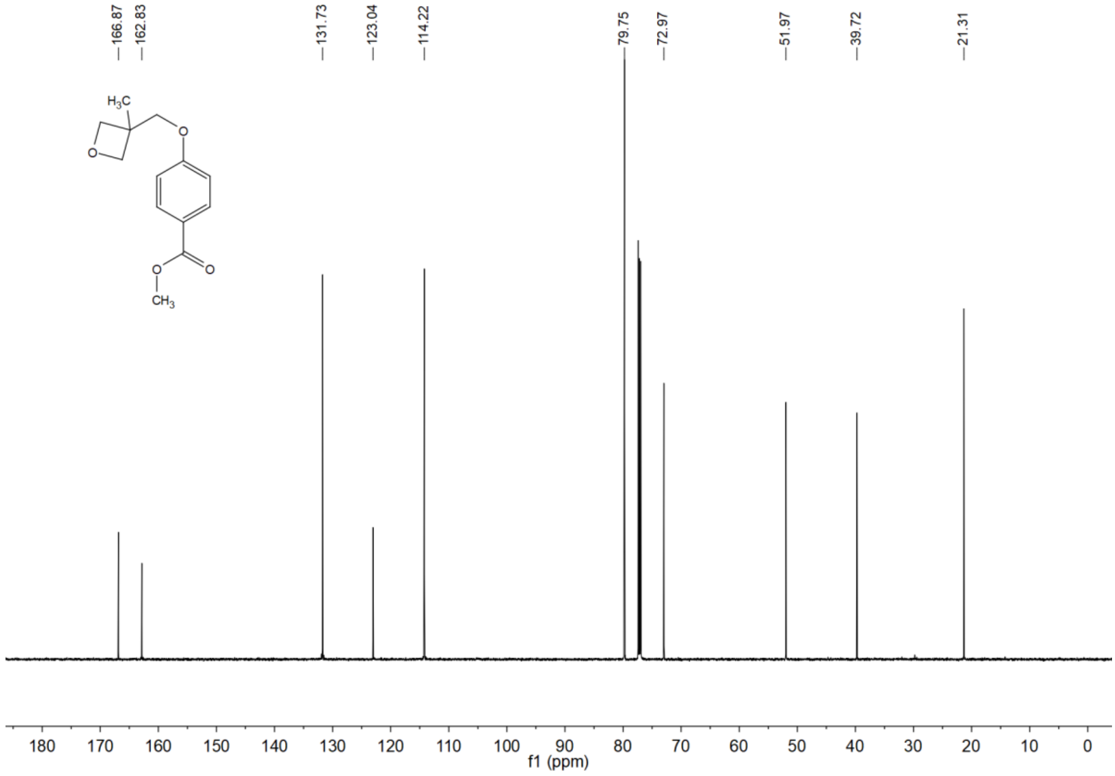

^{1}H NMR (**14c**)

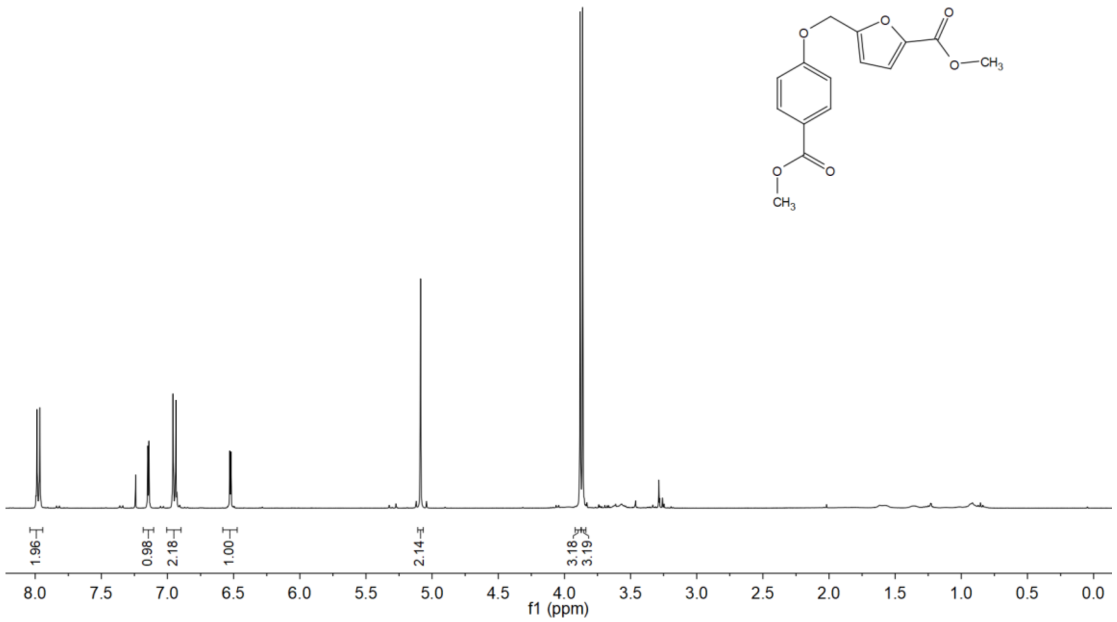

^{13}C NMR (**14c**)

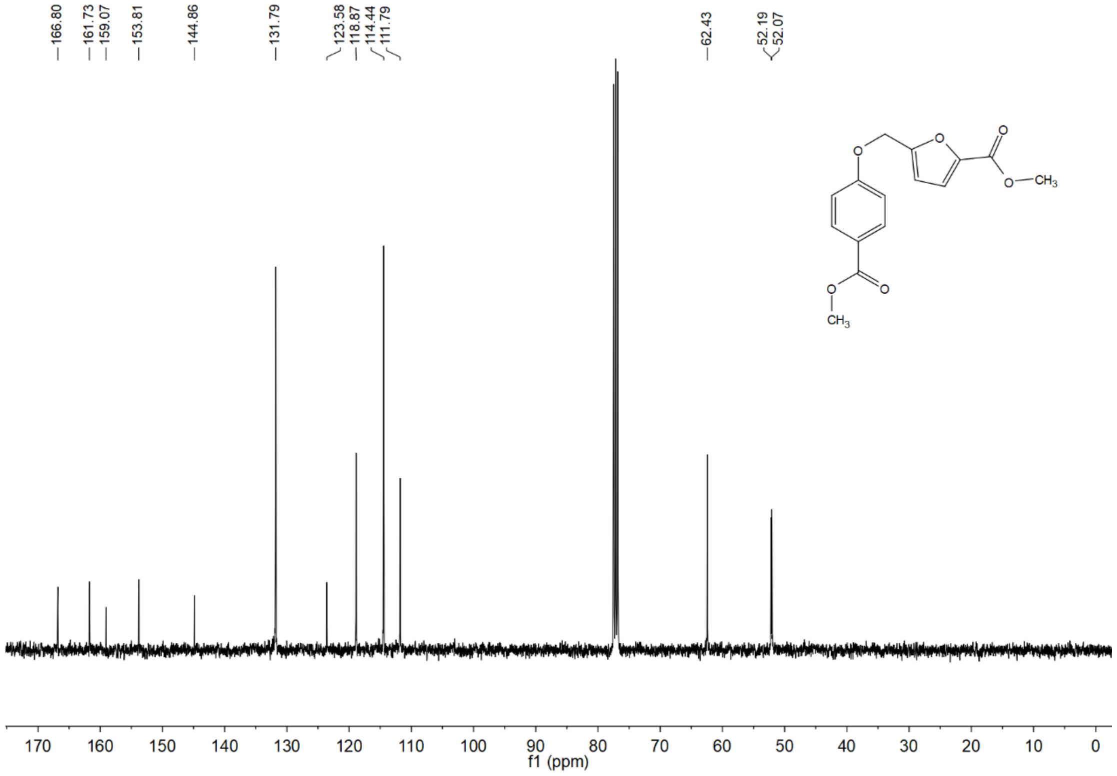

^{1}H NMR (**15c**)

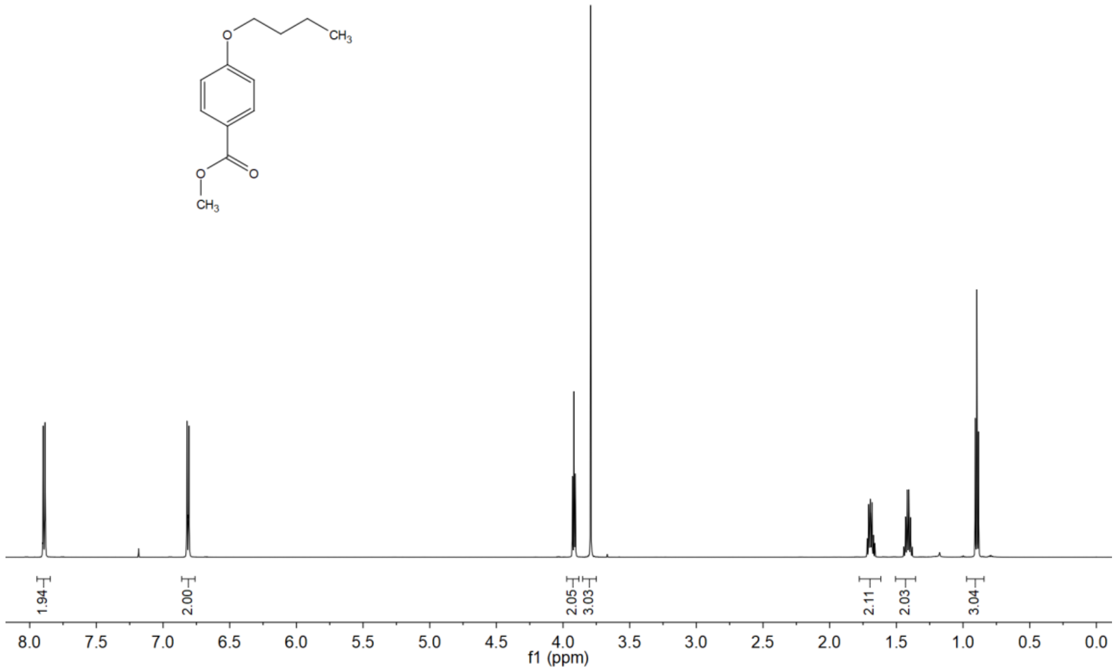

^{13}C NMR (**15c**)

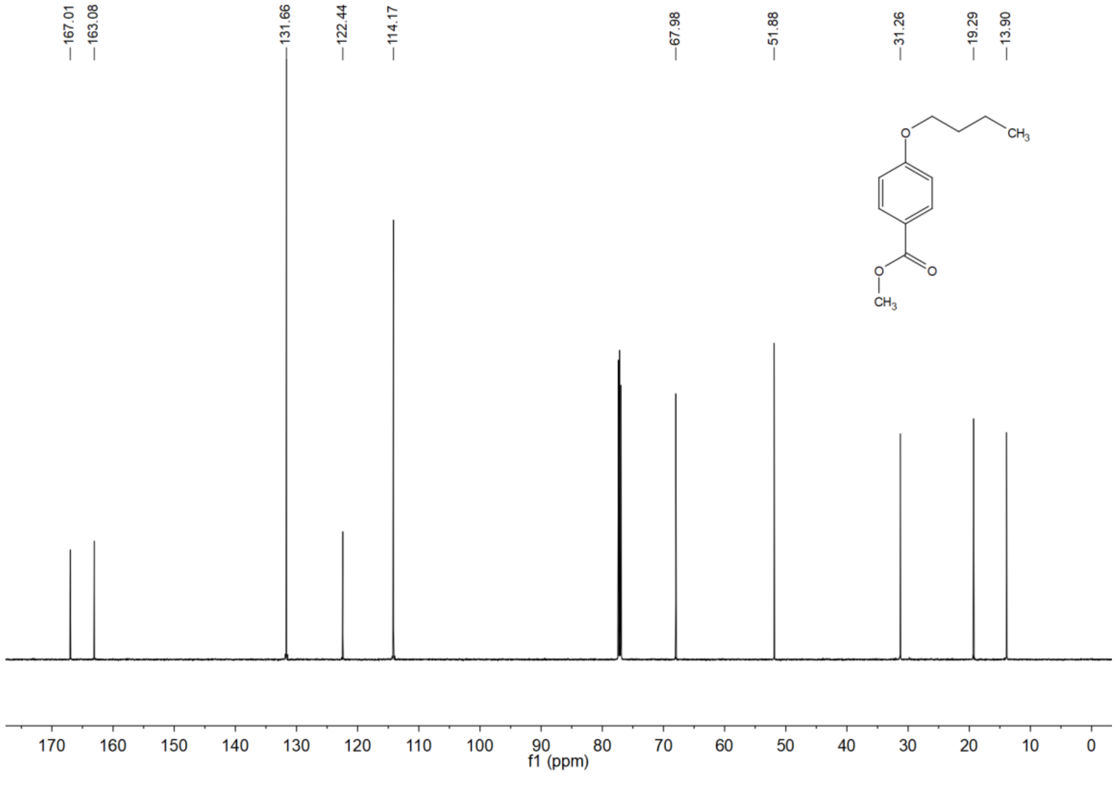

^{1}H NMR (**16c**)

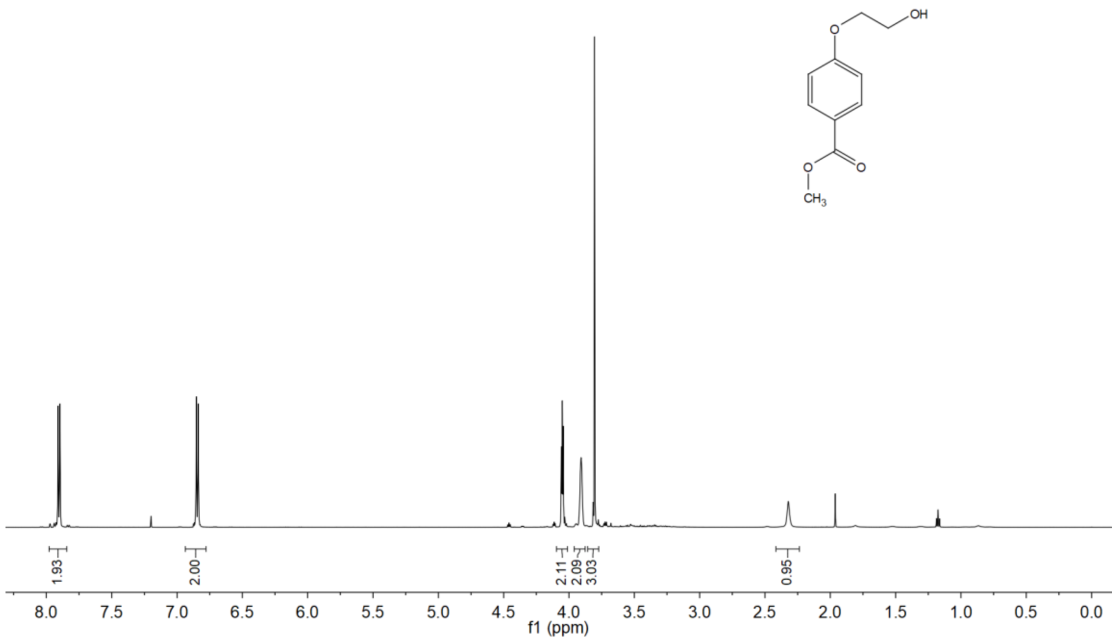

^{13}C NMR (**16c**)

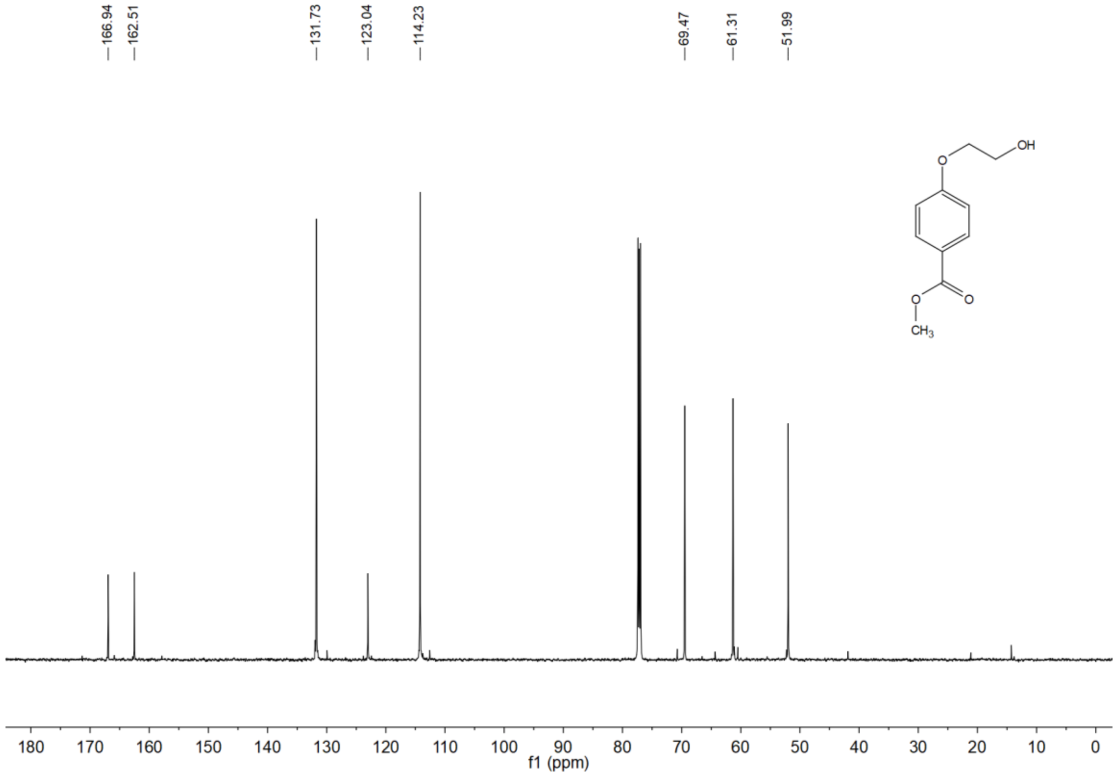

^{1}H NMR (**17c**)

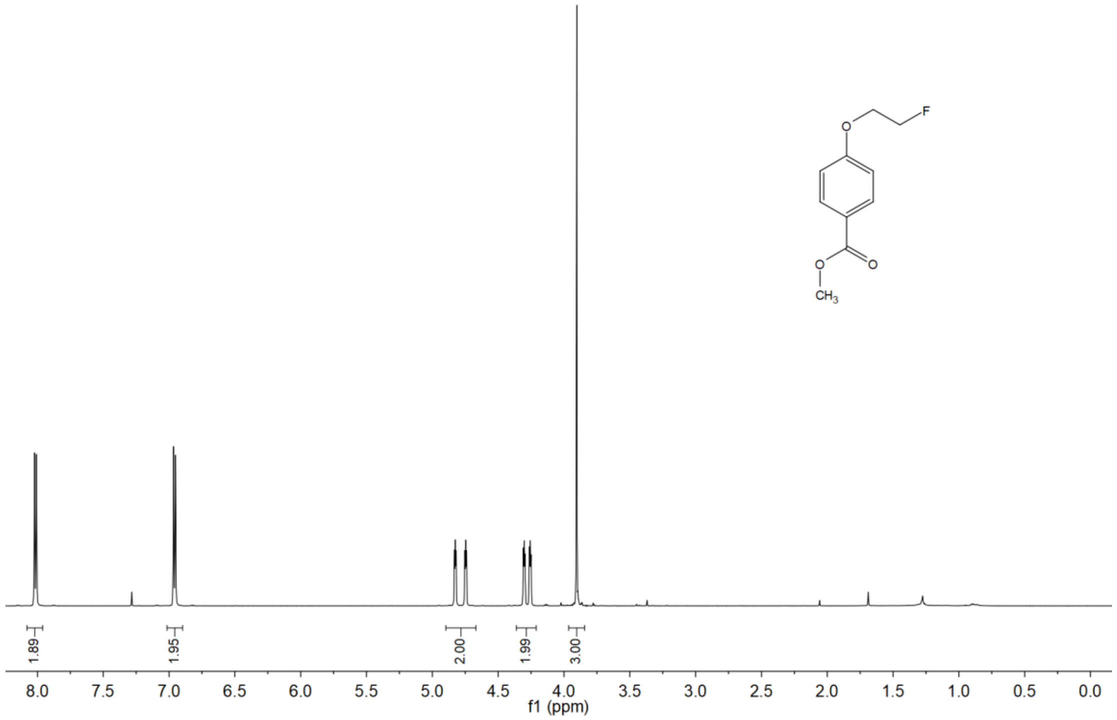

^{13}C NMR (**17c**)

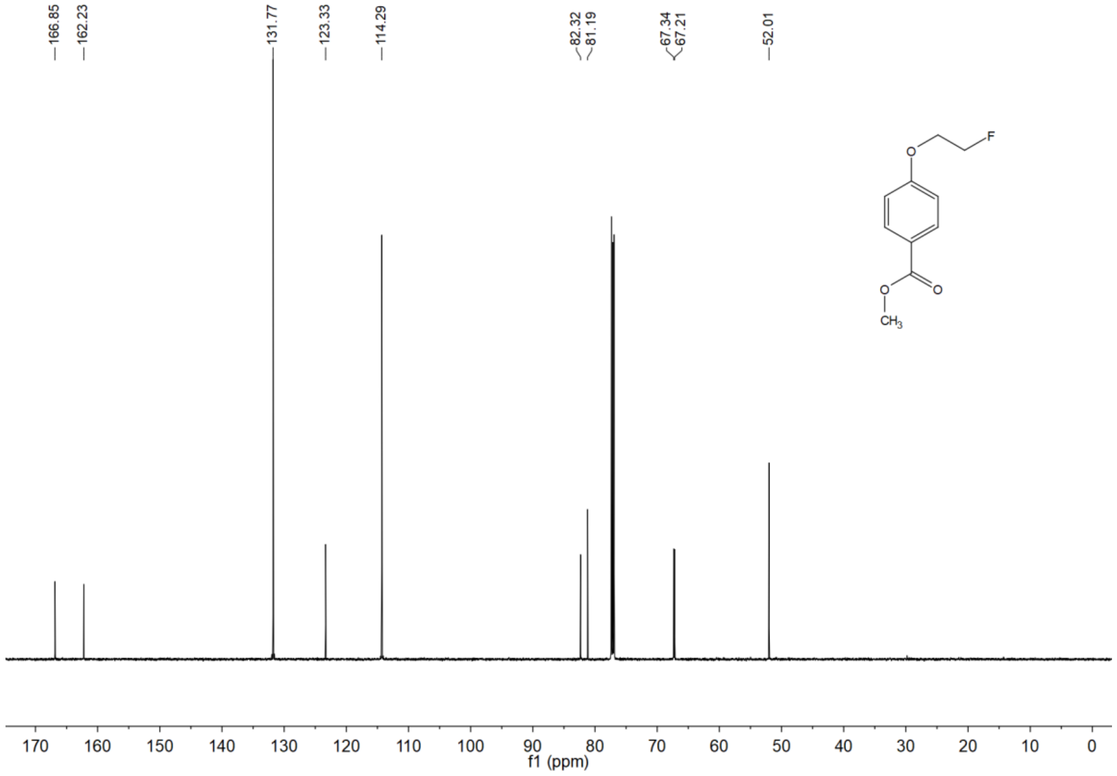

^{1}H NMR (**18c**)

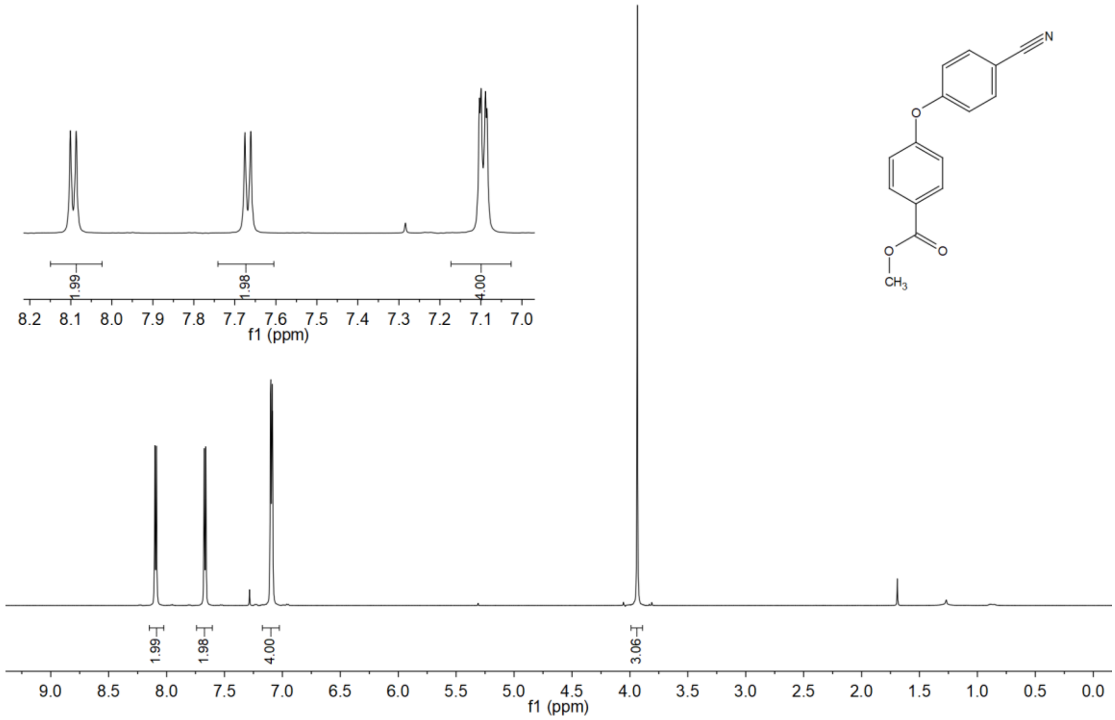

^{13}C NMR (**18c**)

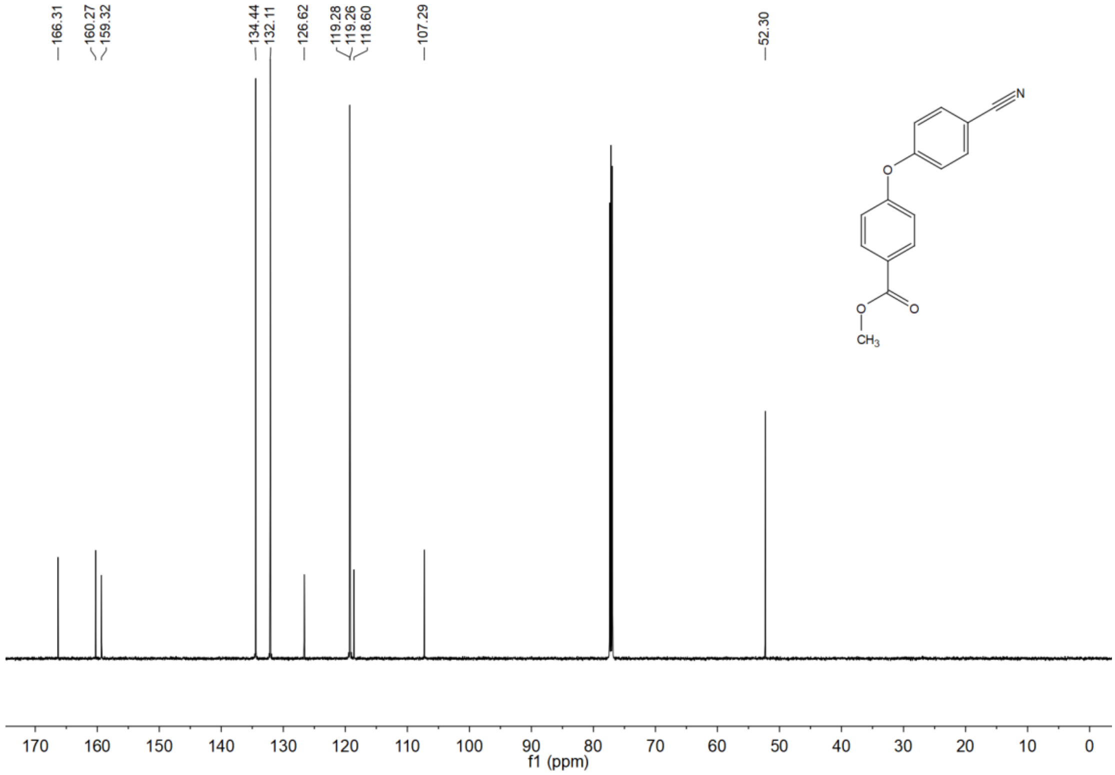

^{1}H NMR (**19c**)

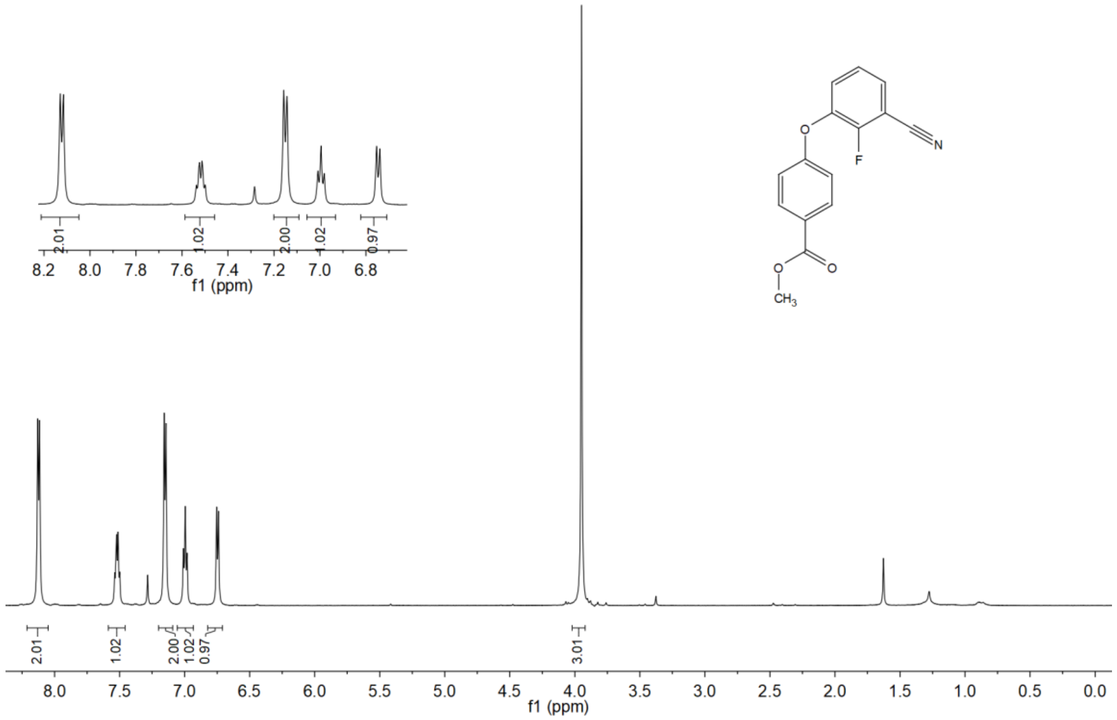

^{13}C NMR (**19c**)

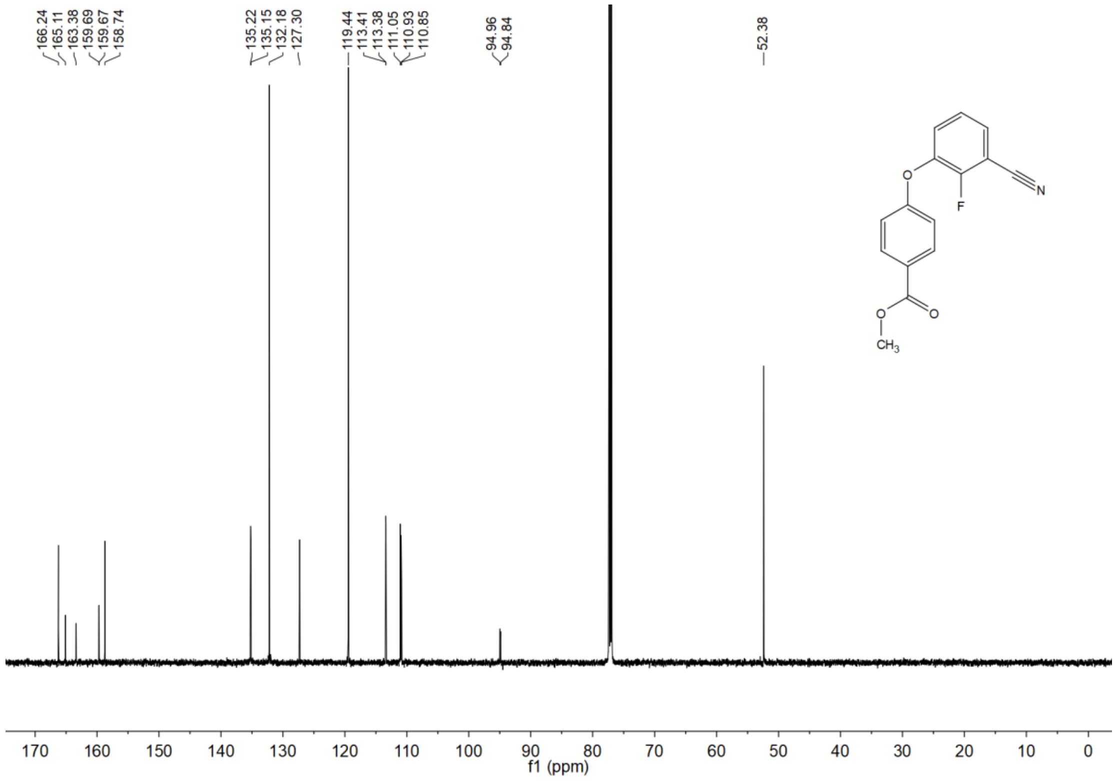

^{1}H NMR (**20c**)

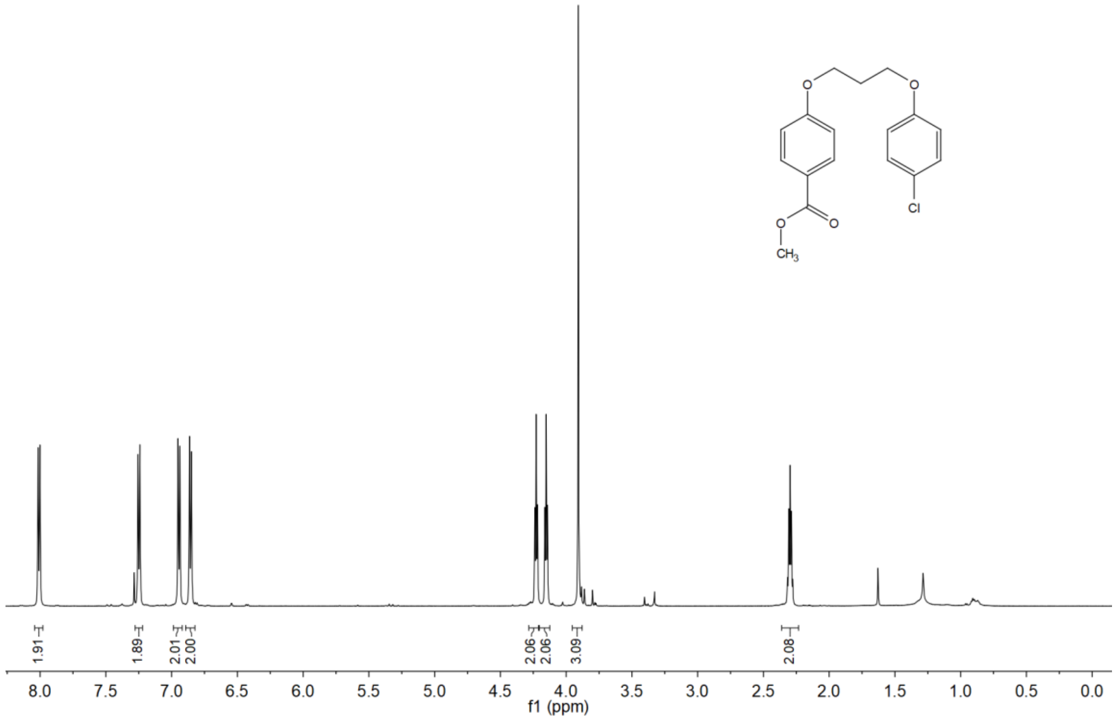

^{13}C NMR (**20c**)

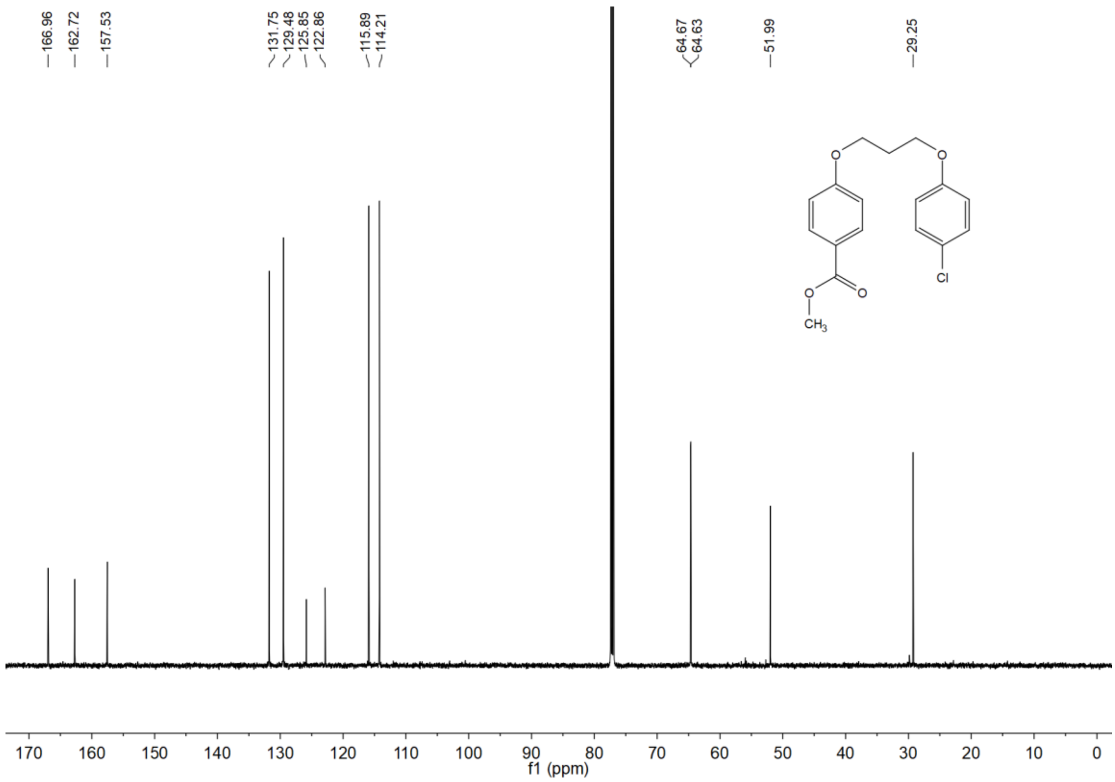

^{1}H NMR (**21c**)

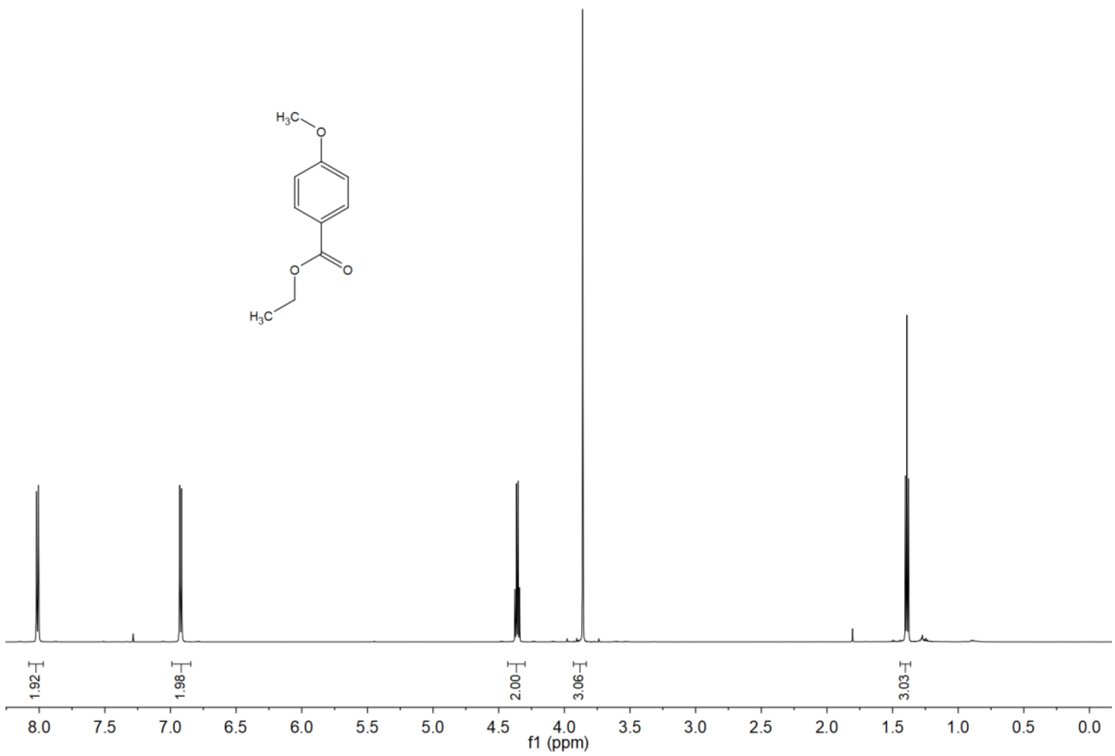

^{13}C NMR (**21c**)

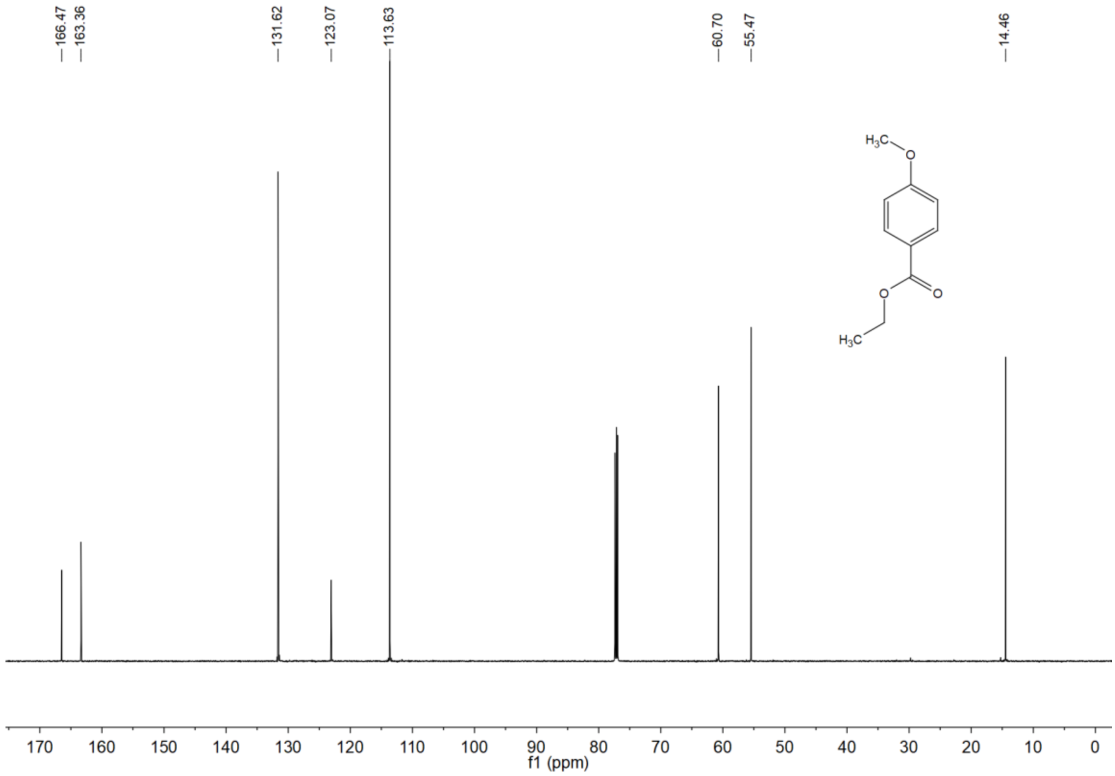

^{1}H NMR (**22c**)

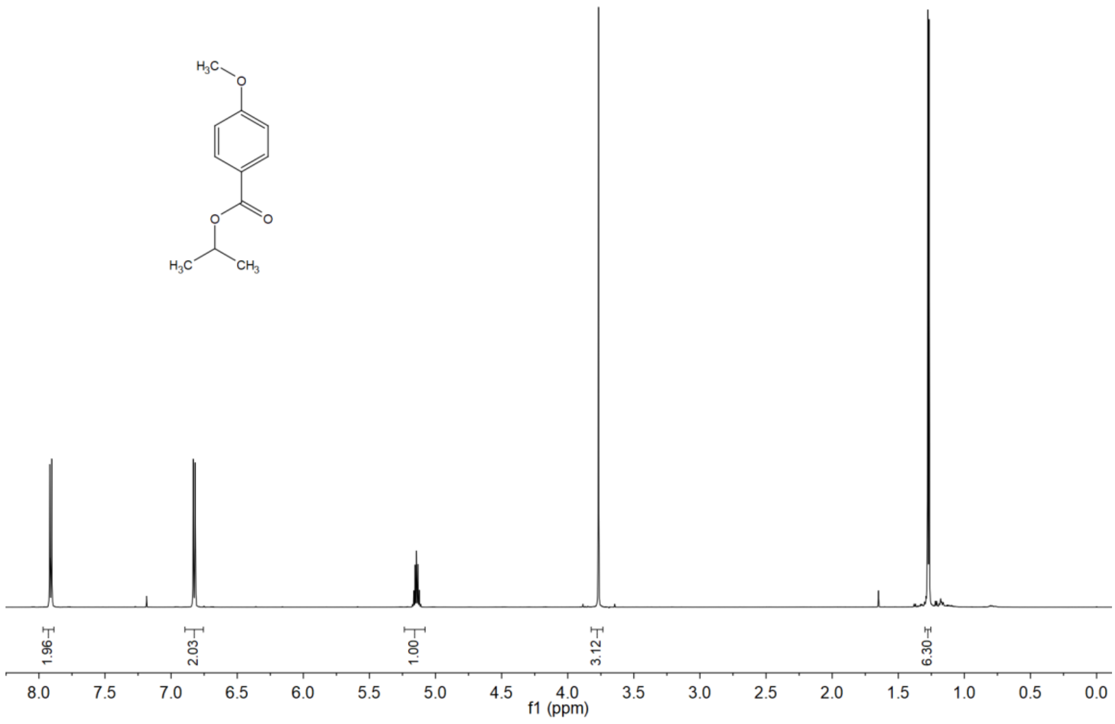

^{13}C NMR (**22c**)

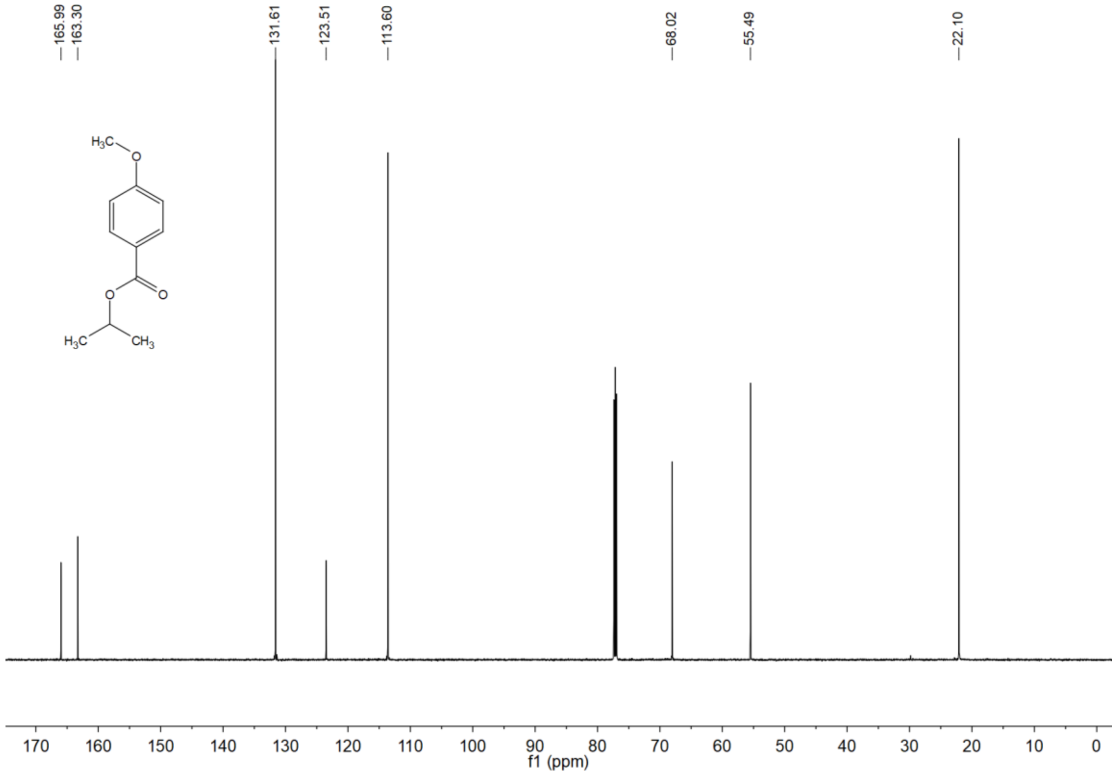

^{1}H NMR (**23c**)

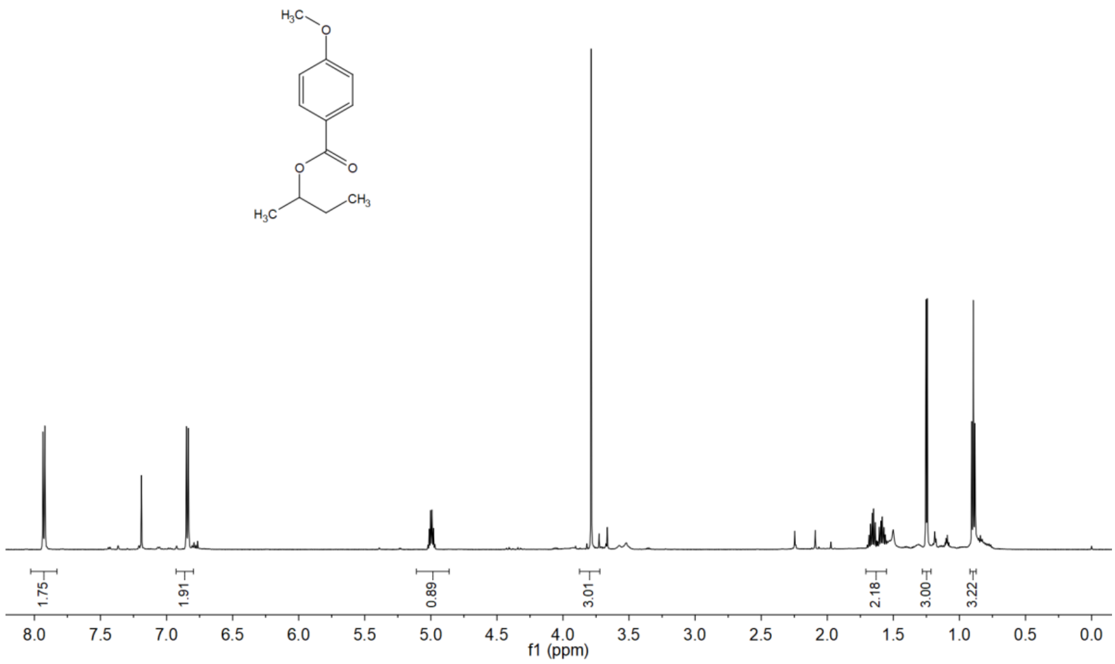

^{13}C NMR (**23c**)

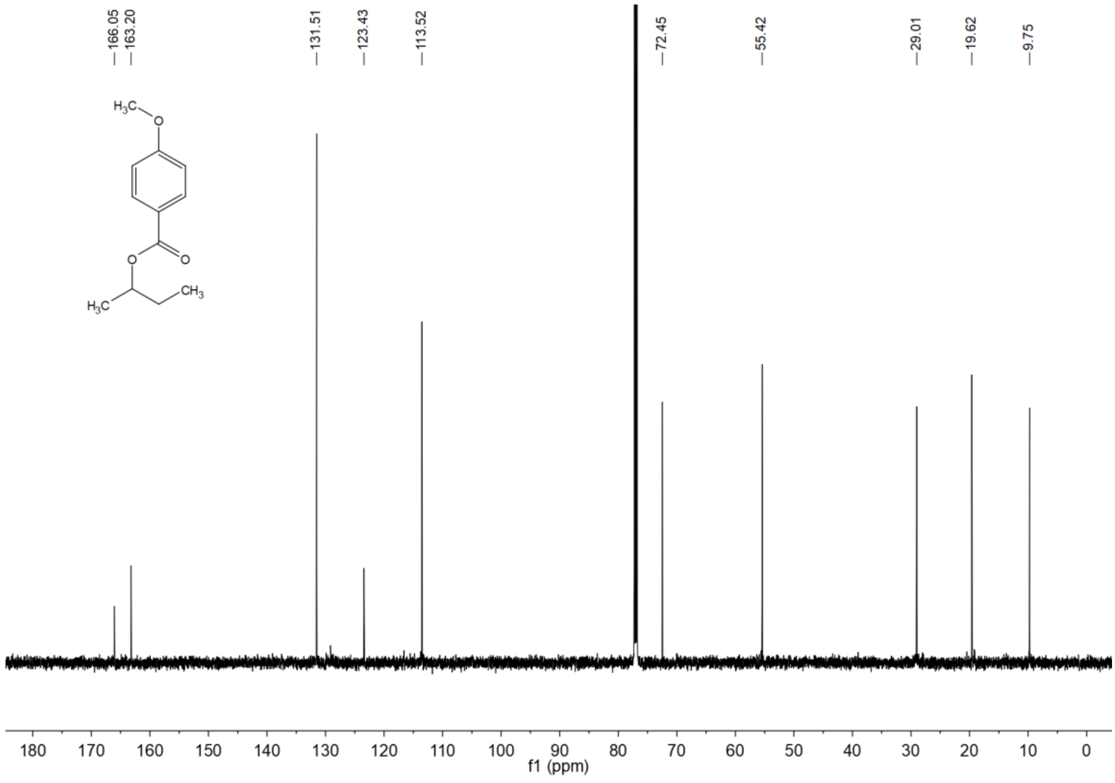

^{1}H NMR (**24c**)

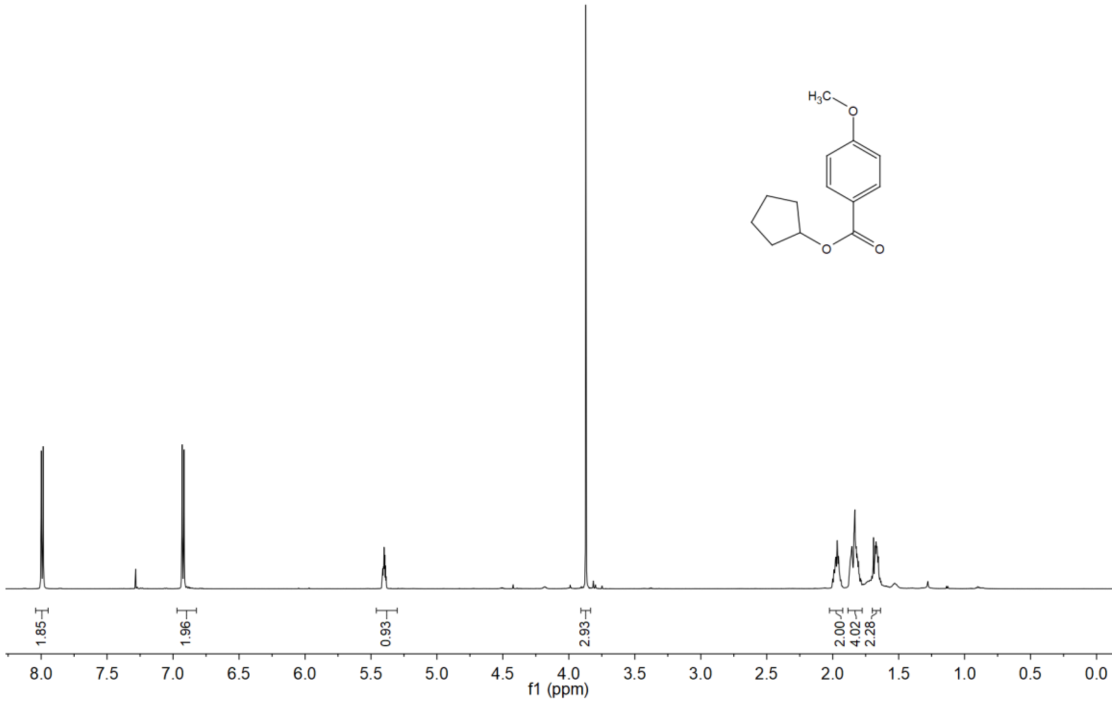

^{13}C NMR (**24c**)

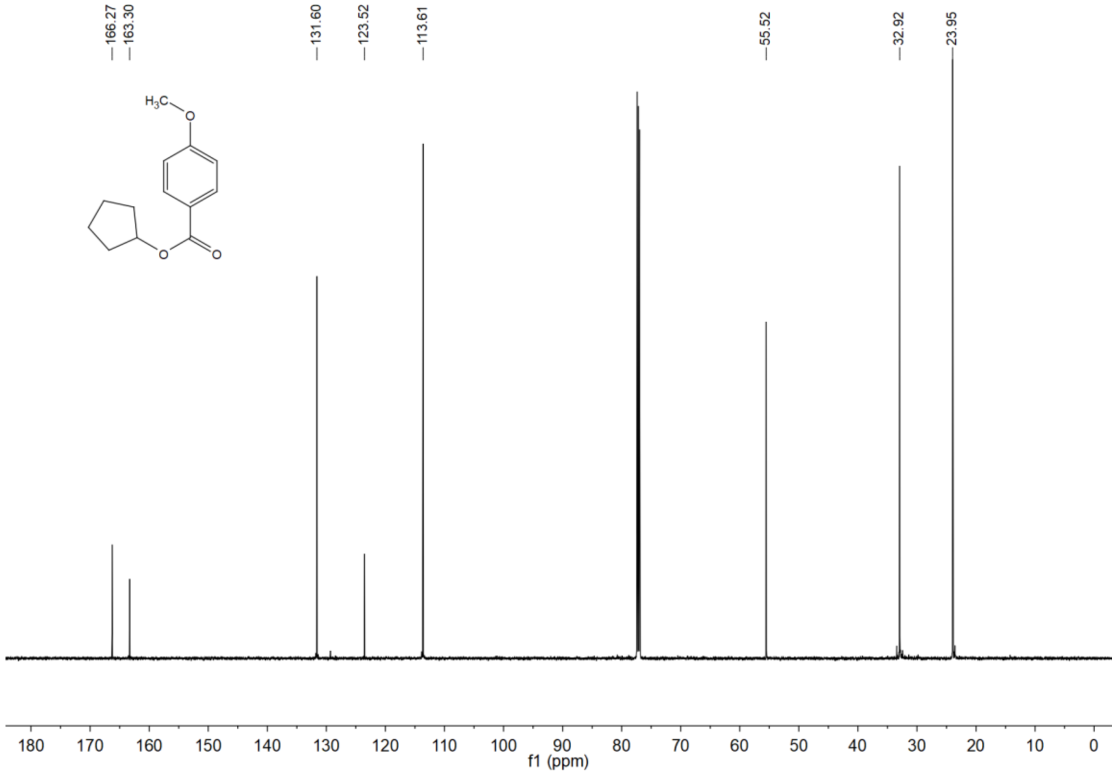

^{1}H NMR (**25c**)

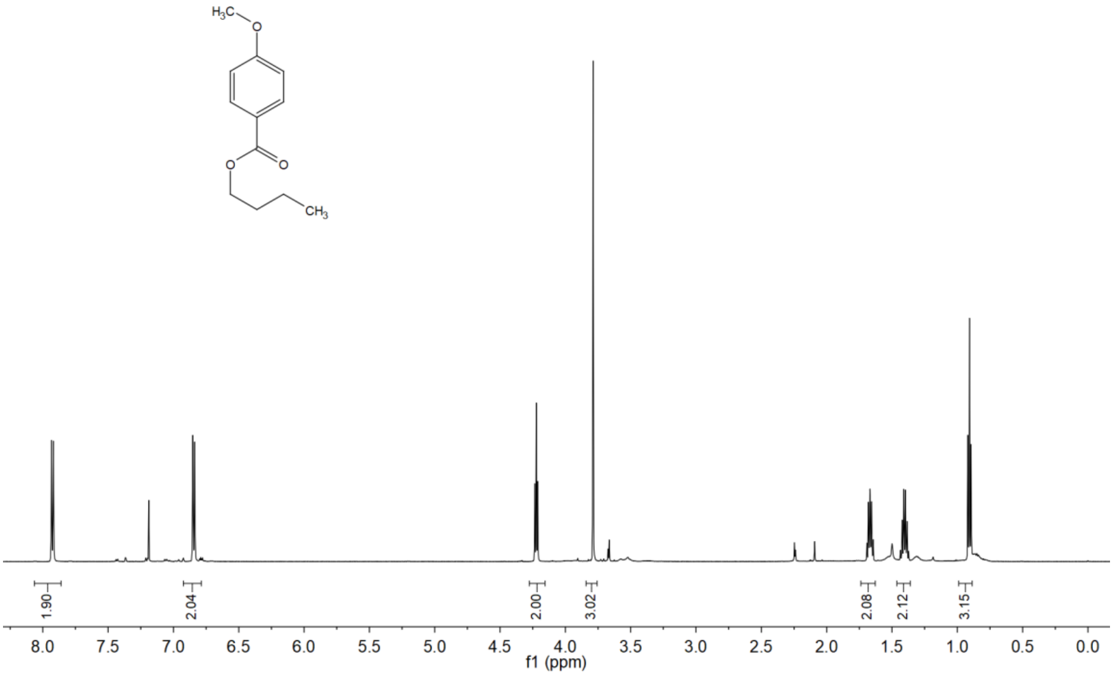

^{13}C NMR (**25c**)

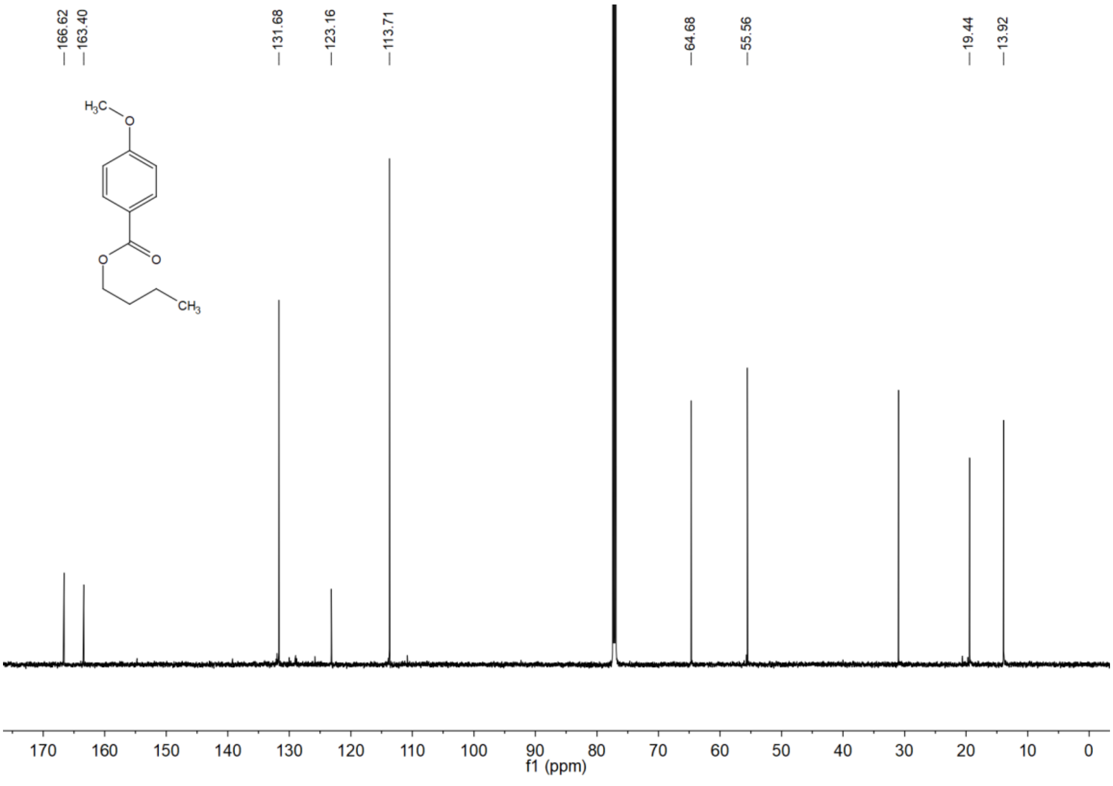

^{1}H NMR (**26c**)

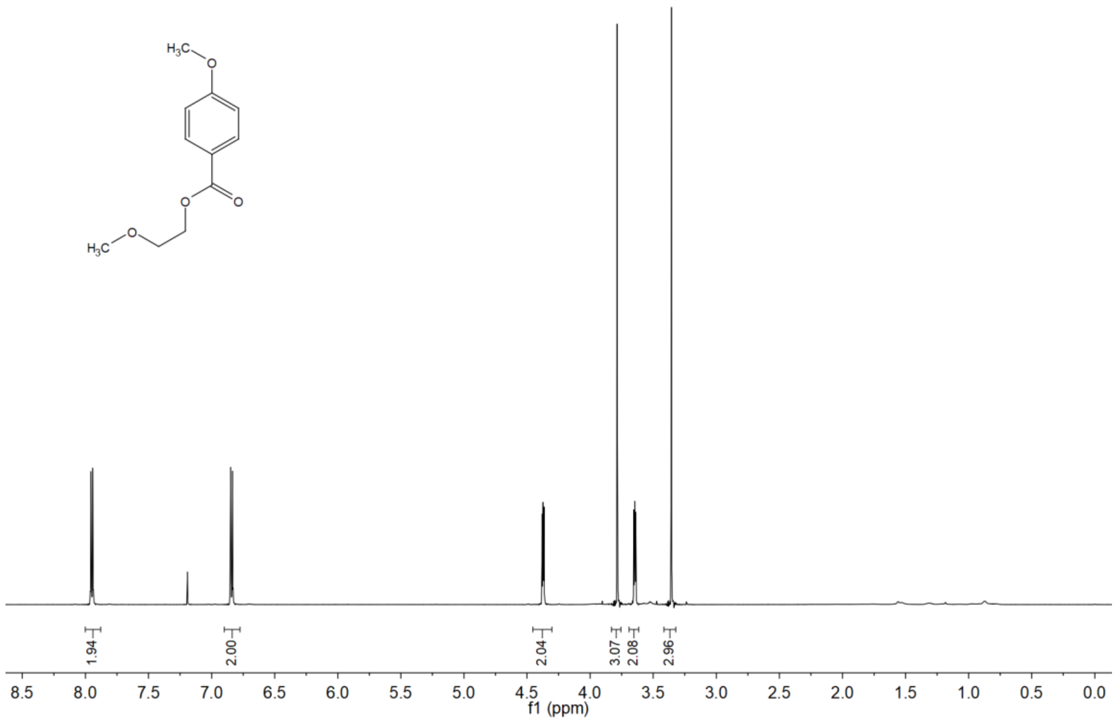

^{13}C NMR (**26c**)

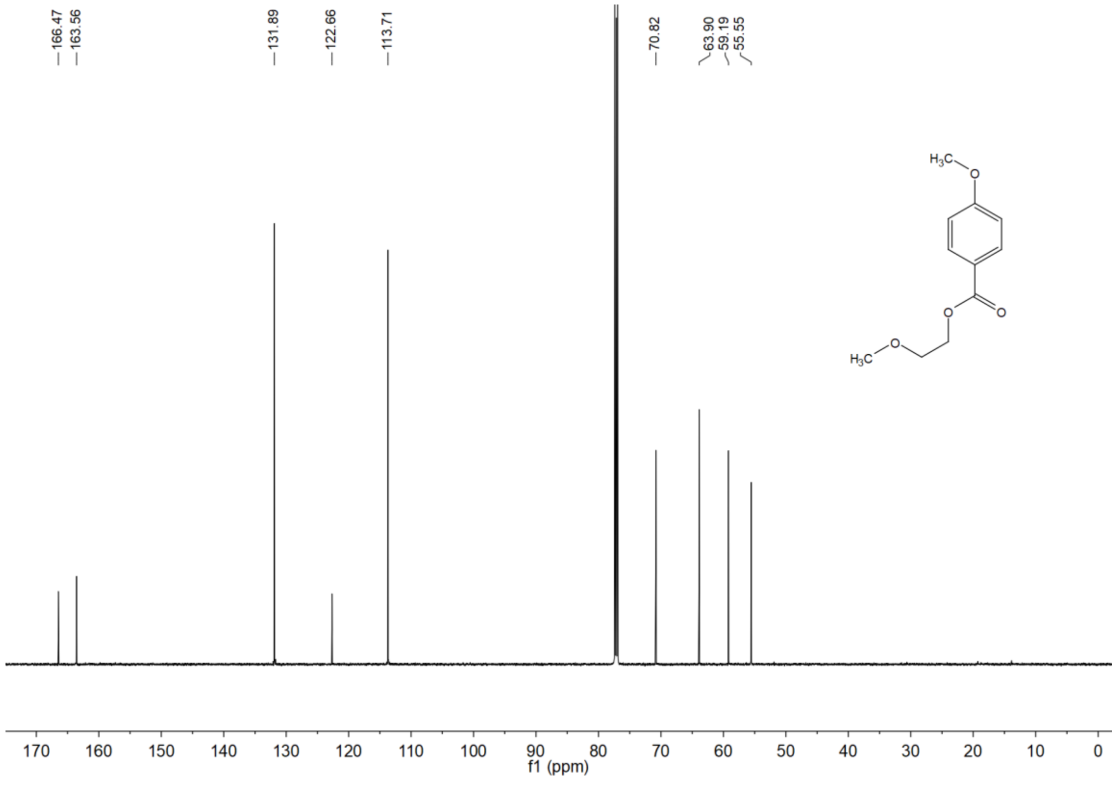

^{1}H NMR (**27c**)

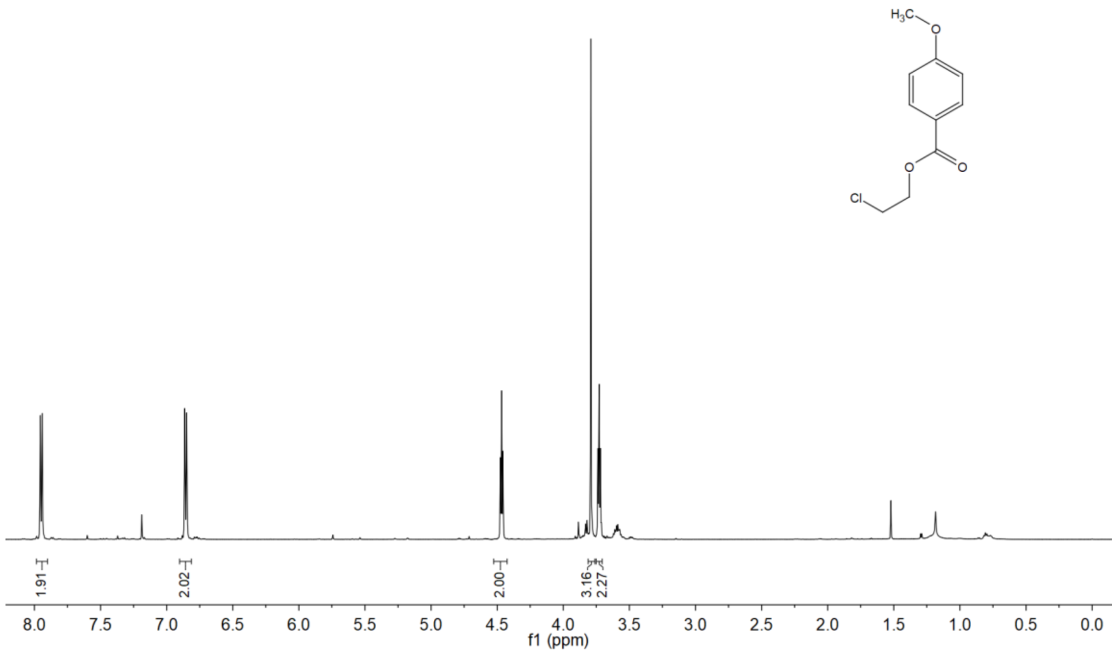

^{13}C NMR (**27c**)

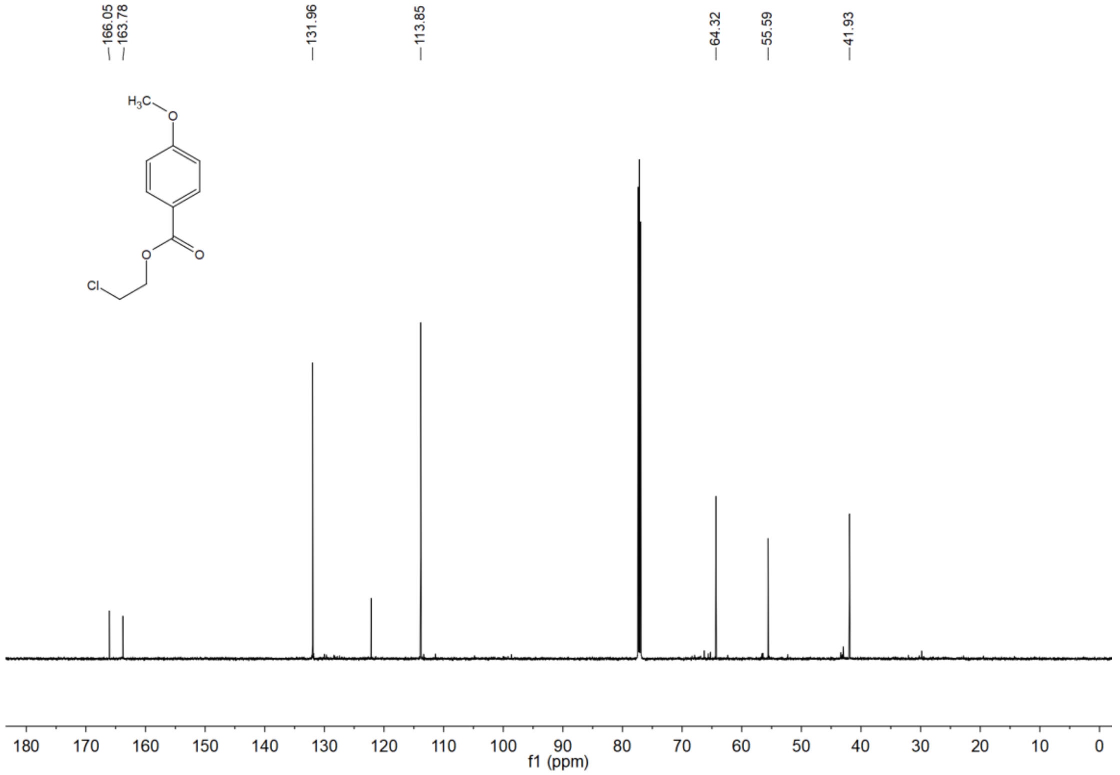

^{1}H NMR (**28c**)

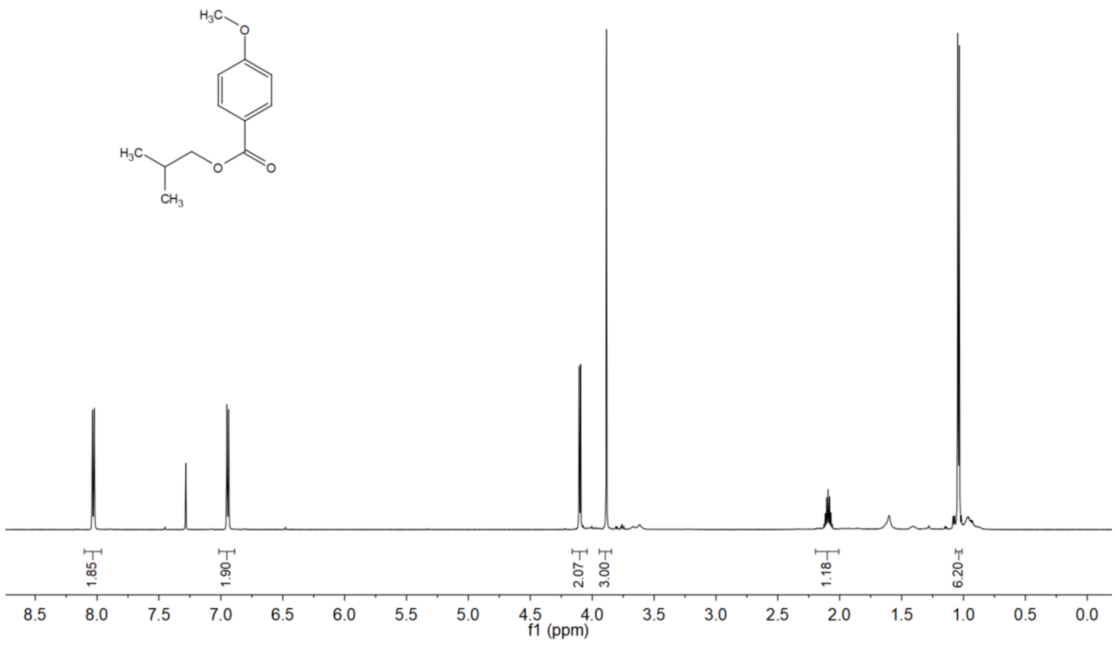

^{13}C NMR (**28c**)

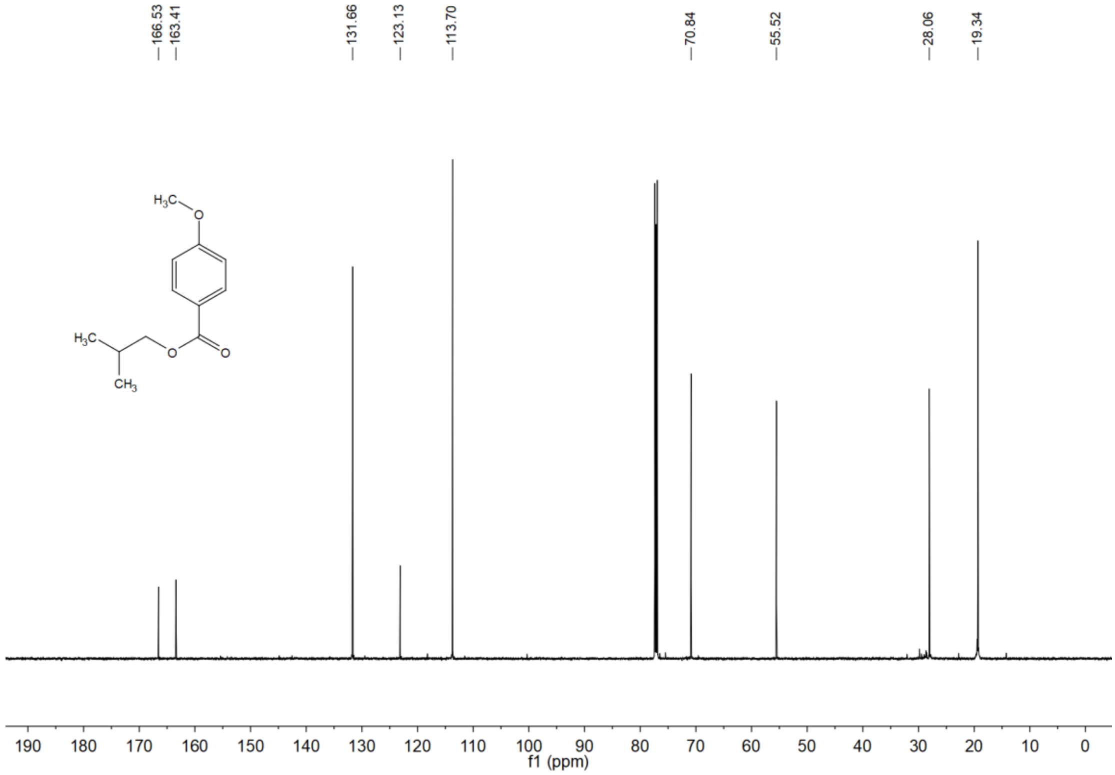

^{1}H NMR (**29c**)

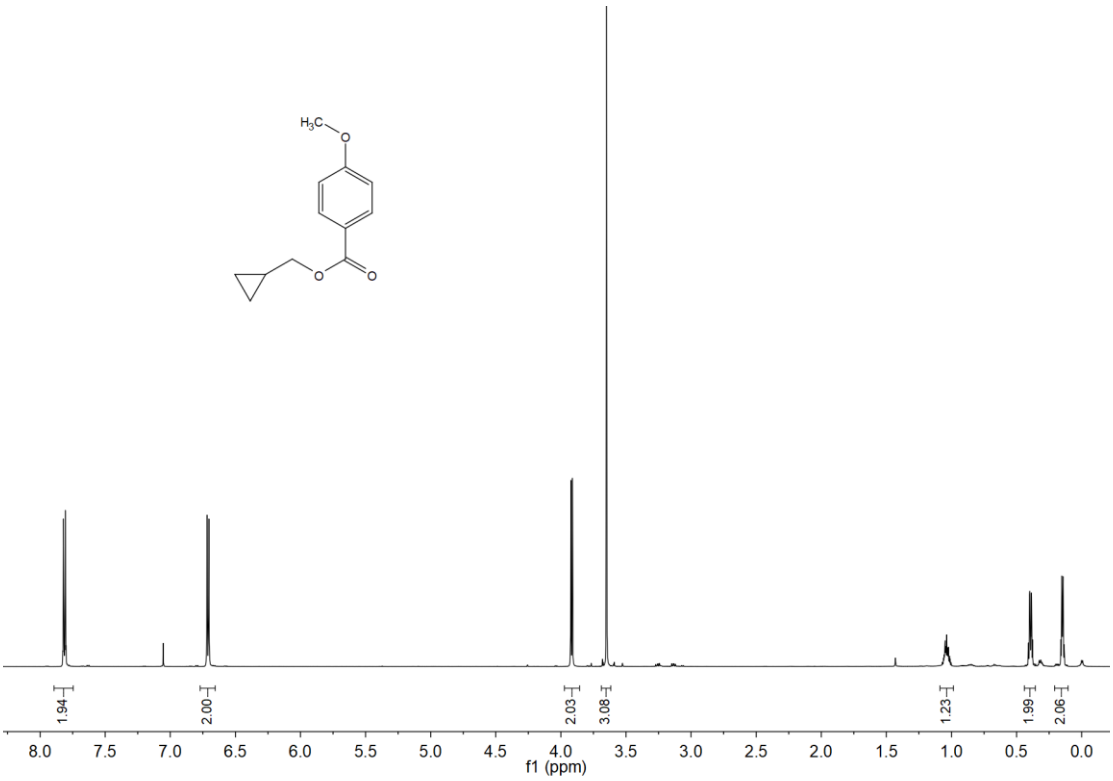

^{13}C NMR (**29c**)

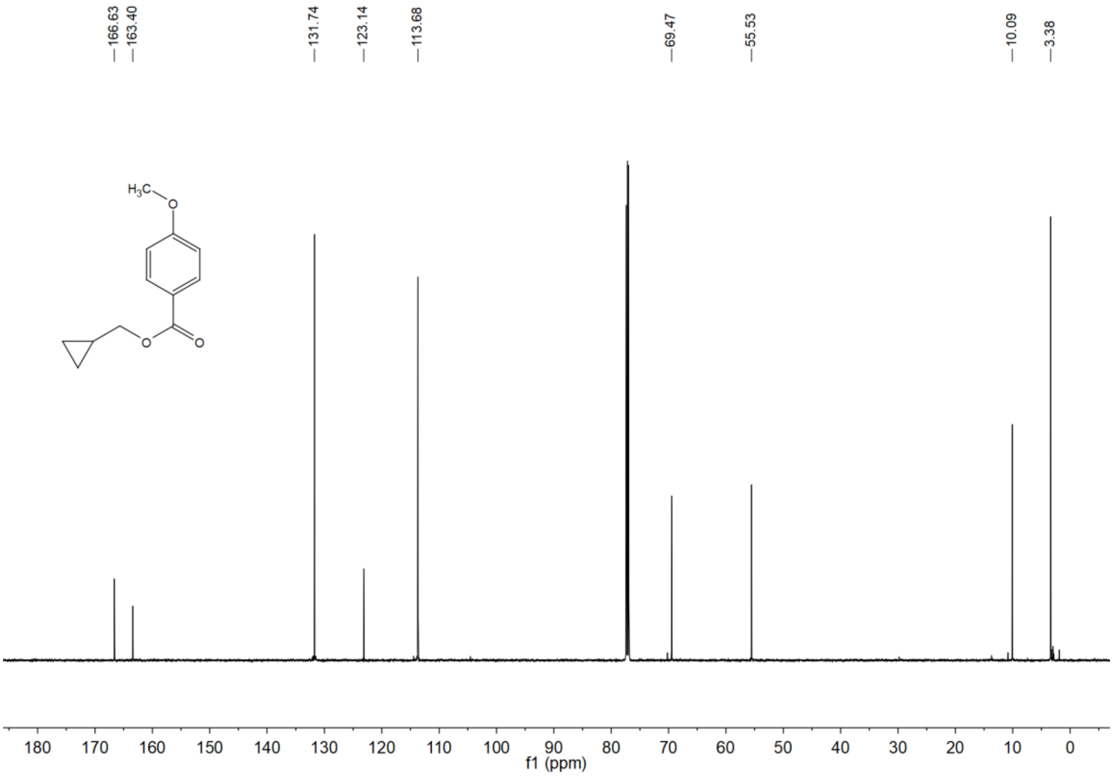

^{1}H NMR (**30c**)

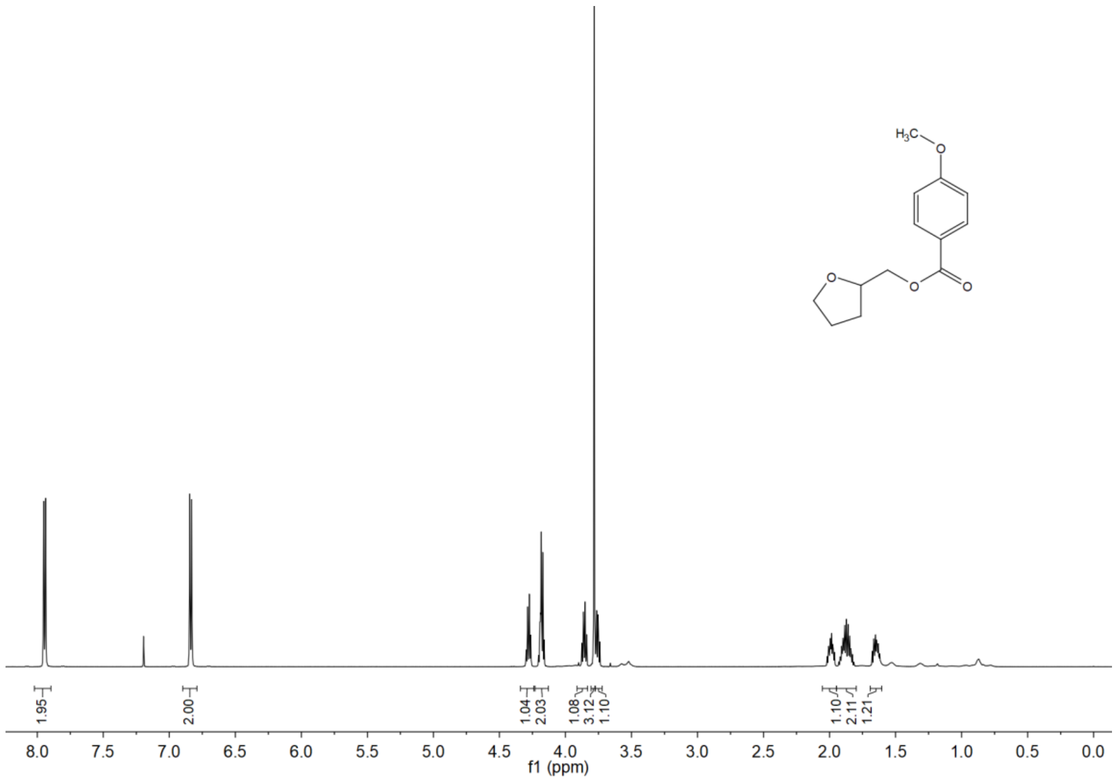

^{13}C NMR (**30c**)

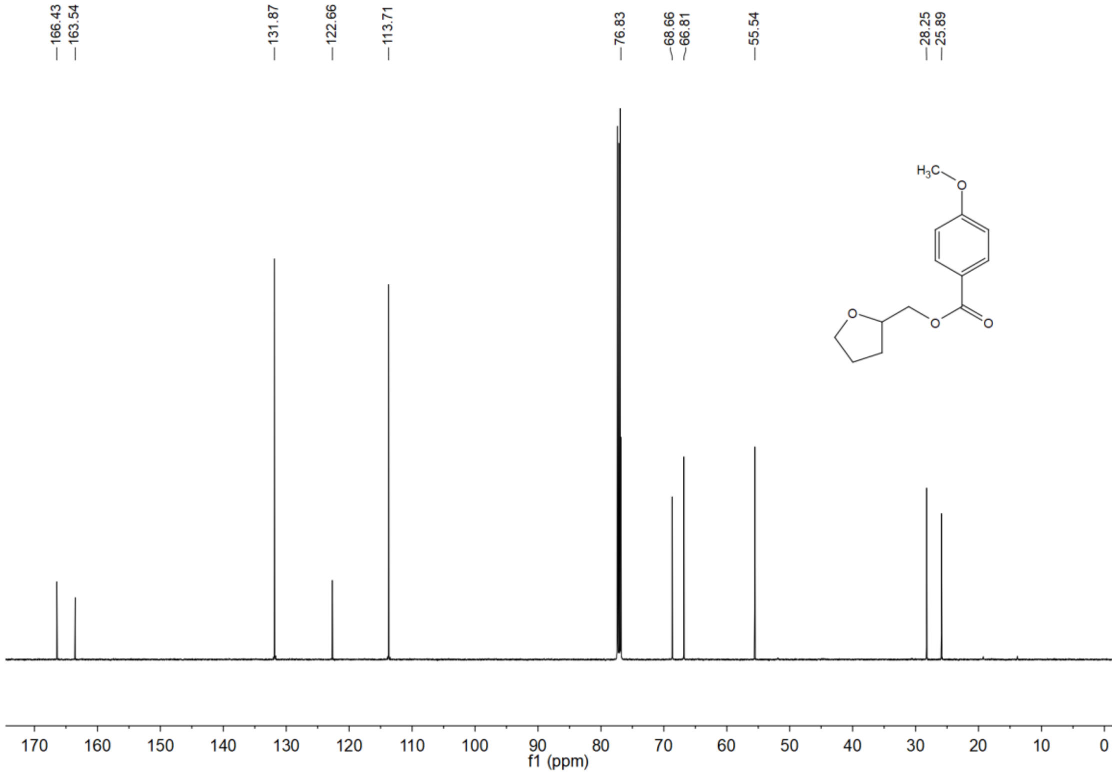

^{1}H NMR (**31c**)

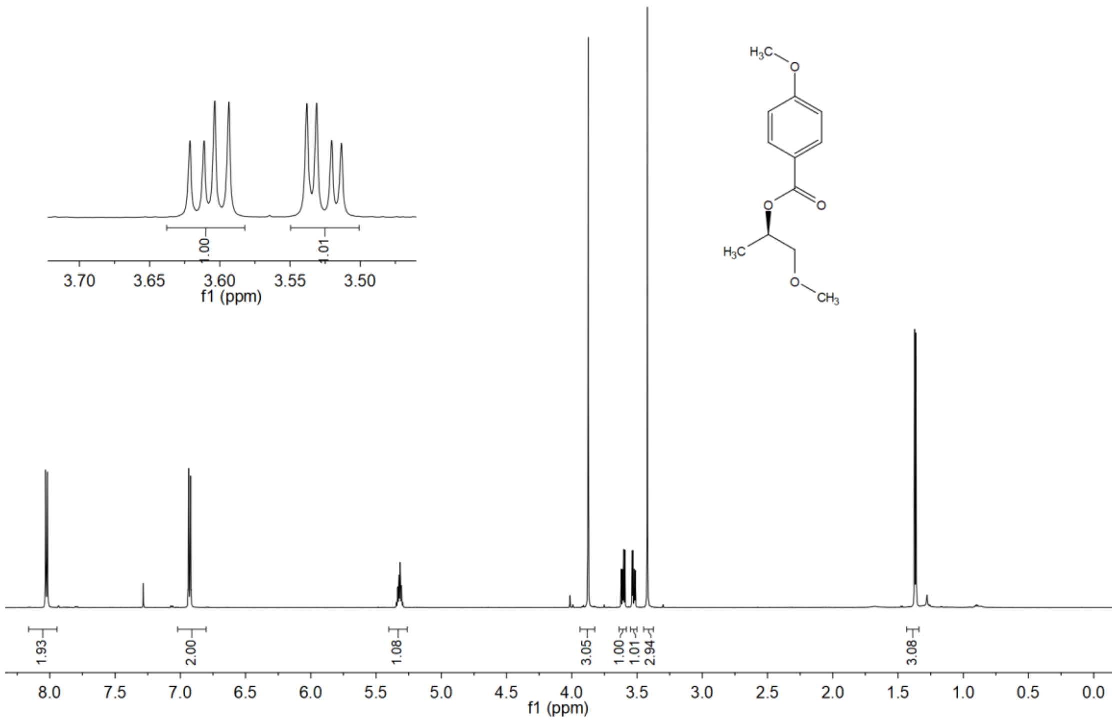

^{13}C NMR (**31c**)

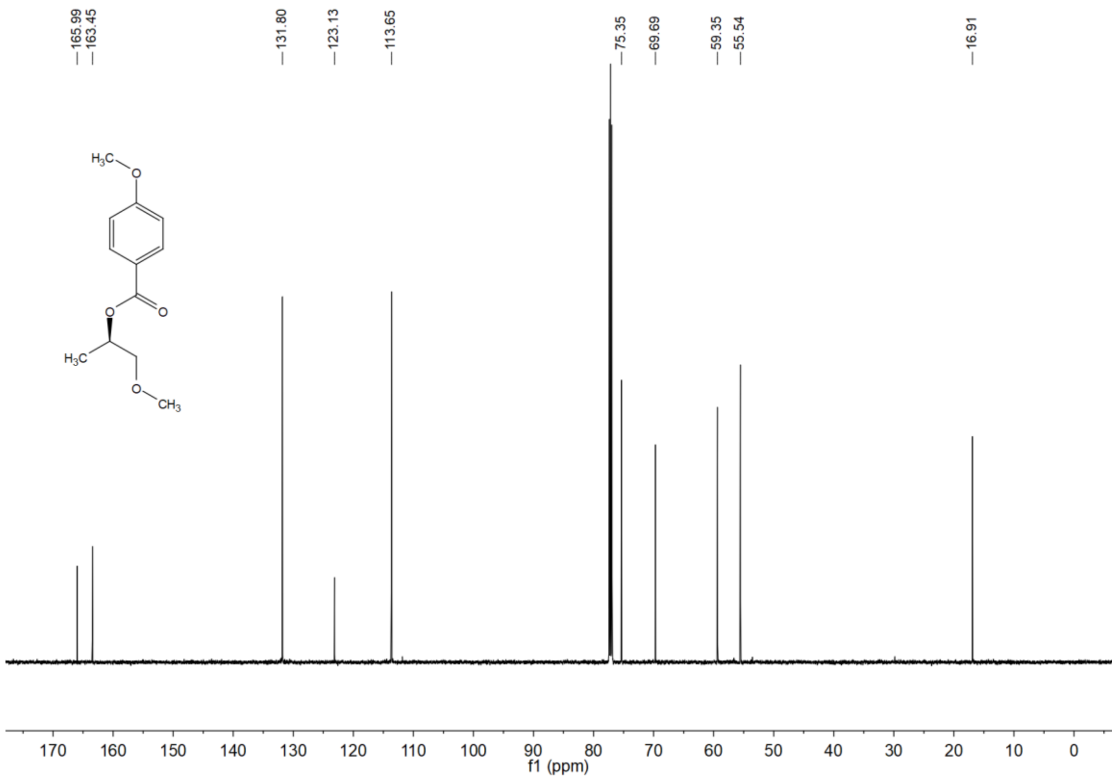

^{1}H NMR (**32c**)

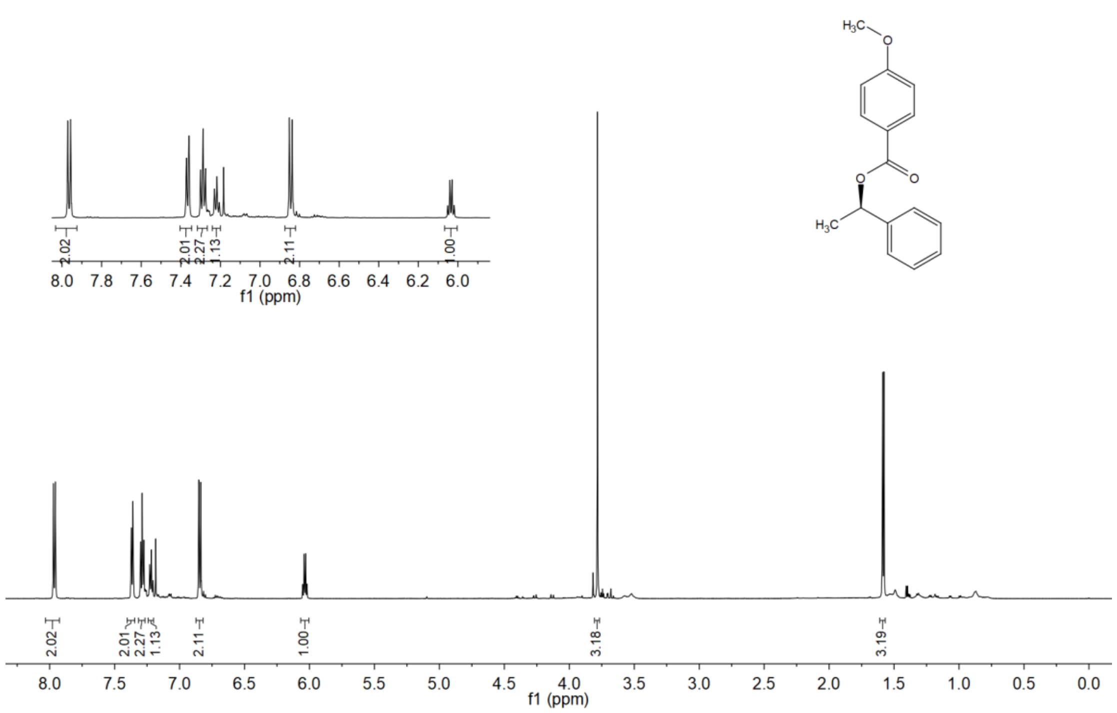

^{13}C NMR (**32c**)

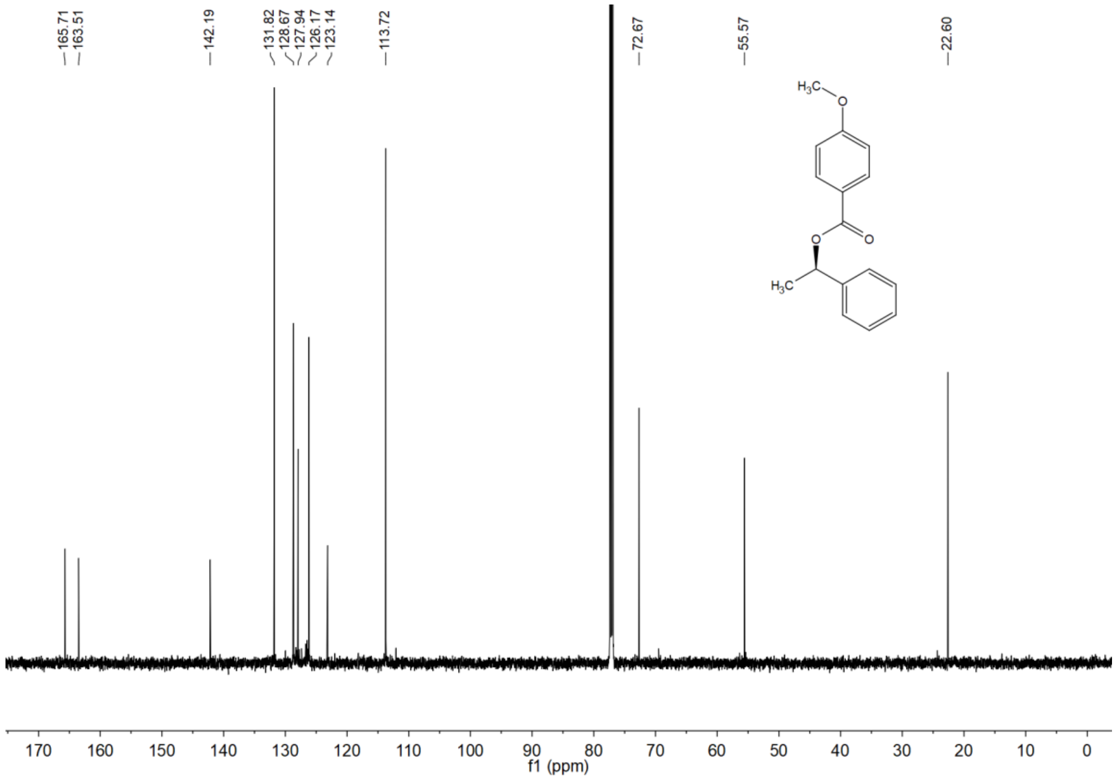

^{1}H NMR (**1e**)

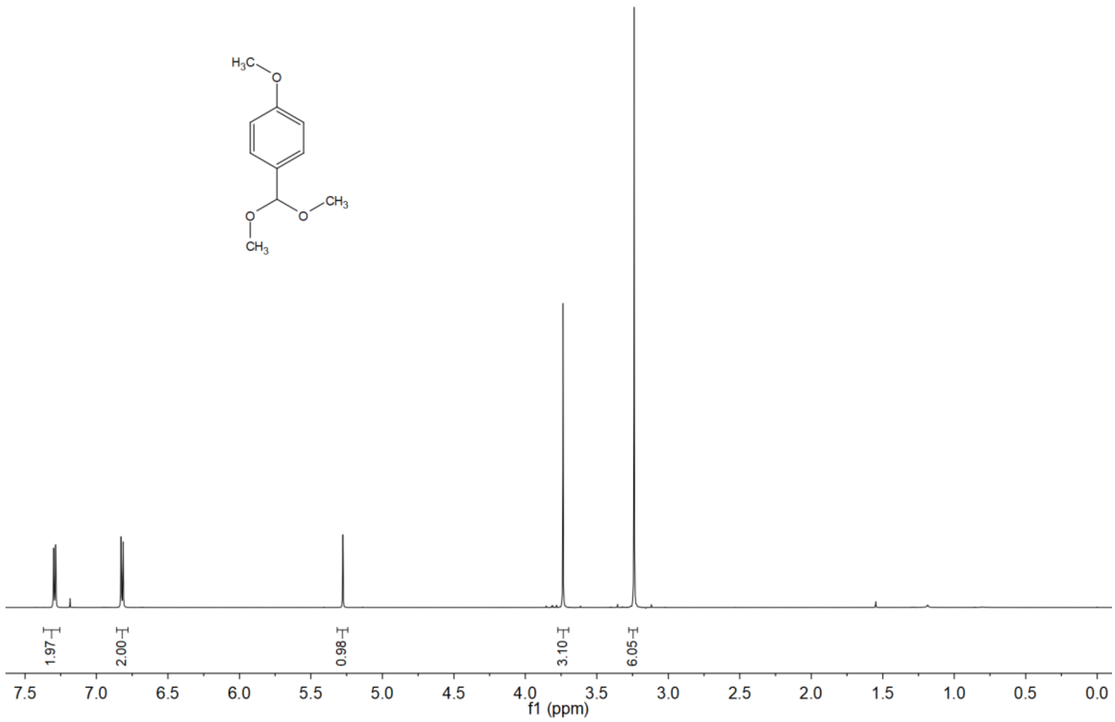

^{13}C NMR (**1e**)

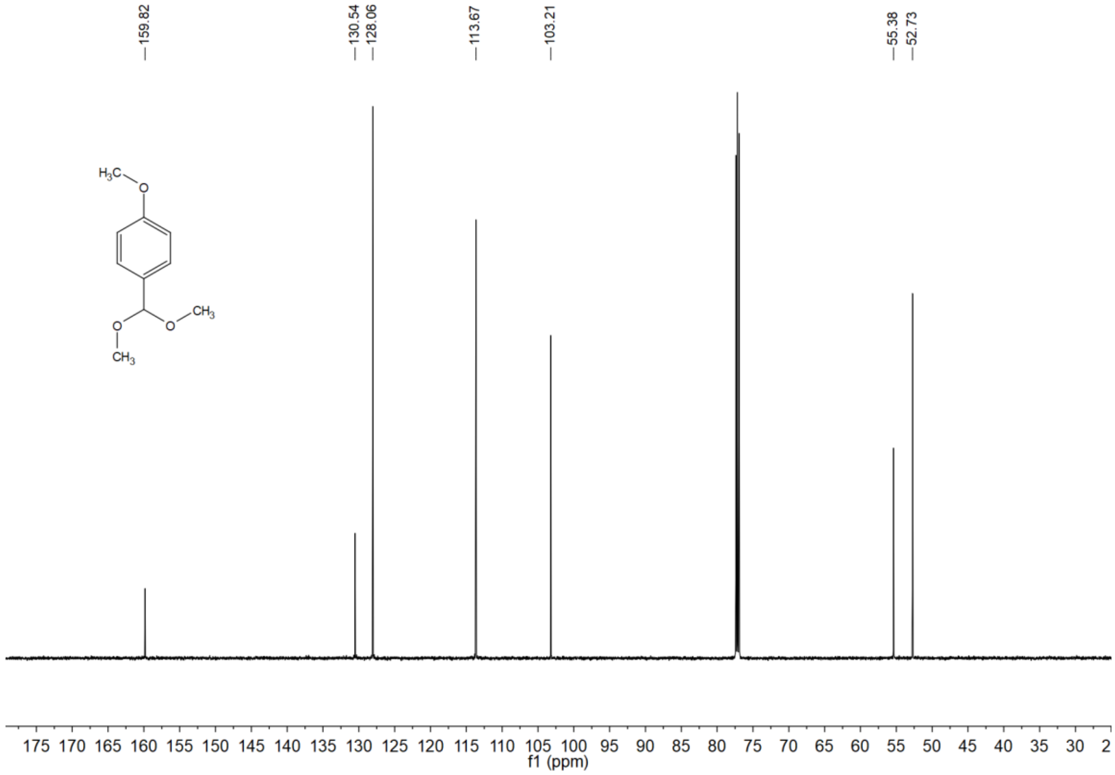

^{1}H NMR (**2e**)

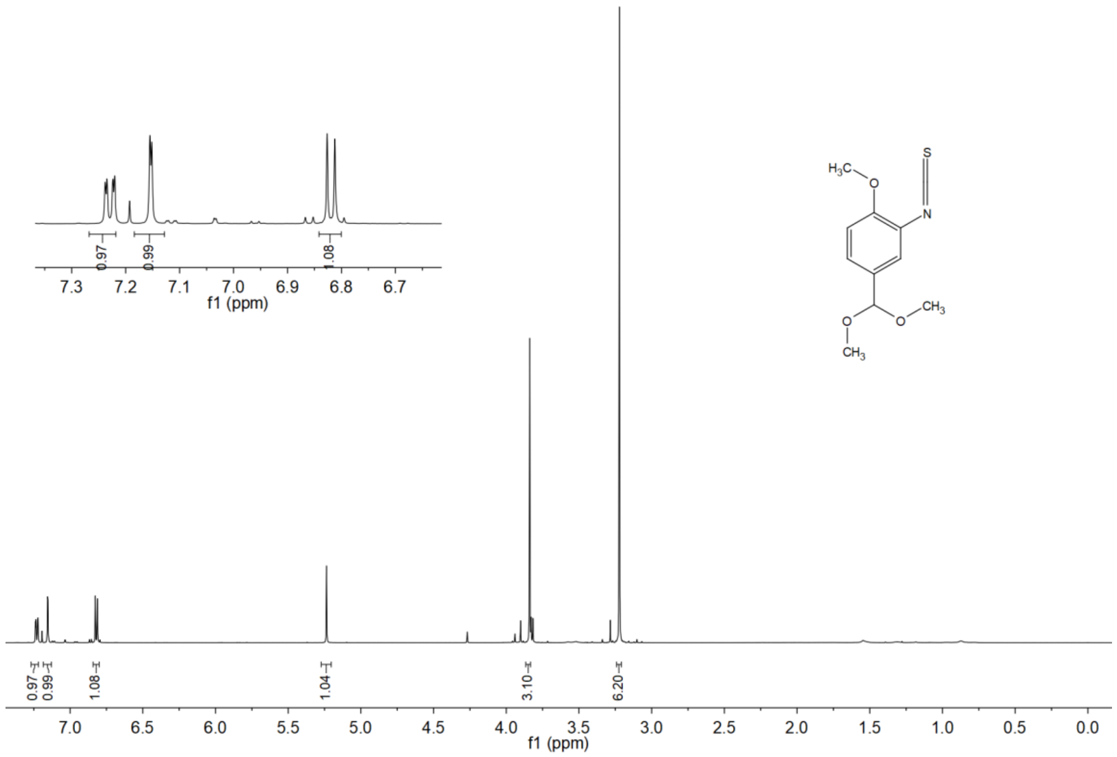

^{13}C NMR (**2e**)

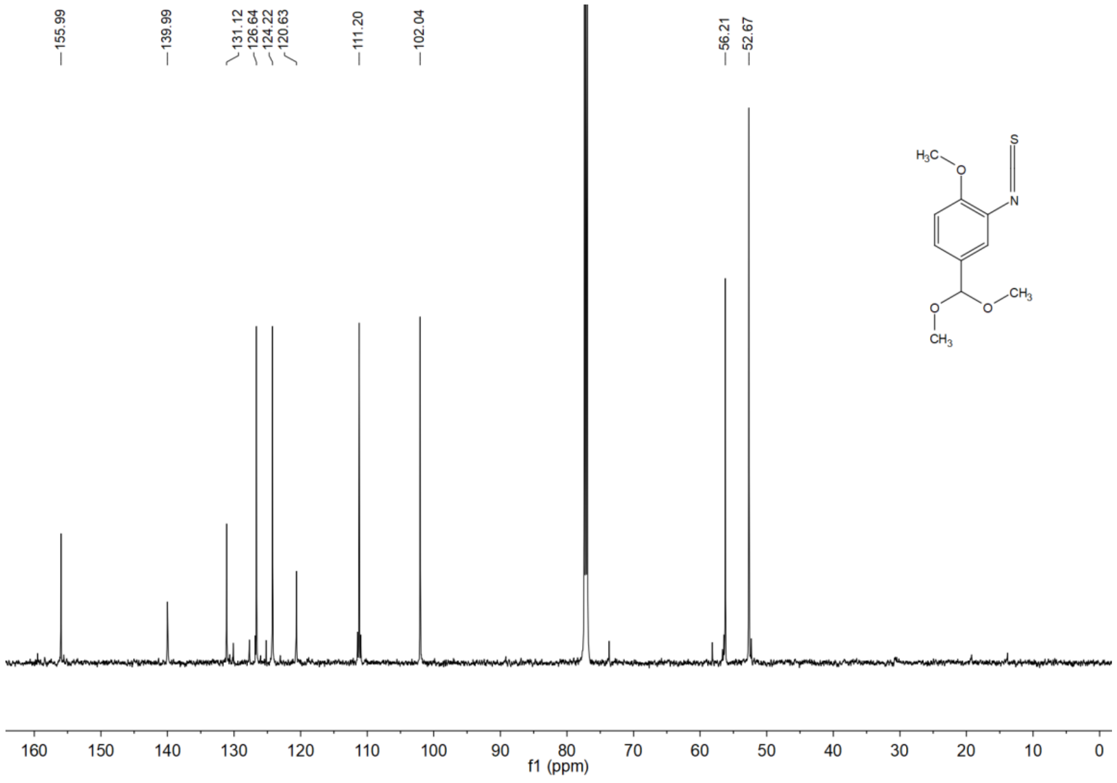

^{1}H NMR (**3e**)

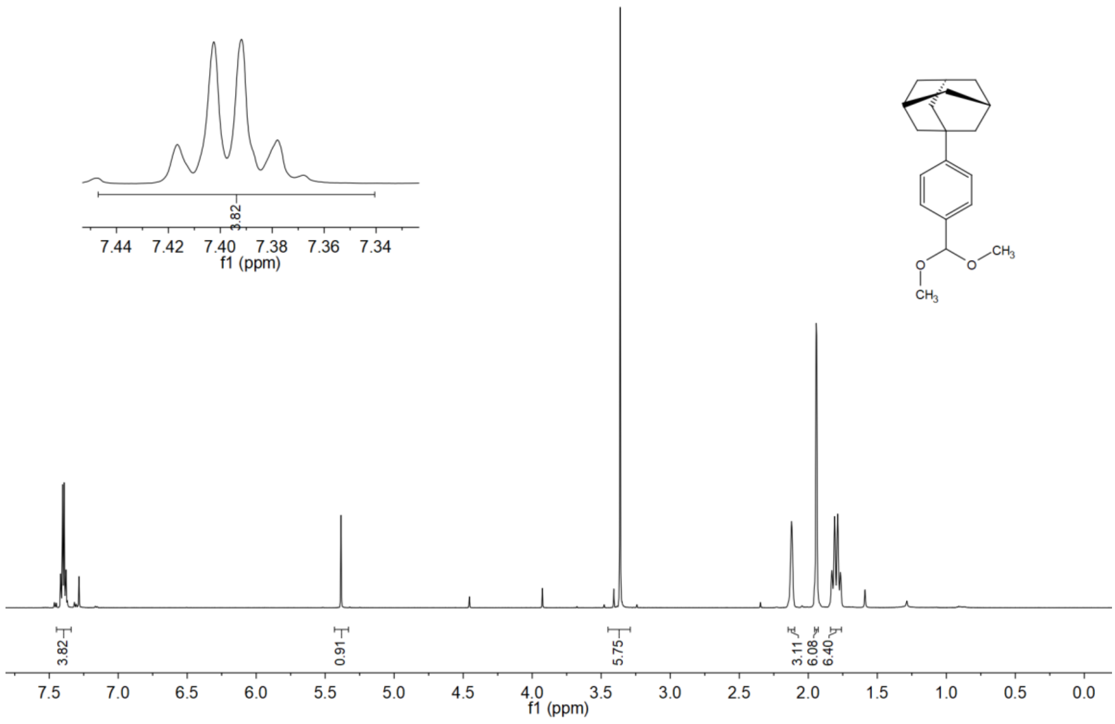

^{13}C NMR (**3e**)

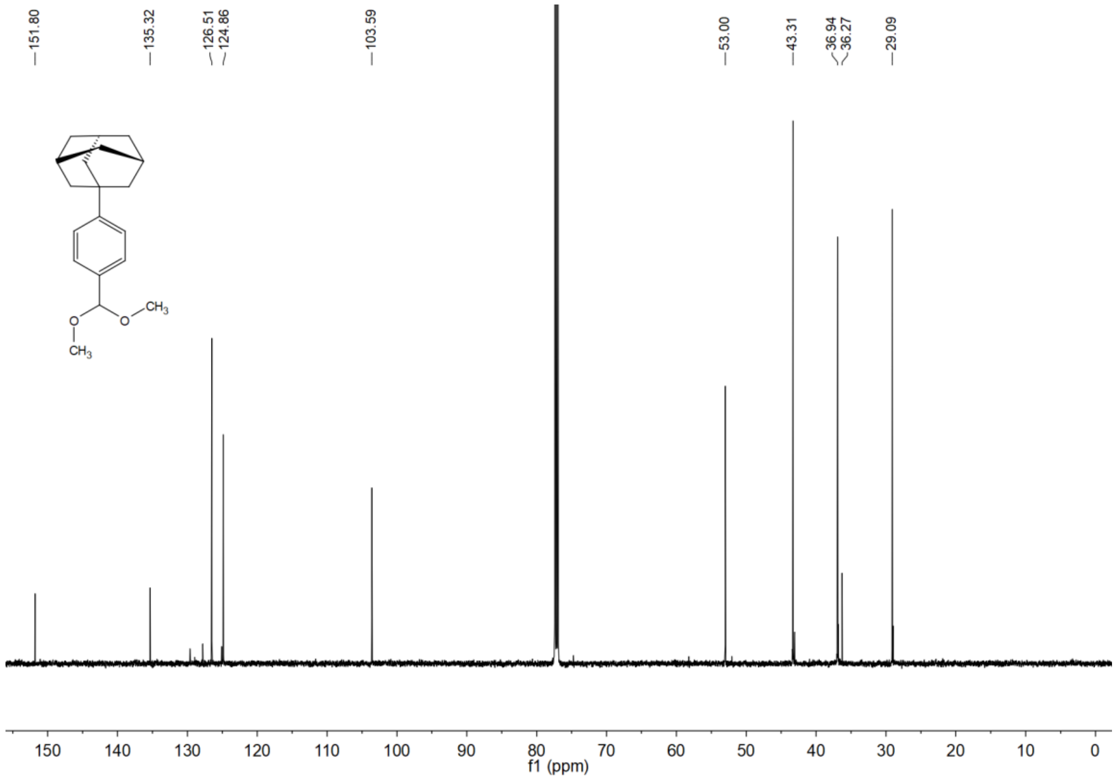

^{1}H NMR (**4e**)

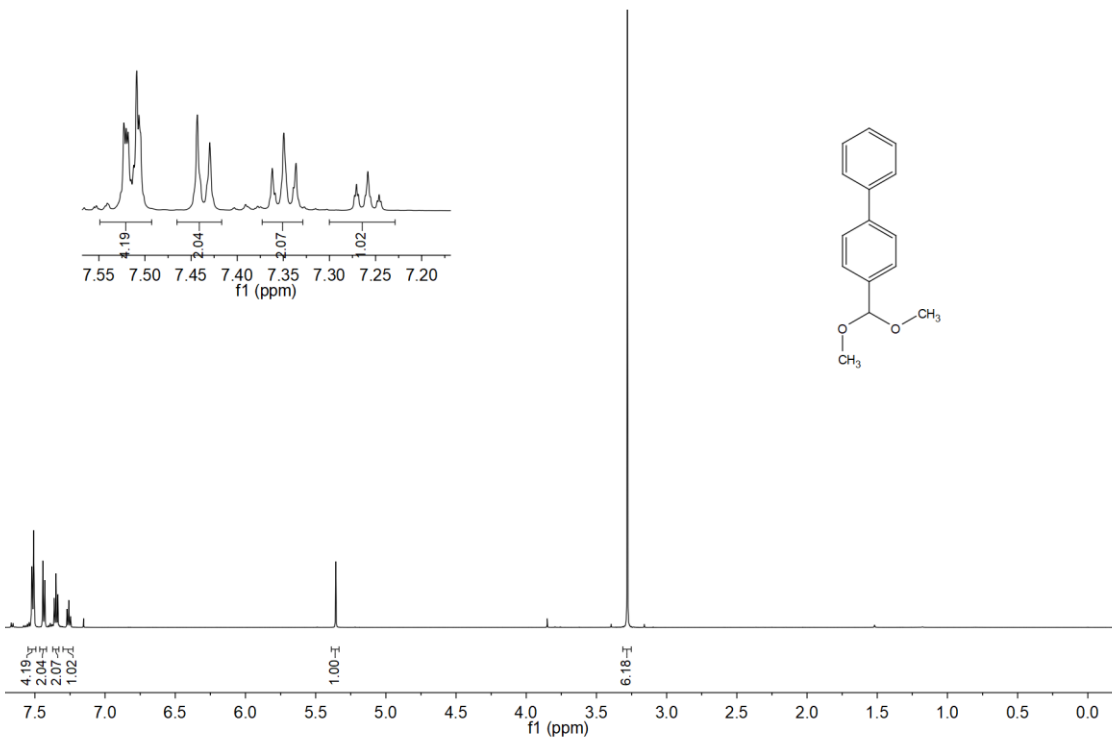

^{13}C NMR (**4e**)

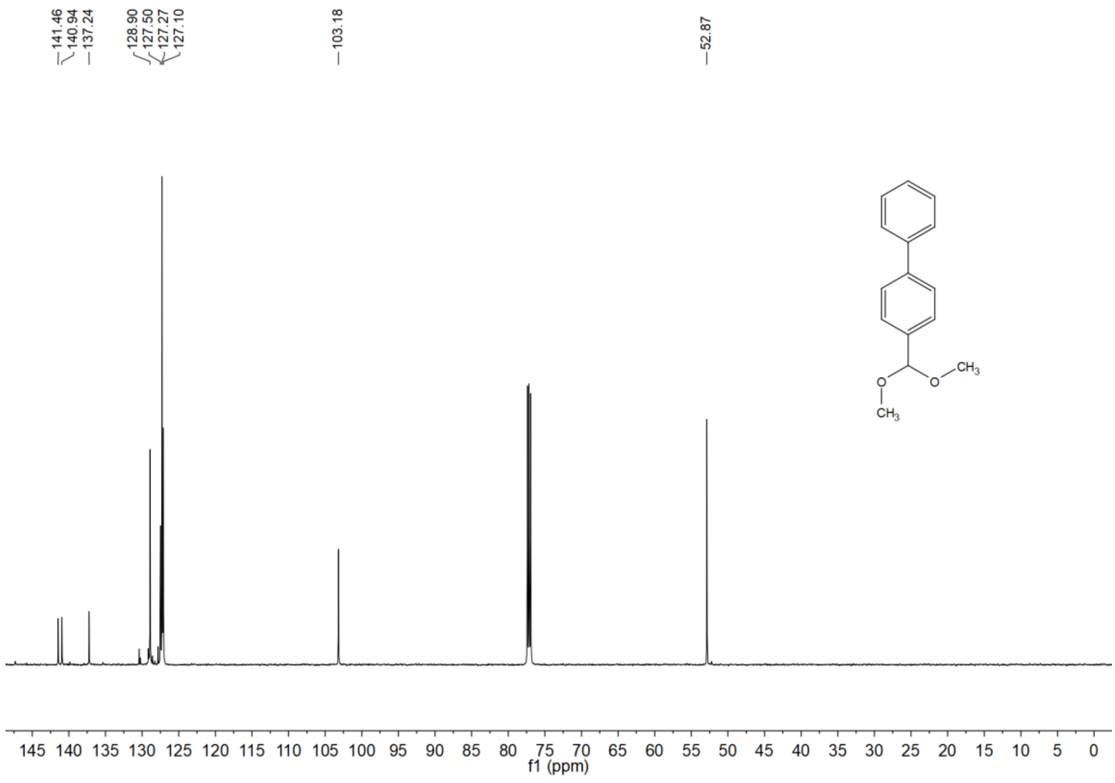

^{1}H NMR (**5e**)

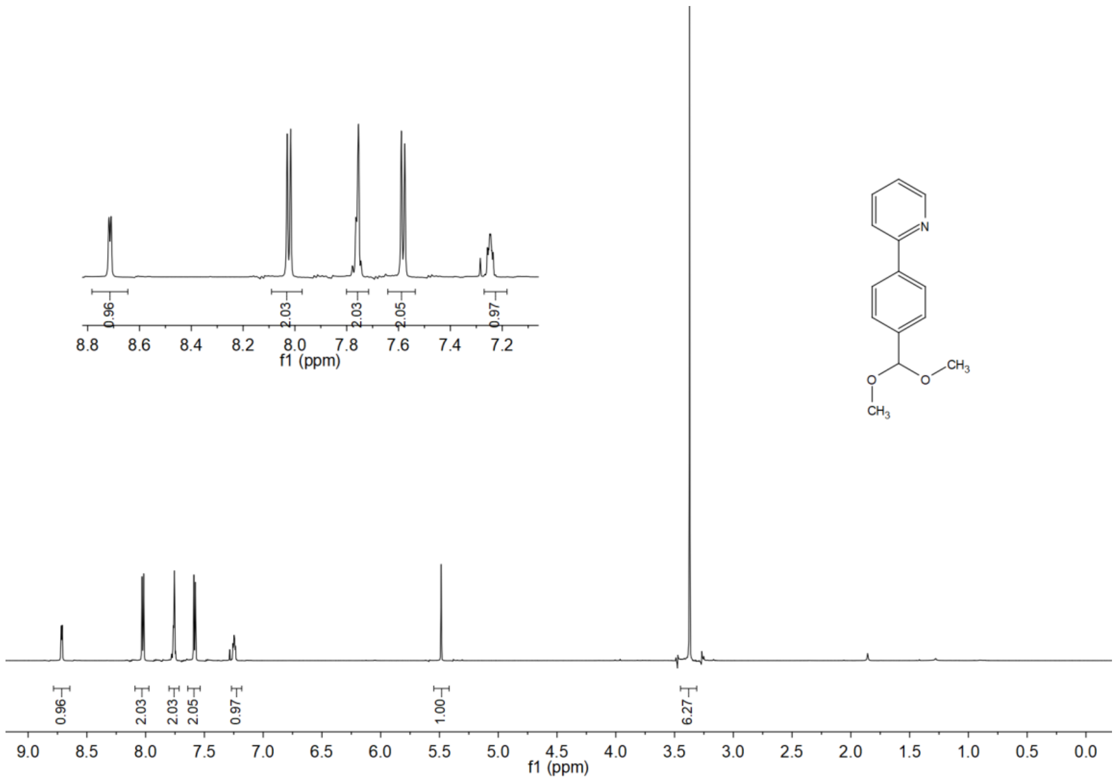

^{13}C NMR (**5e**)

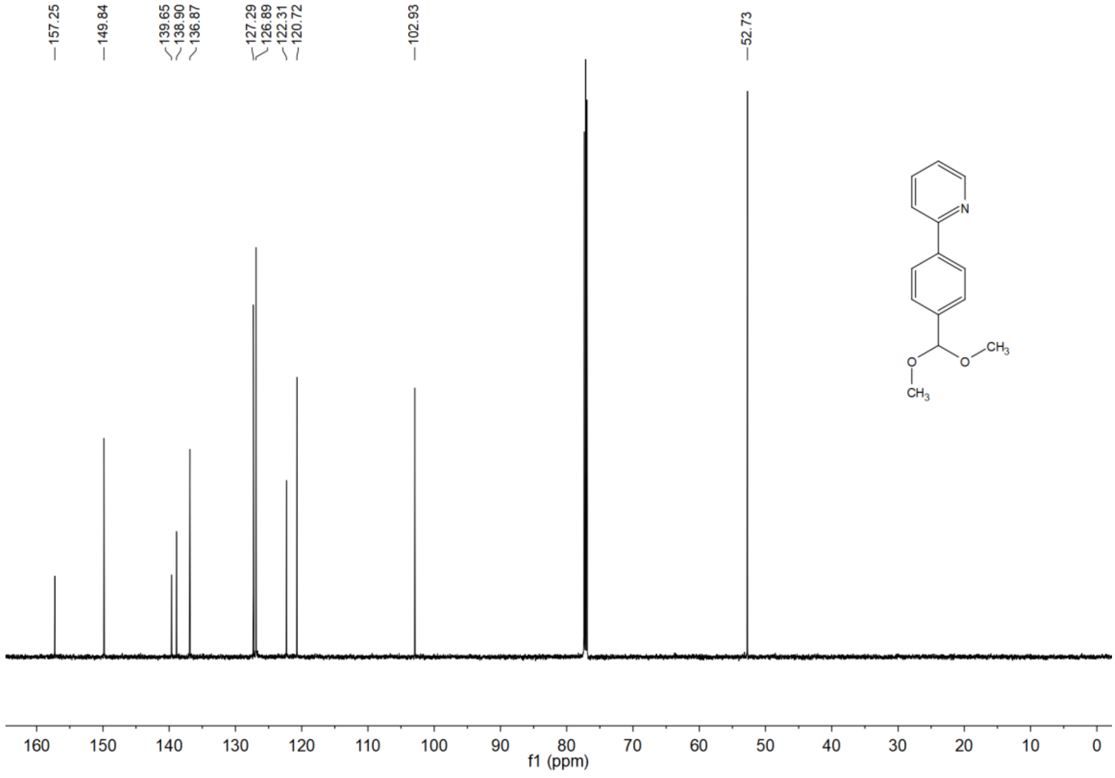

^{1}H NMR (**33c**)

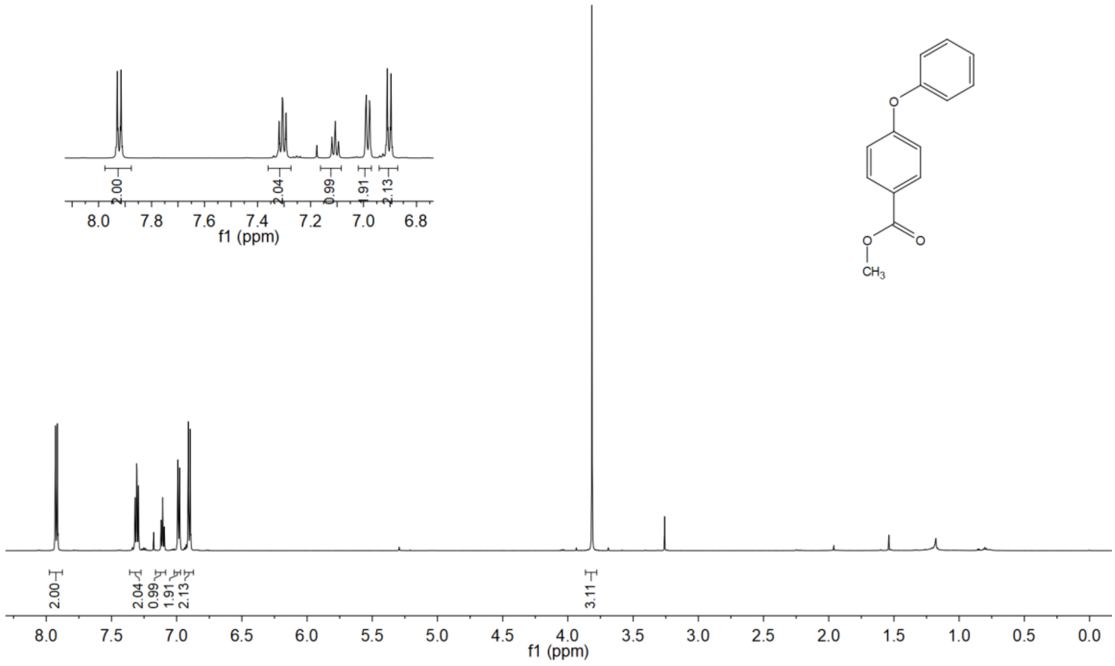

^{13}C NMR (**33c**)

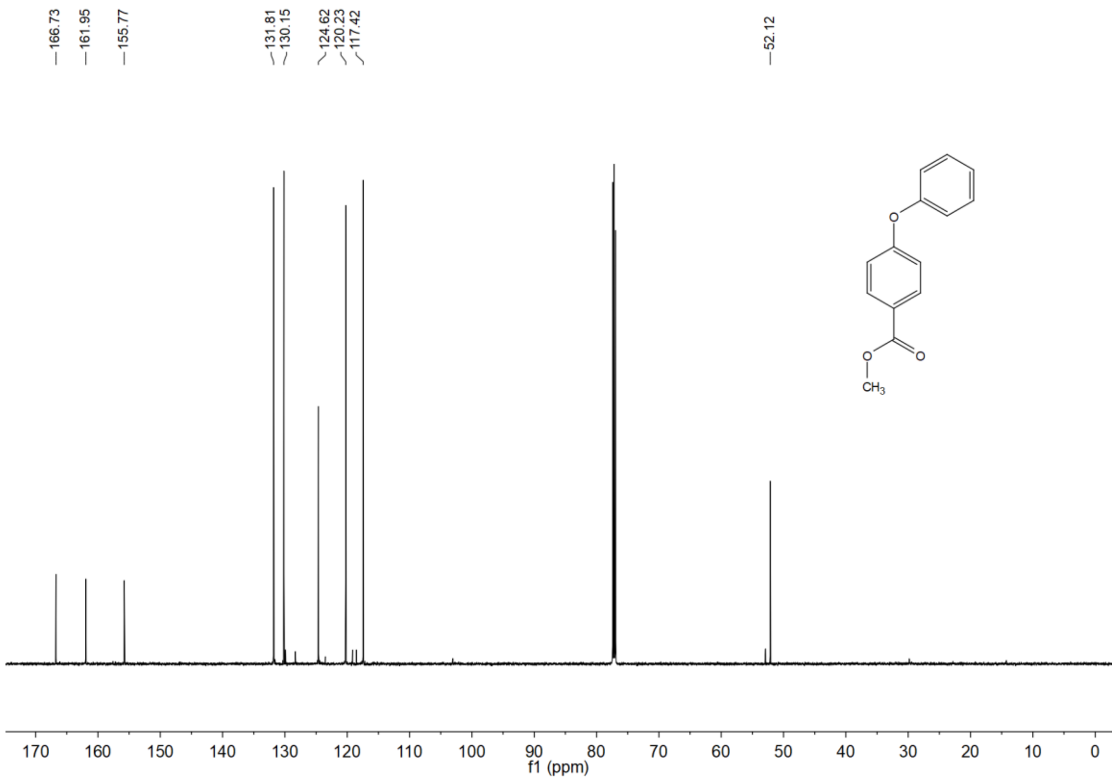

^{1}H NMR (**34c**)

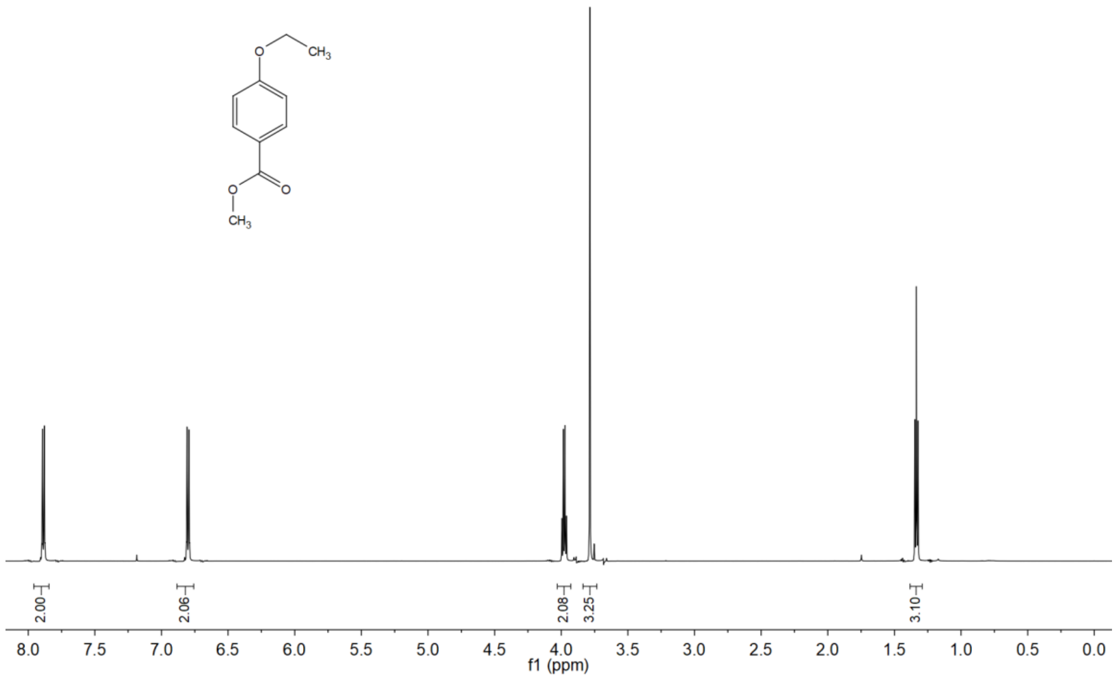

^{13}C NMR (**34c**)

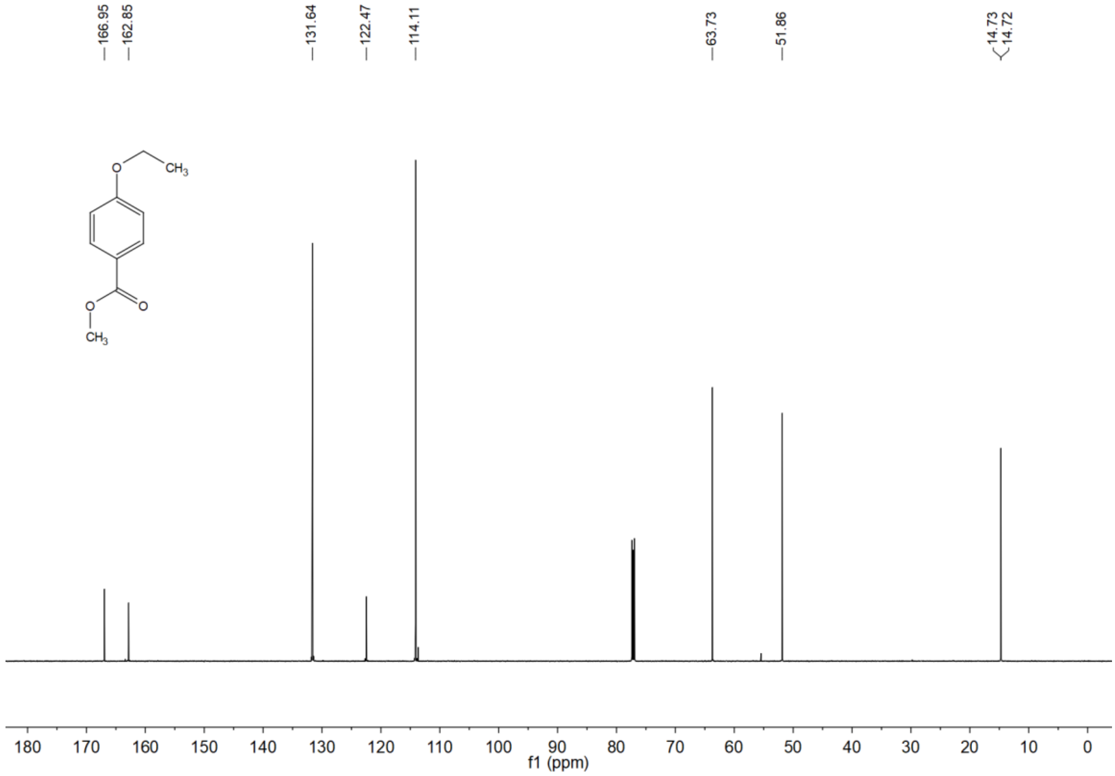

^{1}H NMR (**35c**)

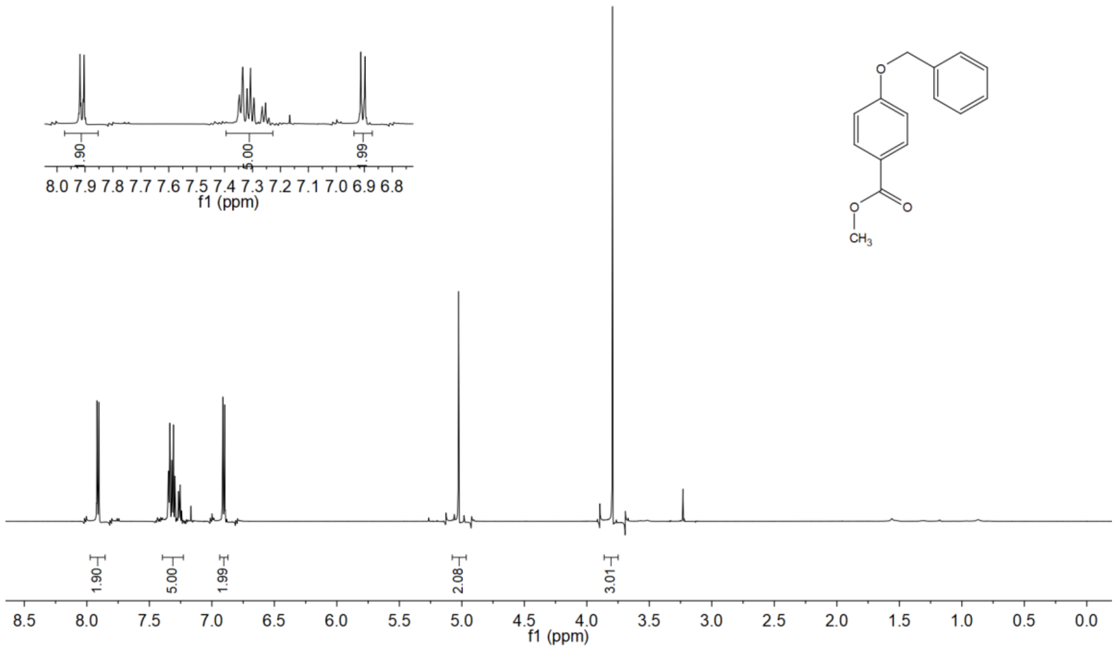

^{13}C NMR (**35c**)

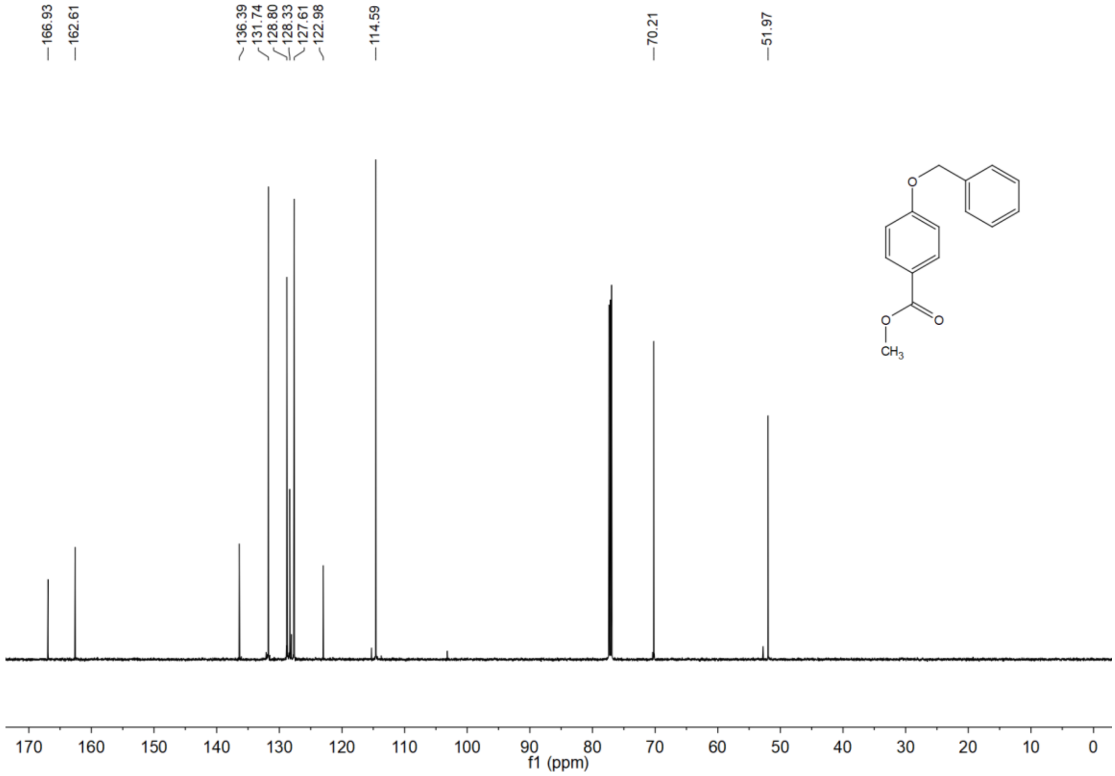

^{1}H NMR (**36c**)

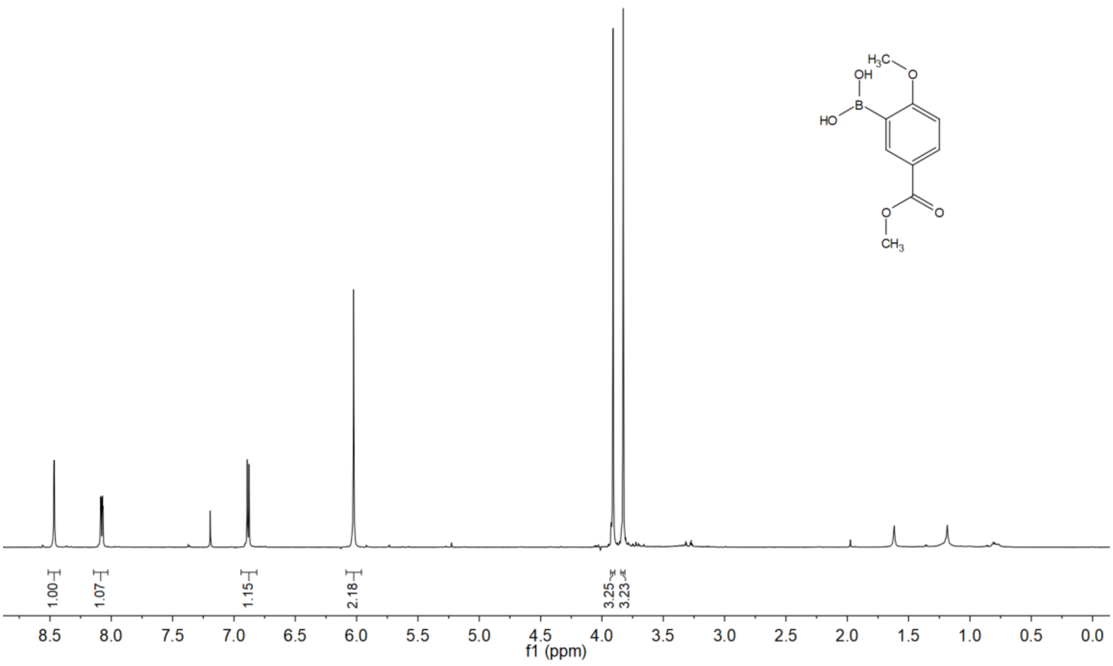

^{13}C NMR (**36c**)

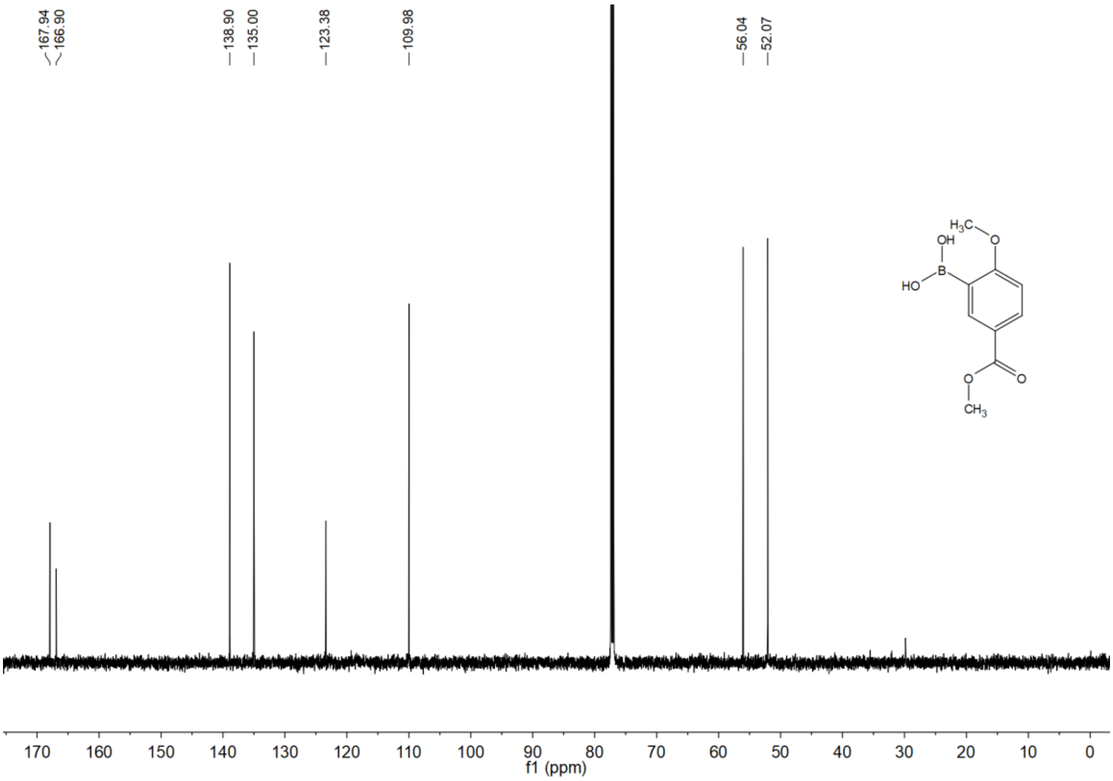

^{1}H NMR (**37c**)

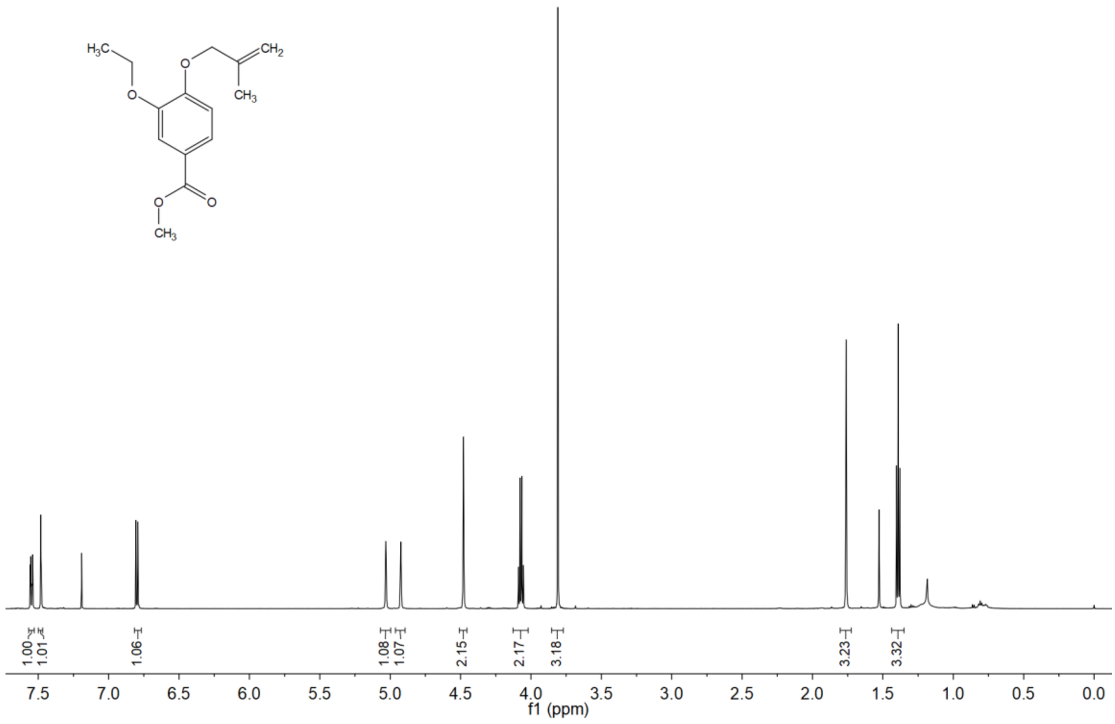

^{13}C NMR (**37c**)

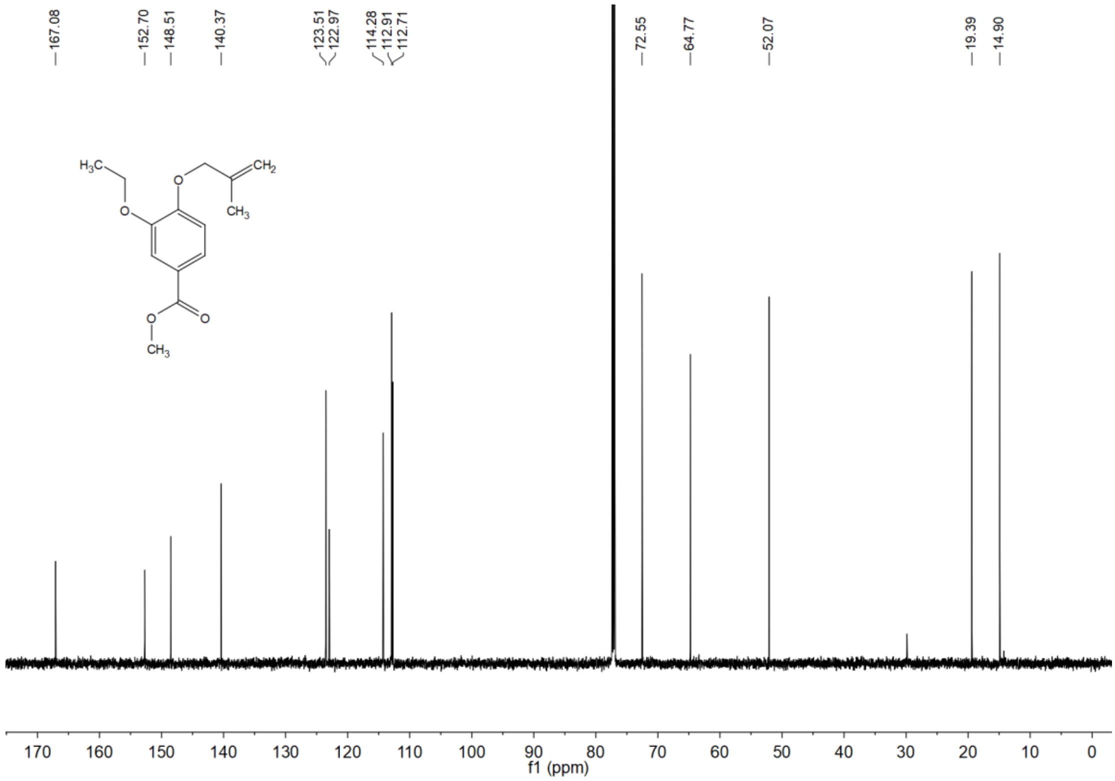

^{1}H NMR (**38c**)

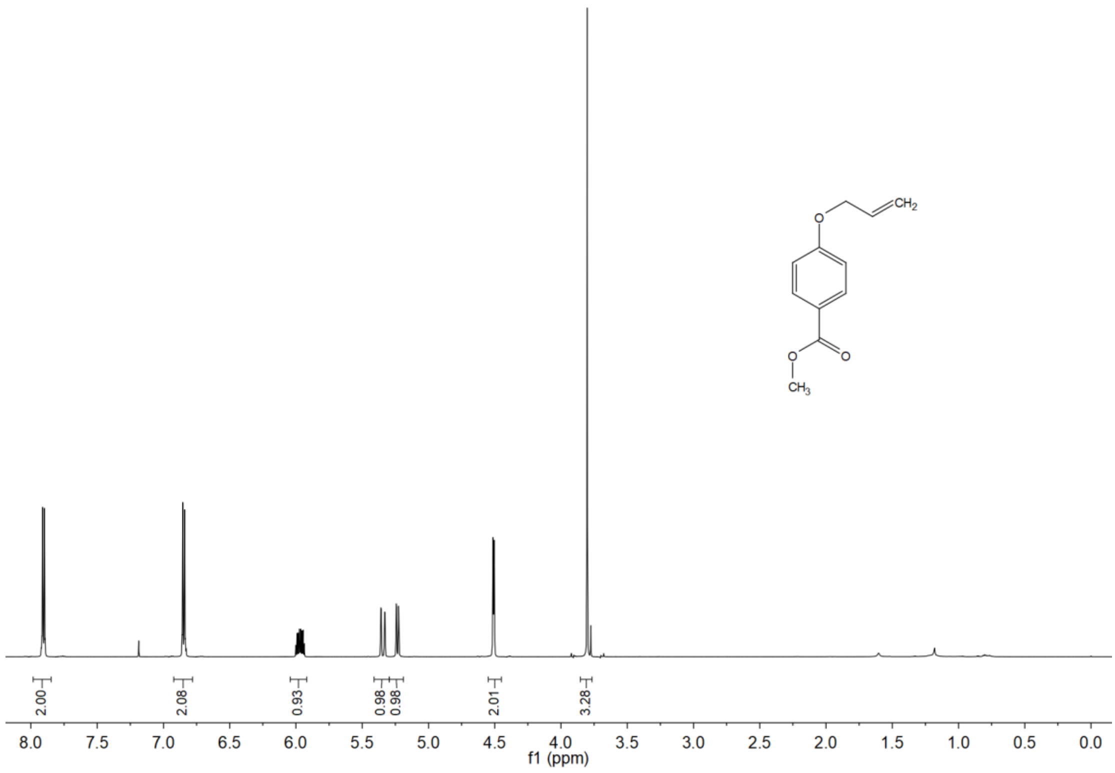

^{13}C NMR (**38c**)

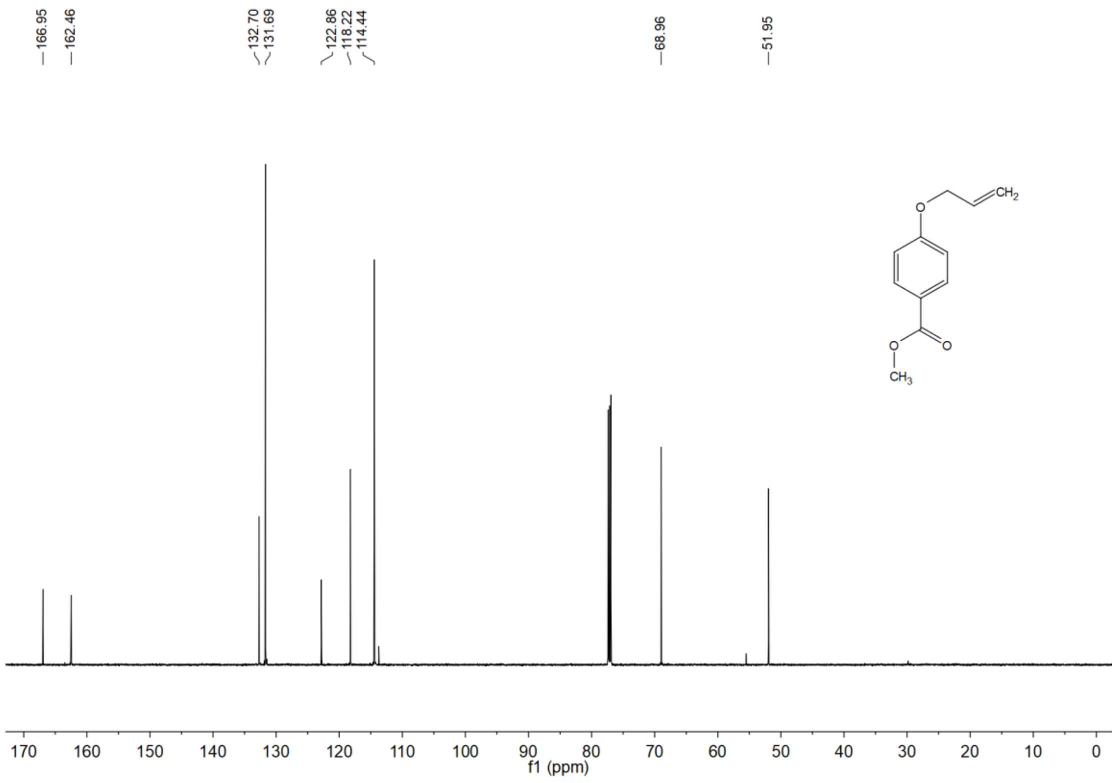

^{1}H NMR (**39c**)

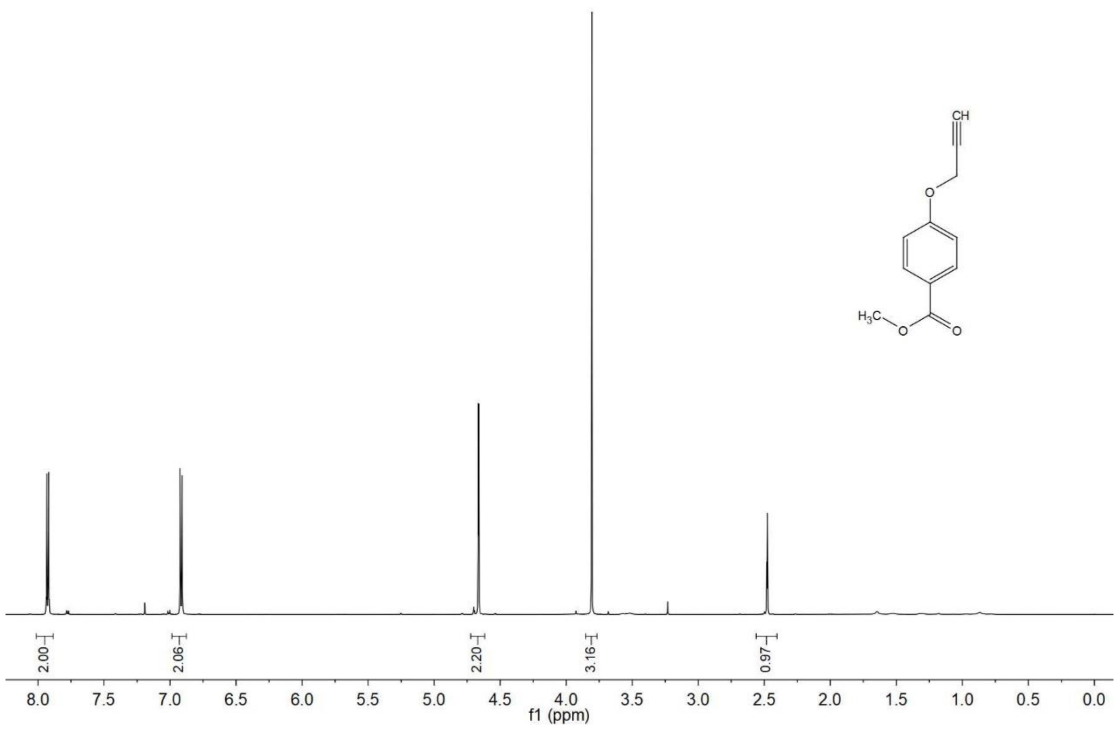

^{13}C NMR (**39c**)

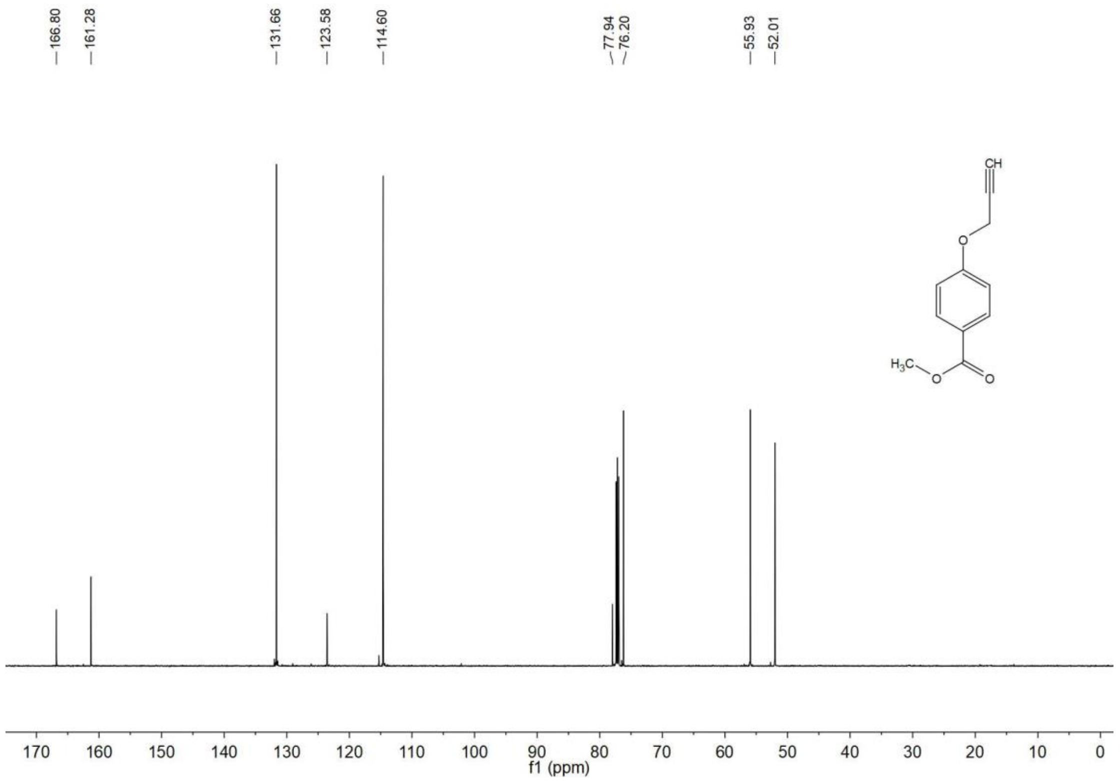

^{1}H NMR (**40c**)

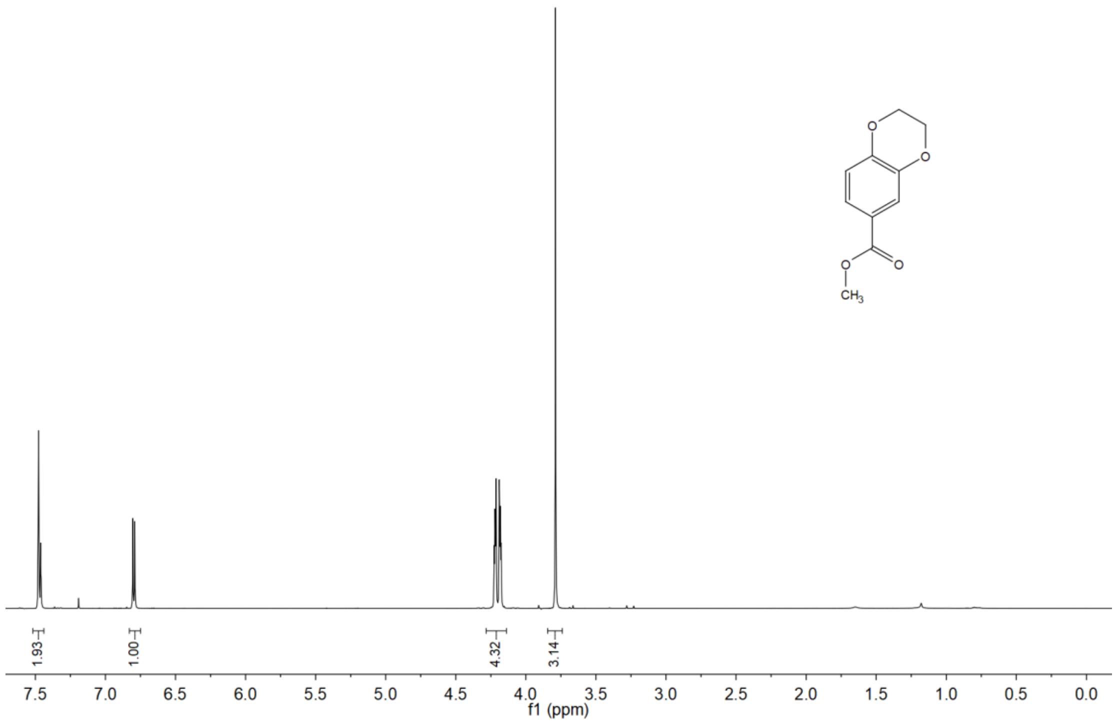

^{13}C NMR (**40c**)

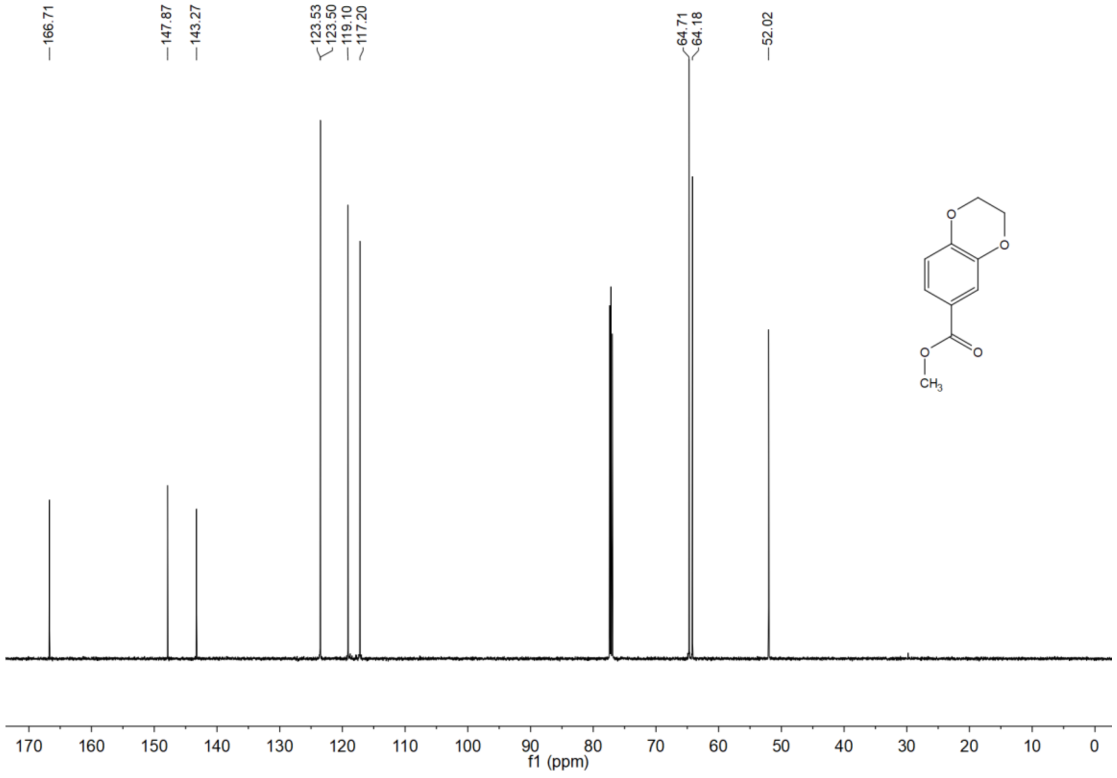

^{1}H NMR (**41c**)

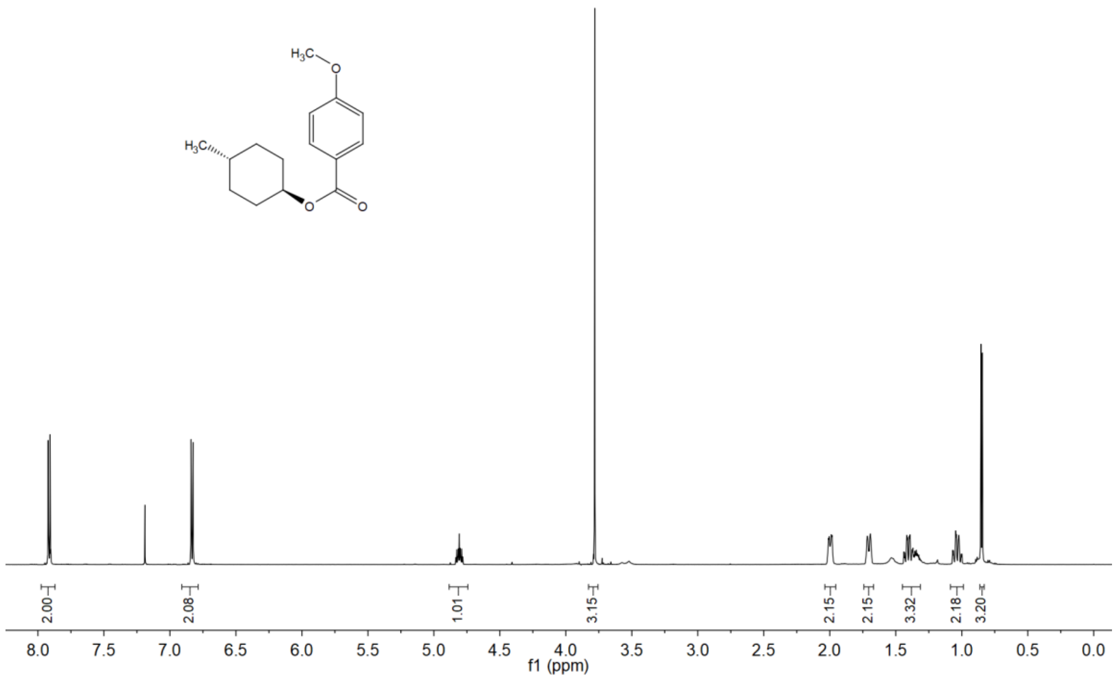

^{13}C NMR (**41c**)

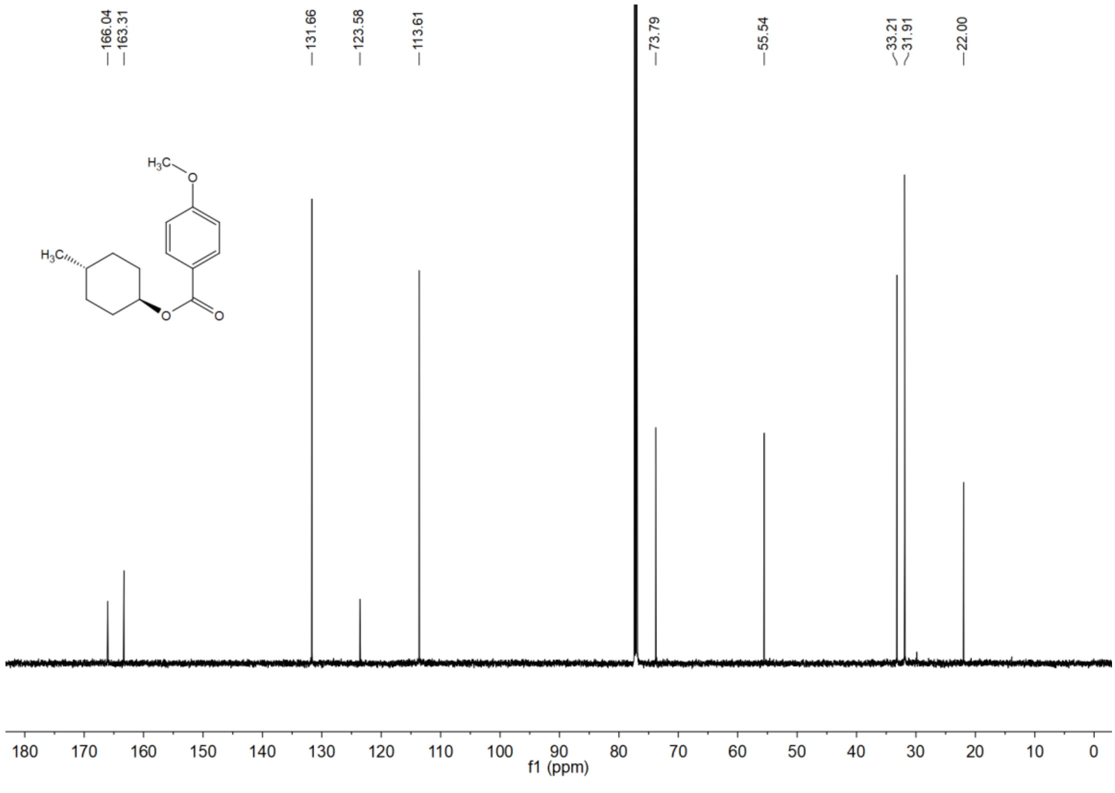

^{1}H NMR (**42c**)

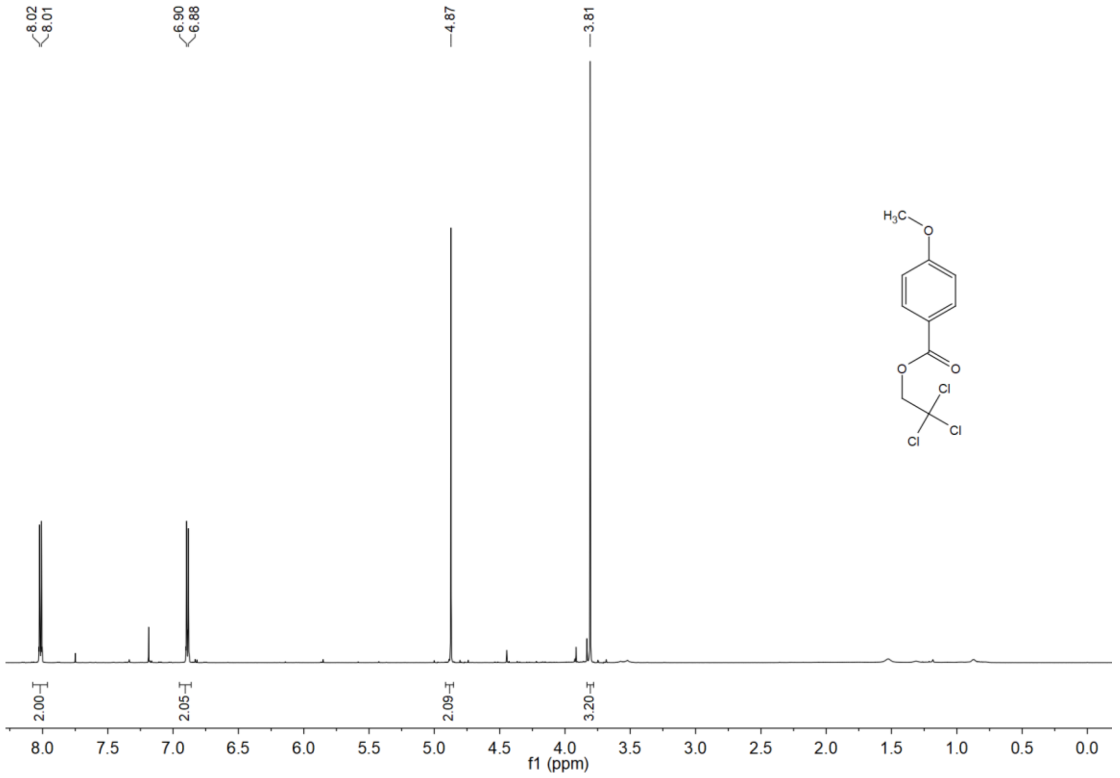

^{13}C NMR (**42c**)

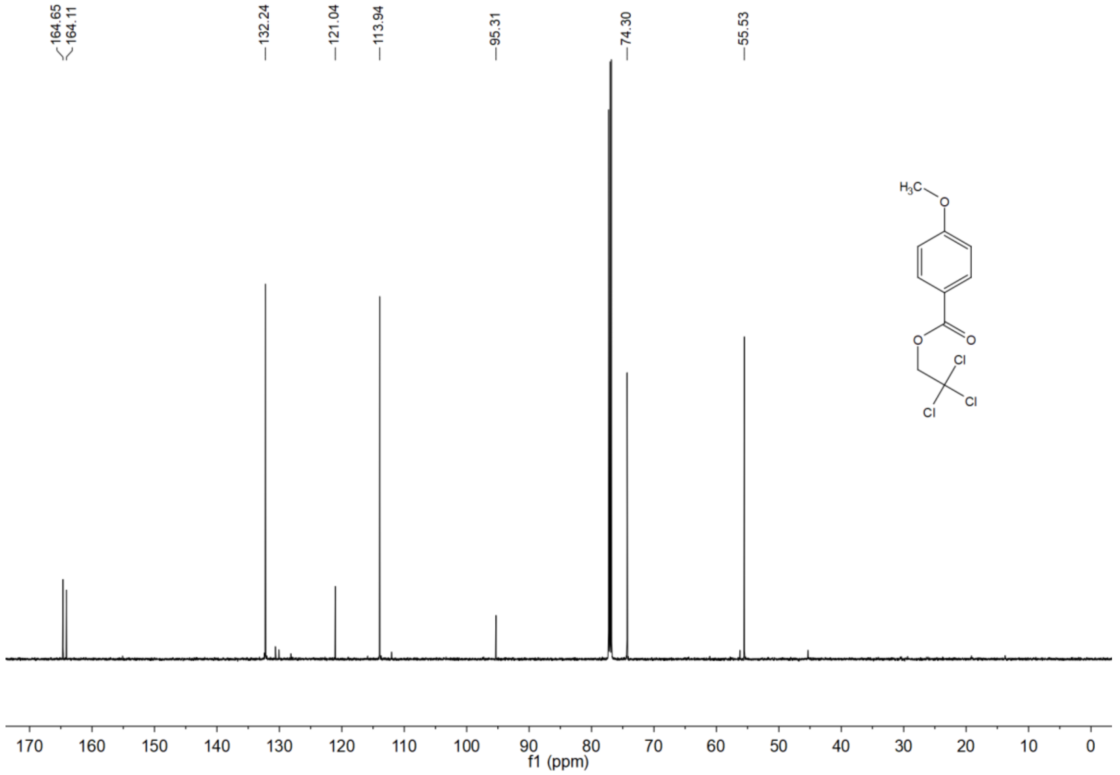

HPLC (**31c**)

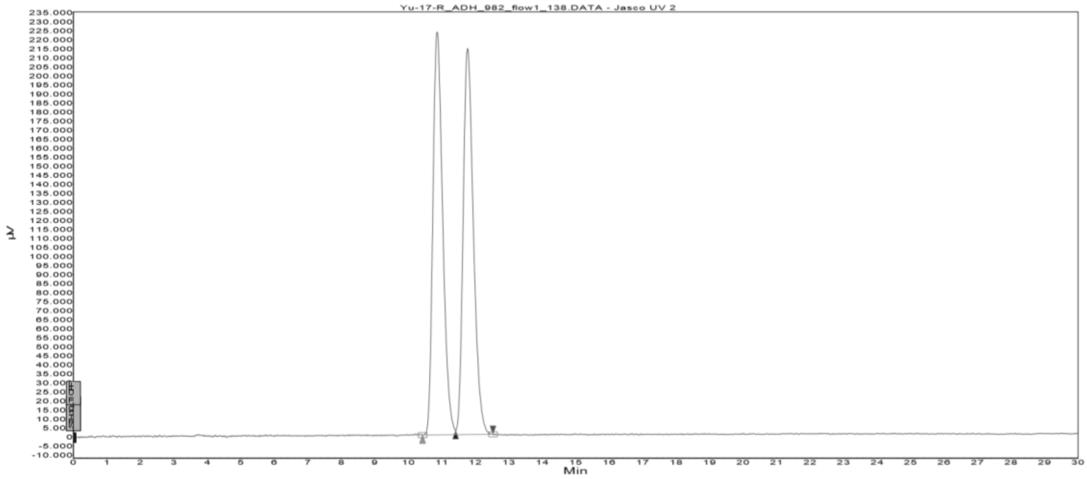

Index	Start	Time	End	Area %
	[Min]	[Min]	[Min]	[%]
1	10,427	10,867	11,415	50,021
2	11,415	11,775	12,528	49,979
Total				100,000

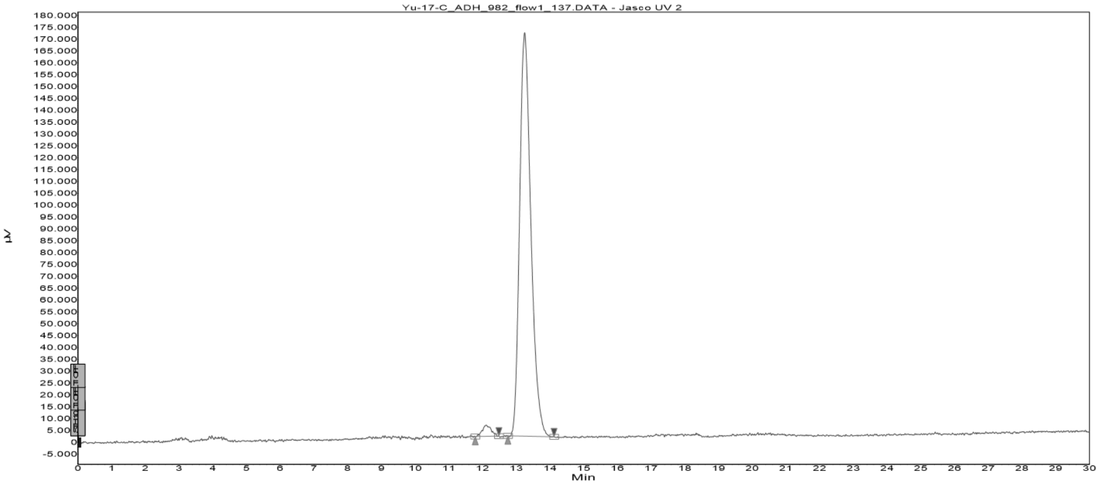

Index	Start	Time	End	Area %
	[Min]	[Min]	[Min]	[%]
1	11,785	12,100	12,488	2,132
2	12,751	13,250	14,122	97,868
Total				100,000

HPLC (**32c**)

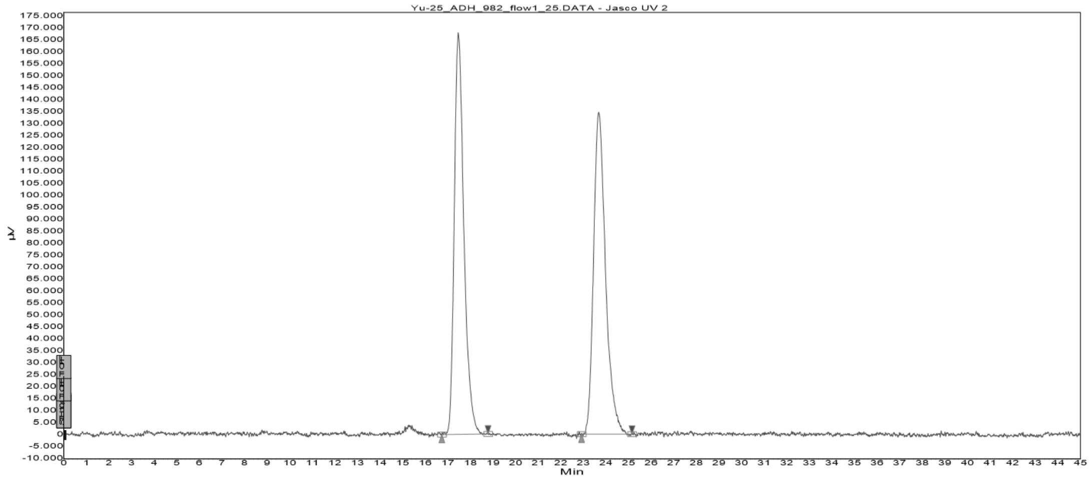

Index	Start	Time	End	Area %
	[Min]	[Min]	[Min]	[%]
1	16,736	17,458	18,781	50,045
2	22,918	23,675	25,150	49,955
Total				100,000

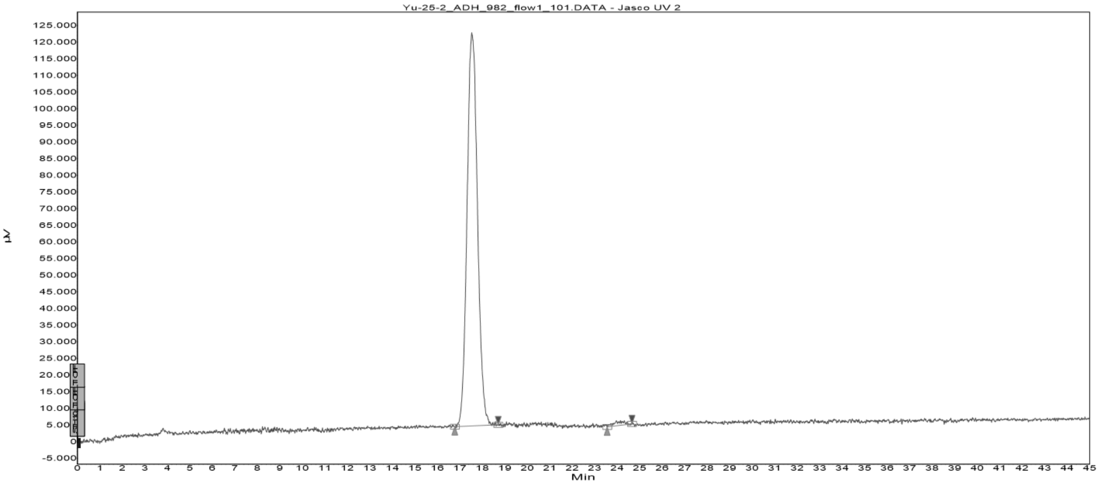

Index	Start	Time	End	Area %
	[Min]	[Min]	[Min]	[%]
1	16,780	17,533	18,711	98,965
2	23,552	23,958	24,655	1,035
Total				100,000
